# Supplementary material for: A conditional protein diffusion model generates artificial programmable endonuclease sequences with enhanced activity
Source: Cell Discov. 2024 Sep 10;10:95. doi: 10.1038/s41421-024-00728-2 (PMC11385924; doi:10.1038/s41421-024-00728-2)
Supplement: Supplementary file 1 — Supplementary information [file 41421_2024_728_MOESM1_ESM.pdf]

Supplementary Information: Conditional protein diffusion generates artificial programmable endonuclease sequences with enhanced activity

Bingxin Zhou, Lirong Zheng, Banghao Wu, Kai Yi,
Bozitao Zhong, Qian Liu, Pietro Liò, Liang Hong

Supplementary Data

Contents

1 Protein Graph Representation **7**

2 Conditional Protein Denoising Diffusion Method **8**
2.1 Diffusion Process and Generative Denoising Process 8
2.1.1 Diffusion Process 8
2.1.2 Training Denoising Networks 9
2.1.3 Parameterized Generative Process 9
2.2 Protein-Specific Conditions for Model Training 10
2.2.1 Transition Matrices 10
2.2.2 Secondary Structure 10
2.2.3 Conservative AA sites 11
2.3 Equivariant Graph Denoising Network 12
2.4 DDIM Sampling Process 12

3 Model Pipeline for Masked Sequence Generation **13**

4 Baseline Comparison **14**
4.1 Protein Inverse Folding on Public Benchmarks 14
4.2 Quality of Generated Novel Sequences 16
4.3 Significance of Conditions 18

5 Impact of Additional Training Data **19**
5.1 Comparison on Different Models 19
5.2 Comparison on Different Number of Training Data 21

6 Supporting Material for CPDiffusion-Based Novel Protein Sequences Generation and Evaluation **23**

6.1 AlphaFold2-based *In Silico* Screening 23
6.2 Wet Lab Experimental Evaluations 23
6.3 Bioinformatics and Structural Analyses for Sequential Evaluations . . 25
6.4 Novel PfAgo Sequence Generation and Evaluation 25

7 Related Work **26**

8 Outlook of CPDiffusion **27**
8.1 Potential Applications of CPDiffusion in Nucleic Acid-based Diagnostics and Protein Library Expansion 27
8.2 Scalability of CPDiffusion in Protein Generation 28
8.3 Broader Impact of CPDiffusion in Gene Editing and Protein Design . 29

List of Supplementary Figures

S1 Recovery rates of buried and surface residues. 16
S2 t-SNE visualization of generated 100 samples for 3FKF backbone. The recovery rate is thresholded at 50%. 17
S3 Recovery rates influenced by various modules. The percentage improvement of including each module is reported in the figure. 18
S4 Confusion matrix of the three models on generated KmAgo sequences. 20
S5 AA Distribution of the PIWI catalytic motif in generated KmAgo sequences with 0 (row 1) or all (row 2) additional pAgo dataset. . . . 21
S6 AA Distribution of the PIWI catalytic motif in generated KmAgo sequences by CPDiffusion trained with different numbers of pAgos. . . 22
S7 Conserved residues distribution in generated KmAgo sequences by CPDiffusion trained with different numbers of pAgos. 22
S8 Comparison of pLDDT of WT KmAgo and Km-AP1. 34
S9 Comparison of pLDDT of WT KmAgo and Km-AP2. 34
S10 Comparison of pLDDT of WT KmAgo and Km-AP3. 34
S11 Comparison of pLDDT of WT KmAgo and Km-AP4. 34
S12 Comparison of pLDDT of WT KmAgo and Km-AP5. 35
S13 Comparison of pLDDT of WT KmAgo and Km-AP6. 35
S14 Comparison of pLDDT of WT KmAgo and Km-AP7. 35
S15 Comparison of pLDDT of WT KmAgo and Km-AP8. 35
S16 Comparison of pLDDT of WT KmAgo and Km-AP9. 36
S17 Comparison of pLDDT of WT KmAgo and Km-AP10. 36
S18 Comparison of pLDDT of WT KmAgo and Km-AP11. 36
S19 Comparison of pLDDT of WT KmAgo and Km-AP12. 36
S20 Comparison of pLDDT of WT KmAgo and Km-AP13. 37
S21 Comparison of pLDDT of WT KmAgo and Km-AP14. 37
S22 Comparison of pLDDT of WT KmAgo and Km-AP15. 37
S23 Comparison of pLDDT of WT KmAgo and Km-AP16. 37
S24 Comparison of pLDDT of WT KmAgo and Km-AP17. 38
S25 Comparison of pLDDT of WT KmAgo and Km-AP18. 38
S26 Comparison of pLDDT of WT KmAgo and Km-AP19. 38

S27 Comparison of pLDDT of WT KmAgo and Km-AP20. 38
S28 Comparison of pLDDT of WT KmAgo and Km-AP21. 39
S29 Comparison of pLDDT of WT KmAgo and Km-AP22. 39
S30 Comparison of pLDDT of WT KmAgo and Km-AP23. 39
S31 Comparison of pLDDT of WT KmAgo and Km-AP24. 39
S32 Comparison of pLDDT of WT KmAgo and Km-AP25. 40
S33 Comparison of pLDDT of WT KmAgo and Km-AP26. 40
S34 Comparison of pLDDT of WT KmAgo and Km-AP27. 40
S35 Comparison of pLDDT of WT KmAgo and negative sample (No.0). . 41
S36 Comparison of pLDDT of WT KmAgo and negative sample (No.6). . 41
S37 Comparison of pLDDT of WT KmAgo and negative sample (No.12). . 41
S38 Comparison of pLDDT of WT KmAgo and negative sample (No.15). . 41
S39 Comparison of pLDDT of WT KmAgo and negative sample (No.17). . 42
S40 Comparison of pLDDT of WT KmAgo and negative sample (No.39). 42
S41 RMSD (right) and TM score (left) of all 100 generated KmAgo sequences. The 27 Km-APs are highlighted in red. 43
S42 Structural comparison of KmAgo and Km-APs folded by AlphaFold2. 44
S43 The electrostatic surface of MID domain and PIWI domain in KmAgo and Km-APs. 45
S44 Sequence identities of Km-APs with the WT KmAgo. 46
S45 Sequence identities of Km-APs with the most similar pAgo proteins (excluding WT KmAgo) in the training dataset. 46
S46 Sequence identities of Km-APs with the most similar protein sequence in NCBI NR (excluding WT KmAgo). 46
S47 Pairwise sequence identities of 27 Km-APs. 47
S48 The construction of plasmid of KmAgo and Km-APs linked with GFP. 57
S49 The construction of plasmid of Km-WT, Km-APs, Pf-WT, and Pf-APs. 58
S50 Guinier plots of of Km-WT and Km-APs. The lower insets show the error weighted residual difference plots for the Guinier fitting. Last panel represents the pair distribution of Km-WT and Km-APs obtained from SAXS. 59
S51 CD spectra of KmAgo (red) and 27 Km-APs (blue). 59
S52 Cleavage assay achieved by KmAgo and Km-APs expressed in vitro. Notice that the fluorescence intensity is not normalized by the concentration of proteins, this assay only reaches a qualitative conclusion on whether ssDNA is cleaved by the input protein. 60
S53 Electrophoresis mobility shift assay of the binding of Km-WT and Km-AP23 to gDNA at different concentration ratios. 60
S54 Fluorescence polarization assay of the binding of KmAgo and Km-AP23 to gDNA and tDNA. 60
S55 The cleavage activity of KmAgo and Km-APs at 37 °C is traced by fluorescence intensity as a function of time. The cleavage for Km-WT is highlighted in gray for a clearer comparison. 61

S56 The cleavage activity of KmAgo and Km-APs after incubation at 42 °C for 2 minutes. The cleavage experiment is conducted at 37 °C and is traced by fluorescence intensity as a function of time. The cleavage for Km-WT is highlighted in gray for a clearer comparison. 61
S57 The cleavage activity of KmAgo and Km-APs after incubation at 42 °C for 5 minutes. The cleavage experiment is conducted at 37 °C and is traced by fluorescence intensity as a function of time. The cleavage for Km-WT is highlighted in gray for a clearer comparison. 61
S58 The protein quantification of activity experiments of Km-APs. 62
S59 The cleavage activity experiments of Km-APs under different ratios of protein:gDNA:tDNA. 62
S60 Michaelis-Menten kinetics analysis of Km-WT, Km-AP23, and Km-AP9. 62
S61 Left panel: The cleavage activity of SeAgo, PbAgo, BlAgo, CbAgo, KmAgo, and Km-AP23 at 37 °C. Right panel: The cleavage activity of SeAgo, PbAgo, BlAgo, CbAgo, KmAgo, and Km-AP23 at 37 °C is traced by fluorescence intensity as a function of time. The blank indicates the sample contains guide DNA and target DNA. 63
S62 DNA cleavage assay on different guide and target DNA sequences from various viruses and diseases (SARS-CoV-2, KRAS, PIK3CA, and EGFR) of KmAgo and Km-AP23. 63
S63 Cleavage assay of KmAgo and Km-AP23 on different ssDNA/ssRNA targets when using different ssDNA/ssRNA guide. The blank indicates the sample contains guide and target nucleic acids. 64
S64 Left panel: complex structure of Km-WT-gRNA-tDNA. Middle panel: complex structure of Km-AP23-gRNA-tDNA. Right panel: binding free energy of gRNA/tDNA for Km-WT and Km-AP23. gRNA, tDNA, and protein are highlighted in orange, green, and blue. The binding free energy is averaged over MD trajectory. 64
S65 Catalytic motif of KmAgo, Km-AP9, Km-AP8, and Km-AP19. 66
S66 Catalytic motif of KmAgo, Km-AP22, Km-AP23 and Km-AP27. . . . 66
S67 The binding free energy of gDNA/tDNA in the catalytic pocket of Km-WT, Km-AP23, and Km-AP9 is averaged over the MD trajectory. . . 67
S68 Comparison of pLDDT of WT PfAgo and Pf-AP1. 68
S69 Comparison of pLDDT of WT PfAgo and Pf-AP2. 68
S70 Comparison of pLDDT of WT PfAgo and Pf-AP3. 68
S71 Comparison of pLDDT of WT PfAgo and Pf-AP4. 68
S72 Comparison of pLDDT of WT PfAgo and Pf-AP5. 69
S73 Comparison of pLDDT of WT PfAgo and Pf-AP6. 69
S74 Comparison of pLDDT of WT PfAgo and Pf-AP7. 69
S75 Comparison of pLDDT of WT PfAgo and Pf-AP8. 69
S76 Comparison of pLDDT of WT PfAgo and Pf-AP9. 70
S77 Comparison of pLDDT of WT PfAgo and Pf-AP10. 70
S78 Comparison of pLDDT of WT PfAgo and Pf-AP11. 70
S79 Comparison of pLDDT of WT PfAgo and Pf-AP12. 70
S80 Comparison of pLDDT of WT PfAgo and Pf-AP13. 71

S81 Comparison of pLDDT of WT PfAgo and Pf-AP14. 71
S82 Comparison of pLDDT of WT PfAgo and Pf-AP15. 71
S83 RMSD (right) and TM score (left) of 15 Pf-APs. 72
S84 Structural comparison of WT PfAgo and Pf-APs folded by AlphaFold2. 72
S85 The electrostatic surface of MID domain and PIWI domain in PfAgo and Pf-APs. 73
S86 Sequence identities of Pf-APs with the WT PfAgo. 74
S87 Sequence identities of Pf-APs with the most similar pAgo proteins (excluding WT PfAgo) in the training dataset. 74
S88 Sequence identities of Pf-APs with the most similar protein sequence in NCBI NR (excluding WT PfAgo). 74
S89 Pairwise sequence identities of 15 Pf-APs. 75
S90 The SDS-PAGE of Pf-WT and Pf-APs. 81
S91 Guinier plots of of Pf-WT and Pf-APs. The lower insets show the error weighted residual difference plots for the Guinier fitting. Last panel represents the pair distribution of Pf-WT and Pf-APs obtained from SAXS. 82
S92 The cleavage activity of KmAgo, PfAgo, and Pf-APs. The cleavage experiment of KmAgo, PfAgo, and Pf-APs is conducted at 45 °C, 45 °C and 95 °C, and 45 °C, respectively. 83
S93 The protein quantification of activity experiments of Pf-APs. 83
S94 The cleavage activity experiments of Pf-APs under different ratios of protein:gDNA:tDNA. 83
S95 Thermal unfolding curves of Pf-APs measured by DSF spectroscopy. . 84
S96 Conservative patterns of Pf-APs on the 33 conserved sites aligned from the pAgo dataset. 85
S97 AA composition of generated samples on the 4 sites of PIWI catalytic motif (DEDD) for KmAgo. Results are summarized for sequences generated by the CPDiffusion trained with (upper panel) and without (lower panel) the pAgo datasets. AAs with $>5\%$ composition are labelled for clearer presentation. 85
S98 AA composition of generated samples on the 4 sites of PIWI catalytic motif (DEDH) for PfAgo. 86
S99 Residue conservation scores for the aligned 694 WT protein, Km-APs, and Pf-APs. 86
S100 Sequence and structure similarity of WT KmAgo (left, yellow) and WT PfAgo (right, red) with other WT pAgo proteins. 86
S101 The growth temperature of associated bacterial strains of the WT pAgo proteins, grouped into short, long-A, and long-B pAgo proteins. This metric is investigated to provide an indirect evidence of the optimal temperature for enzyme activity. 87
S102 The distribution of non-mutation sites and mutation sites of Km-AP23 compared with Km-WT. 87

List of Supplementary Tables

S1 Zero-shot prediction test performance on **CATH 4.2**. 15
S2 Zero-shot prediction test performance on **TS50** and **T500**. 15
S3 Reliability of the designed novel sequences. 18
S4 Overall recovery rate on generating Ago proteins. 19
S5 Sequence specifications of Km-APs (1-3). 48
S6 Sequence specifications of Km-APs (4-6). 49
S7 Sequence specifications of Km-APs (7-9). 50
S8 Sequence specifications of Km-APs (10-12). 51
S9 Sequence specifications of Km-APs (13-15). 52
S10 Sequence specifications of Km-APs (16-18). 53
S11 Sequence specifications of Km-APs (19-21). 54
S12 Sequence specifications of Km-APs (22-24). 55
S13 Sequence specifications of Km-APs (25-27). 56
S14 SAXS data analysis of Km-WT and Km-APs. 57
S15 Michaelis-Menten analysis of Km-WT, Km-AP23, and Km-AP9. . . . 58
S16 Sequences of guide and target DNAs from different viruses and diseases. 65
S17 Additional Sequences of guide and target DNA/RNAs from nucleic acid preference analysis. 65
S18 The number of hydrogen bonds and salt bridges around Km-WT and Km-APs catalytic motif DEDD. 65
S19 Sequence specifications of Pf-APs (1-3). 76
S20 Sequence specifications of Pf-APs (4-6). 77
S21 Sequence specifications of Pf-APs (7-9). 78
S22 Sequence specifications of Pf-APs (10-12). 79
S23 Sequence specifications of Pf-APs (13-15). 80
S24 SAXS data analysis of Pf-WT and Pf-APs. 81

1 Protein Graph Representation

The protein's geometry provides insights into higher-level structures and topological relationships, which hold paramount importance in dictating protein functionality. To elucidate the physiochemical and geometric attributes of a given protein, we construct a protein graph denoted as $\mathcal{G} = (\boldsymbol{X}, \boldsymbol{E})$. This graph encapsulates essential information, with nodes corresponding to amino acids (AAs) represented by $\boldsymbol{X} \in \mathbb{R}^{35}$ node attributes. These attributes consist of a 20-dimensional AA type encoder, 12-dimensional AA properties, and 3-dimensional AA positions. The construction of undirected edge connections follows a k-nearest neighbor (kNN)-graph with a defined cutoff. In essence, each node is linked to up to k other nodes within the graph, where the link is established based on the smallest Euclidean distance relative to other nodes, provided this distance remains under a specified cutoff (*e.g.*, 30Å). Edge attributes facilitate the relationship of connected node pairs. Specifically, if nodes i and j are connected, their relationship is characterized by $\boldsymbol{E}_{ij} = \boldsymbol{E}_{ji} \in \mathbb{R}^{93}$.

The AA types are represented as one-hot encodings, resulting in 20 binary values denoted as $\boldsymbol{X}^{\text{aa}}$. Building upon this foundation, the attributes of AAs and their local environments are captured by $\boldsymbol{X}^{\text{prop}}$. This encompasses various descriptors including solvent-accessible surface area (SASA), normalized surface-aware node features, dihedral angles of backbone atoms, and 3D positions of the amino acid by the location of its C-α atom. SASA offers insights into an AA's exposure to solvent within a protein, measured through a scalar value. This metric serves as a crucial indicator of active sites within proteins, aiding in the determination of whether a residue is positioned on the protein's surface. SASA is standardized based on AA-wise mean and standard deviation associated with the attribute. Surface-aware features [1] of an AA is non-linear projections to the weighted average distance of the central AA to its one-hop neighbors $i' \in \mathcal{N}_i$, *i.e.*,

$$\rho(\mathbf{x}_i; \lambda) = \frac{\left\| \sum_{i' \in \mathcal{N}_i} w_{i,i',\lambda} \left(\boldsymbol{X}^{\text{pos},i} - \boldsymbol{X}^{\text{pos},i'} \right) \right\|}{\sum_{i' \in \mathcal{N}_i} w_{i,i',\lambda} \left\| \boldsymbol{X}^{\text{pos},i} - \boldsymbol{X}^{\text{pos},i'} \right\|},$$

where the weights are defined by

$$w_{i,i',\lambda} = \frac{\exp\left(- \left\| \boldsymbol{X}_{\text{pos},i} - \boldsymbol{X}_{\text{pos},i'} \right\|^2 / \lambda \right)}{\sum_{i' \in \mathcal{N}_i} \exp\left(- \left\| \boldsymbol{X}_{\text{pos},i} - \boldsymbol{X}_{\text{pos},i'} \right\|^2 / \lambda \right)},$$

where $\lambda \in 1, 2, 5, 10, 30$, and $\boldsymbol{X}^{\text{pos},i} \in \mathbb{R}^3$ represents the 3D coordinates of the ith residue, based on the position of the α-carbon. Additionally, the conformation of each AA along the protein chain is defined using the trigonometric values of dihedral angles $\sin, \cos \circ \phi_i, \psi_i, \omega_i$.

Edge attributes $\boldsymbol{E} \in \mathbb{R}^{93}$, on the other hand, include kernel-based distances, relative spatial positions, and relative sequential distances for pairwise distance characterization. For two connected residues i and j, their kernel-based distance is projected using Gaussian radial basis functions (RBF) expressed as $\exp\left\{ \frac{\|\boldsymbol{x}_j - \boldsymbol{x}_i\|^2}{2\sigma_r^2} \right\}$ with

$r = 1, 2, \ldots, R$. A total of 15 distinct distance-based features are formed, employing $\sigma_r = \{1.5^k \mid k = 0, 1, 2, \ldots, 14\}$. Further, local frames [1] are created from the positions of the corresponding residues' heavy atoms, serving to define 12 relative positions. These positions capture nuanced interactions between AAs and reflect how the two residues interplay in terms of rigidity. Lastly, the sequential relationship of residues is encoded through 66 binary features, which rely on the relative position $d_{i,j} = |s_i - s_j|$, where s_i and s_j denote the absolute positions of the two nodes within the AA chain [2]. Additionally, a binary contact signal [3] is introduced to signify whether two residues come into contact in space, defined by the condition $|C\alpha_i - C\alpha_j| < 8$.

2 Conditional Protein Denoising Diffusion Method

This section presents a comprehensive account of our CPDiffusion for protein sequences generation. In the characterization of the input protein's geometric and topological attributes, a graph denoted as $\mathcal{G} = \boldsymbol{X}, \boldsymbol{E}$ is established, incorporating node features denoted as $\boldsymbol{X}$ and edge features denoted as $\boldsymbol{E}$. Specifically, the node features encompass AA position, AA type, spatial properties, and biochemical properties, which can be denoted as $\boldsymbol{X} = [\boldsymbol{X}^{\text{pos}}, \boldsymbol{X}^{\text{aa}}, \boldsymbol{X}^{\text{prop}}]$. The diffusion process is defined on the AA features $\boldsymbol{X}^{\text{aa}}$, and the denoising procedure incorporates the inherent graph structure that can be adeptly encoded by *equivariant neural networks* [4]. It is noteworthy that at the denoising stage, we integrate protein-specific conditions, including an *AA substitution scoring matrix*, protein *secondary structure*, and *conserved AA sites* for graph inpainting [5].

2.1 Diffusion Process and Generative Denoising Process

Given a protein consisting of n AAs and a corresponding sequence of spatial coordinates denoted as $\boldsymbol{X}^{\text{pos}} = \{\boldsymbol{x}_1^{\text{pos}}, \ldots, \boldsymbol{x}_i^{\text{pos}}, \ldots, \boldsymbol{x}_n^{\text{pos}}\}$, wherein these coordinates are defined by the backbone Cα atoms within the protein's structure, the objective is to predict the native sequence of AAs $\boldsymbol{X}^{\text{aa}} = \{\boldsymbol{x}_1^{\text{aa}}, \ldots, \boldsymbol{x}_i^{\text{aa}}, \ldots, \boldsymbol{x}_n^{\text{aa}}\}$. This prediction encompasses the density of interactions between the targeted AA and other AAs throughout the protein chain. The training of CPDiffusion revolves around minimizing the negative log-likelihood associated with the generated AA sequence in comparison to the native WT sequence. Sequences can be generated either through sampling or by identifying sequences that maximize the conditional probability $p(\boldsymbol{X}^{\text{aa}}|\boldsymbol{X}^{\text{pos}})$ given the desired protein structure $\boldsymbol{X}^{\text{pos}}$.

2.1.1 Diffusion Process

In order to capture the distribution of AA types, a noise injection process is independently applied to each AA node within the input protein structure. For any given node, the transition probabilities are defined by the matrix $\boldsymbol{Q}_t$ that $[\boldsymbol{Q}_t]_{ij} = q(\boldsymbol{x}_t = j \mid \boldsymbol{x}_{t-1} = i)$. To establish the transitional kernel within the diffusion process, we utilize a predefined transition matrix (such as BLOSUM62 [6]) on a one-hot encoded categorical feature $\boldsymbol{X}_t^{\text{aa}}$. This allows us to define the transitional kernel as follows:

$$q\left(\boldsymbol{X}_t^{\text{aa}} \mid \boldsymbol{X}_{t-1}^{\text{aa}}\right) = \boldsymbol{X}_{t-1}^{\text{aa}} \boldsymbol{Q}_t \quad \text{and} \quad q\left(\boldsymbol{X}_t^{\text{aa}} \mid \boldsymbol{X}^{\text{aa}}\right) = \boldsymbol{X}^{\text{aa}} \bar{\boldsymbol{Q}}_t.$$

Here, $\bar{\boldsymbol{Q}}t = \boldsymbol{Q}_1 \ldots \boldsymbol{Q}t$ represents the cumulative transition probability matrix up to step t. The posterior distribution can then be calculated in closed form as

$$q\left(\boldsymbol{X}_{t-1}^{\mathrm{aa}} \mid \boldsymbol{X}_t^{\mathrm{aa}}, \boldsymbol{X}^{\mathrm{aa}}\right) \propto \boldsymbol{X}_t^{\mathrm{aa}} \boldsymbol{Q}_t^\top \odot \boldsymbol{X}^{\mathrm{aa}} \bar{\boldsymbol{Q}}_{t-1}$$

using Bayes' rule. This results in the generative probability being jointly influenced by the transition kernel, the model's output at time t, and the current state of the process $\boldsymbol{X}_t^{\mathrm{aa}}$.

It's worth noting that the generation algorithm necessitates the prior distribution $p(\boldsymbol{X}_T^{\mathrm{aa}})$ to be independent of the initial observation $\boldsymbol{X}_0^{\mathrm{aa}}$. To achieve this, the construction of the transition matrix requires the application of a noise schedule. The most commonly employed method is the uniform transition, which can be parameterized as $\boldsymbol{Q}_t = \alpha_t \boldsymbol{I} + (1-\alpha_t)\mathbf{1}_d \mathbf{1}_d^\top / d$. Here, $\boldsymbol{I}^\top$ represents the transpose of the identity matrix $\boldsymbol{I}$, d denotes the number of AA types (*i.e.*, $d = 20$), and $\mathbf{1}_d$ signifies the one vector with dimension d. As t approaches infinity, α undergoes a progressive decay until it reaches 0. Consequently, the distribution $q(\boldsymbol{X}_T^{\mathrm{aa}})$ asymptotically converges to a uniform distribution, rendering it independent of the initial state $\boldsymbol{X}^{\mathrm{aa}}$.

2.1.2 Training Denoising Networks

The denoising neural network, denoted as f_θ and parameterized by θ, constitutes the second component of the diffusion model. This network accepts a noisy input $\mathcal{G}_t = (\boldsymbol{X}_t, \mathbf{E})$ with $\boldsymbol{X}_t$ be the concatenation of the noisy AA types $\boldsymbol{X}_t^{\mathrm{aa}}$ and other AA properties $\boldsymbol{X}^{\mathrm{prop}}$. It aims to predict the clean type of AA $\boldsymbol{X}^{\mathrm{aa}}$, which allows us to model the underlying sequence diversity in the protein structure while maintaining their inherent structural constraints. The training procedure for f_θ involves optimizing the cross-entropy loss, denoted as L_{CE}, which quantifies the disparity between the predicted probabilities $\hat{p}(\boldsymbol{X}^{\mathrm{aa}})$ for the AA type associated with each node. A complete process is presented in Algorithm 1.

2.1.3 Parameterized Generative Process

A new AA sequence is generated through the reverse diffusion iterations on each node $\boldsymbol{x}$. The generative probability distribution $p_\theta(\boldsymbol{x}_{t-1}|\boldsymbol{x}_t)$ is approximated based on the projected probability $\hat{p}(\boldsymbol{x}^{\mathrm{aa}}|\boldsymbol{x}_t)$ by the neural networks. We marginalize over the network predictions to compute for generative distribution at each iteration:

$$p_\theta\left(\boldsymbol{x}_{t-1} \mid \boldsymbol{x}_t\right) \propto \sum_{\hat{\boldsymbol{x}}^{\mathrm{aa}}} q(\boldsymbol{x}_{t-1}|\boldsymbol{x}_t, \boldsymbol{x}^{\mathrm{aa}}) \hat{p}_\theta(\boldsymbol{x}^{\mathrm{aa}}|\boldsymbol{x}_t), \tag{1}$$

where the posterior

$$q\left(\boldsymbol{x}_{t-1} \mid \boldsymbol{x}_t, \boldsymbol{x}^{\mathrm{aa}}\right) = \mathrm{Cat}\left(\boldsymbol{x}_{t-1} \middle| \frac{\boldsymbol{x}_t Q_t^\top \odot \boldsymbol{x}^{\mathrm{aa}} \bar{Q}_{t-1}}{\boldsymbol{x}^{\mathrm{aa}} \bar{Q}_t \boldsymbol{x}_t^\top}\right) \tag{2}$$

an be calculated based on the transition matrix, the state of the node feature at step t, and the AA type denoted as $\boldsymbol{x}^{\mathrm{aa}}$. Here, $\boldsymbol{x}^{\mathrm{aa}}$ signifies the sample obtained from the denoising network prediction $\hat{p}(\boldsymbol{x}^{\mathrm{aa}})$.

2.2 Protein-Specific Conditions for Model Training

2.2.1 Transition Matrices

The transition matrix serves as a fundamental guide within a discrete diffusion model, facilitating seamless transitions between various states by furnishing the probabilities of transitioning from the current time step to the subsequent one. Given its function in mapping one AA type to another, this matrix plays a pivotal role in both the diffusion and generative processes. During the diffusion phase, the transition matrix undergoes iterative application to observed data, which evolves over time due to inherent noise. With the progression of diffusion time, the probability associated with the original AA type gradually diminishes, ultimately converging towards a uniform distribution encompassing all AA types. In the generative stage, the conditional probability $p(\boldsymbol{x}_{t-1}|\boldsymbol{x}_t)$ finds its determination through both the predictive capabilities of the model and the inherent attributes of the transition matrix $\boldsymbol{Q}$, as outlined in equation 1.

Given the biological context of AA substitutions, the transition probabilities between distinct AAs do not uniformly span the spectrum, thereby rendering the assignment of random directions for the generative or sampling process impractical. As an alternative, the diffusion process could be designed to reflect evolutionary pressures by leveraging substitution scoring matrices that preserve protein functionality, structure, or stability across WT protein families. Formally, an *AA substitution scoring matrix* quantifies the rates at which various AAs within proteins are substituted by alternative AAs over time [7]. In this study, we adopt the Blocks Substitution Matrix (BLOSUM) [6], which discerns conserved regions within proteins presumed to hold greater functional relevance. Rooted in empirical observations of protein evolution, the BLOSUM matrix provides an estimate of the probabilities governing transitions between diverse AAs. Consequently, we incorporate BLOSUM within both the diffusion and generative stages. Initially, the matrix is subjected to normalization using the SoftMax function. Subsequently, we apply the normalized matrix $\boldsymbol{B}$ with varying probability temperatures T to effectively control the noise scale intrinsic to the diffusion process. Consequently, the transition matrix at time t is formulated as $\boldsymbol{Q}_t = \boldsymbol{B}^T$. By employing this matrix to refine the transition probabilities, the generative space available for sampling is effectively constrained, thereby the model's predictions converge toward a meaningful subspace.

2.2.2 Secondary Structure

Protein secondary structure pertains to the local spatial arrangement of AA residues within a protein chain. The two most common types of protein secondary structure are alpha helices and beta sheets, which are stabilized by hydrogen bonds between backbone atoms. Serving as a critical intermediary, the protein secondary structure bridges the divide between AA sequences and the overall 3D conformation of the protein. In this study, we subdivide eight distinct secondary structure types as conditions into

AA nodes during the sampling process. This calculated approach effectively confines the scope of exploration within the realm of potential AA sequences. To be specific, we utilize the DSSP (Define Secondary Structure of Proteins) methodology to forecast the secondary structures of individual AAs, subsequently representing these structures through one-hot encoding. This one-hot encoding is adopted as input for our neural network, which in turn employs it to denoise the AA conditioned on it.

The imposition of motif conditions such as alpha helices and beta sheets on the search for AA sequences not only facilitates more focused exploration through a significant reduction in the sampling space of potential sequences, but also imparts biological implications for the generated protein sequence. By conditioning the sampling process of AA types on their corresponding secondary structure types, we steer the ensuing protein sequence towards not only achieving a favorable 3D structure characterized by viable thermostability but also attaining the capacity to perform its designated function effectively.

2.2.3 Conservative AA sites

Conserved AAs serve as fundamental building blocks underpinning protein functionality. They have evolved to fulfill specific roles that are pivotal for the protein's overall structure, stability, interactions, and function. To retain these key AAs during the process of generating novel AA sequences, we adopt a reverse diffusion approach within an accelerated version of the unconditional *Denoising Diffusion Probabilistic Model* (DDPM) for inpainting. This concept of inpainting finds its analogy in image processing, where missing pixels in an image are predicted based on a mask region. In our context, the unfixed AAs in protein sequence generation play the role of these missing elements to be determined by the algorithm.

Consider a trained unconditional denoising diffusion probabilistic model as defined in Equation (1). Here, we denote the true AA sequence as $\boldsymbol{x}$ and the mask as $\mathcal{M}$, which designates regions with unknown elements to guide the generation process. For any given site i, $\mathcal{M}_i = 1$ if it is a conserved site, and $\mathcal{M}_i = 0$ if it is adaptable for generation. Consequently, the conserved AA sites are obtained through the operation $\mathcal{M} \odot \boldsymbol{x}$, while the adaptable amino acid sites are obtained through $(1-\mathcal{M}) \odot \boldsymbol{x}$. Drawing inspiration from [5], we modify the known regions $\mathcal{M} \odot \boldsymbol{x}$ from the intermediary forward step $\boldsymbol{x}_t$ at a given time instance t using Equation (2). Consequently, at a singular reverse step, we formulate the following equation:

$$\boldsymbol{x}_{t-1} = \mathcal{M} \odot \boldsymbol{x}_{t-1}^{\text{known}} + (1 - \mathcal{M}) \odot \boldsymbol{x}_{t-1}^{\text{known}}, \tag{3}$$

where $\boldsymbol{x}_{t-1}^{\text{known}} \sim q(\boldsymbol{x}_t | \boldsymbol{x}^{\text{aa}})$ signifies the known AA types at time $t-1$, derived from the input sequence $\mathcal{M} \odot \boldsymbol{x}$. Meanwhile, $\boldsymbol{x}_{t-1}^{\text{unknown}} \sim p_\theta(\boldsymbol{x}_{t-1} | \boldsymbol{x}_t)$ embodies sampling from the model during the reverse diffusion phase, grounded in the previous state. Ultimately, the outcomes merge into the conclusive sample $\boldsymbol{x}_{t-1}$ at time $t-1$ with the mask in Equation (3).

2.3 Equivariant Graph Denoising Network

Biomolecules, including proteins and chemical compounds, inherently possess complex 3-dimensional structures. Ensuring that the model consistently predicts the same binding complex, regardless of the positions and orientations of input proteins, is pivotal for encoding a robust and expressive hidden representation. This crucial property is achieved through the concept of rotation equivariance in deep neural networks, such as the equivariant graph neural network (EGNN) [4].

We enhance the SE(3)-equivariant neural layers of EGNN to enable the updating of representations for both nodes and edges, while preserving SO(3) rotation equivariance and E(3) translation invariance. At the lth layer, an Equivariant Graph Convolution (EGC) takes as input a collection of n hidden node embeddings, denoted as $\boldsymbol{H}^{(l)} = \left\{\boldsymbol{h}_1^{(l)}, \ldots, \boldsymbol{h}_n^{(l)}\right\}$, which capture information about AA type and geometry properties. Additionally, edge embeddings $\boldsymbol{m}_{ij}^{(l)}$ corresponding to connected nodes i and j, and node coordinates $\boldsymbol{X}^{\text{pos}} = \{\boldsymbol{x}_1^{\text{pos}}, \ldots, \boldsymbol{x}_n^{\text{pos}}\}$ are provided. The goal of our modified EGC layer is to update hidden node representations $\boldsymbol{H}^{(l+1)}$ and edge representations $\boldsymbol{M}^{(l+1)}$. In concise terms, the update process is denoted as $\boldsymbol{H}^{(l+1)}, \boldsymbol{M}^{(l+1)} = \text{EGC}\left[\boldsymbol{H}^{(l)}, \boldsymbol{X}^{\text{pos}}, \boldsymbol{M}^{(l)}\right]$. To achieve this, an EGC layer is defined by the following equations:

$$
\begin{aligned}
\boldsymbol{m}_{ij}^{(l+1)} &= \phi_e\left(\mathbf{h}_i^{(l)}, \mathbf{h}_j^{(l)}, \left\|\mathbf{x}_i^{(l)} - \mathbf{x}_j^{(l)}\right\|^2, \boldsymbol{m}_{ij}^{(l)}\right) \\
\boldsymbol{x}_i^{(l+1)} &= \mathbf{x}_i^{(l)} + \frac{1}{n}\sum_{j\neq i}\left(\mathbf{x}_i^{(l)} - \mathbf{x}_j^{(l)}\right)\phi_x\left(\mathbf{m}_{ij}^{(l+1)}\right) \\
\boldsymbol{h}_i^{(l+1)} &= \phi_h\big(\mathbf{h}_i^{(l)}, \sum_{j\neq i}\mathbf{m}_{ij}^{(l+1)}\big),
\end{aligned}
\tag{4}
$$

where ϕ_e and ϕ_h denote the edge and node propagation operations, respectively. The operation ϕ_x projects the vector edge embedding $\boldsymbol{m}_{ij}$ into a scalar. This modified EGC layer retains its equivariance to rotations and translations with respect to the set of 3D node coordinates $\boldsymbol{X}^{\text{pos}}$, and simultaneously maintains invariance to permutations within the nodes set, a characteristic common to other GNNs.

2.4 DDIM Sampling Process

One notable limitation in DDPM is the trade-off between the speed reduction and the performance enhancement during the generation process. In continuous diffusion generative models, the *Deterministic Denoising Implicit Model* (DDIM) [8] is frequently employed to address this concern. DDIM adopts a non-Markovian forward diffusion process and conditions on the initial input without referring to the intermediate steps. The reverse generative process can be determined by the initial prior sample by assigning the stepwise noise variance to 0.

We thus extend this sampling technique to our discrete diffusion and define the multi-step generative process as

$$p_\theta\left(\boldsymbol{x}_{t-k} \mid \boldsymbol{x}_t\right) \propto \sum_{\hat{\boldsymbol{x}}^{\text{aa}}} q(\boldsymbol{x}_{t-k}|\boldsymbol{x}_t, \boldsymbol{x}^{\text{aa}})\hat{p}^T(\boldsymbol{x}^{\text{aa}}|\boldsymbol{x}_t). \tag{5}$$

Here, the closed-form of the generative probability $p_\theta(\boldsymbol{x}_{t-1}|\boldsymbol{x}_t)$ is approximated by $\hat{\boldsymbol{x}}^{\text{aa}}$, the network estimation for the true $\boldsymbol{x}^{\text{aa}}$, and the posterior distribution $p(\boldsymbol{x}_{t-1}|\boldsymbol{x}_t, \boldsymbol{x}^{\text{aa}})$. The generative model is made deterministic the sampling temperature T of $p(\boldsymbol{x}^{\text{aa}}|\boldsymbol{x}_t)$, where T governs the level of sampling stochasticity.

3 Model Pipeline for Masked Sequence Generation

We now present an overview of the training and inference methodologies implemented in the generative module of CPDiffusion. The pseudo-codes are respectively provided in Algorithm 1-2.

In Algorithm 1, training begins with a random sampling of a time scale t from a uniform distribution between 1 and T. Subsequently, we calculate the noise posterior $q(\boldsymbol{X}_t|\boldsymbol{X}^{\text{aa}})$ and sample the perturbed $\boldsymbol{X}_t$ from it. Next, an equivariant graph neural network is trained to predict $\boldsymbol{X}^{\text{aa}}$ with the noisy $\boldsymbol{X}_t$ at time t, along with other determined prior from the protein backbone, such as node interaction ($\boldsymbol{E}$) and secondary structure (ss). The trainable weights are back-propagated with cross-entropy between the predicted and original AA types.

Algorithm 1 Training

1: **Input**: graph $\mathcal{G} = \{\boldsymbol{X}, \boldsymbol{E}\}$
2: Sample $t \sim \mathcal{U}(1, T)$
3: Compute $q(\boldsymbol{X}_t|\boldsymbol{X}^{\text{aa}}) = \boldsymbol{X}^{\text{aa}}\bar{Q}_t$
4: Sample $\boldsymbol{X}_t \sim q(\boldsymbol{X}_t|\boldsymbol{X}^{\text{aa}})$
5: Predict $\hat{p}(\boldsymbol{X}^{\text{aa}}) = f_\theta(\boldsymbol{X}_t, \boldsymbol{E}, t, ss)$
6: Compute the cross-entropy loss $L_{\text{CE}}(\hat{p}(\boldsymbol{X}^{\text{aa}}), \boldsymbol{X})$
7: Update $f_\theta(\cdot)$ with the gradient

Algorithm 2 presents the workflow for sequence generation conditioned on fixed AAs (*e.g.*, conservative sites). With the trained neural network $f_\theta(\cdot)$, it is possible to sample AA sequences for a fixed protein backbone, starting from a uniform sampling of AA sequences $\boldsymbol{X}_T$ from 20 classes. The iterative denoising procedure is then implemented with $f_\theta(\cdot)$ for $\boldsymbol{X}^{\text{aa}}$ at every time t until ultimately approximating the original data distribution. The target is to recover the distribution $p(\boldsymbol{x}_{t-k}|\boldsymbol{x}_t)$ for sampling. The difference between DDIM and DDPM lies in step 6 of computing $p(\boldsymbol{x}_{t-k}|\boldsymbol{x}_t)$, where DDIM skips the first k steps in DDPM to achieve a close performance with faster speed. When $k = 1$, DDIM degrades to DDPM. Moreover, the masked update is implemented in step 5 when some AAs are desired to be fixed during sampling. It follows the formulation in Equation (3), which updates the prediction on fixed sites

to its assigned types. Note that this step can be skipped when the mask $\mathcal{M}$ is not provided, *i.e.*, there are no sites to fix for generation.

Algorithm 2 Masked Sampling

Input: graph $\mathcal{G} = \{\boldsymbol{X}, \boldsymbol{E}\}$; mask $\mathcal{M}$
Sample from uniformly prior $\boldsymbol{X}_T \sim p(\boldsymbol{X}_T)$
for t in $\{T, T-k, ..., 1\}$ **do**
  Predict $\hat{p}(\boldsymbol{X}^{\text{aa}} | \, \boldsymbol{X}_t) = f_\theta(\boldsymbol{X}_t, \boldsymbol{E}, t, ss)$
  Update $\hat{p}(\boldsymbol{X}^{\text{aa}} | \, \boldsymbol{X}_t) = \mathcal{M} \odot p(\boldsymbol{X}^{\text{aa}}) + (1 - \mathcal{M}) \odot \hat{p}(\boldsymbol{X}^{\text{aa}} | \, \boldsymbol{X}_t)$
  Compute $p_\theta(\boldsymbol{X}_{t-k} | \boldsymbol{X}_t) = \sum_{\hat{\boldsymbol{X}}^{\text{aa}}} q(\boldsymbol{X}_{t-k} | \boldsymbol{X}_t, \hat{\boldsymbol{X}}^{\text{aa}}) \hat{p}(\boldsymbol{X}^{\text{aa}} | \boldsymbol{X}_t)$
  Sample $\boldsymbol{X}_{t-k} \sim p_\theta(\boldsymbol{X}_{t-k} | \boldsymbol{X}_t)$
end for
Sample $\boldsymbol{X}^{\text{aa}} \sim p_\theta(\boldsymbol{X}^{\text{aa}} | \boldsymbol{X}_1)$

4 Baseline Comparison

To validate the significance of CPDiffusion, we design *in silico* evaluations with extensive experiments. In Section 4.1, we compare the ability of CPDiffusion with other structure-aware deep learning methods that have achieved SOTA performance in recovering native protein sequences. While standard evaluation metrics such as perplexity and recovery rate are often criticized for not meeting the empirical requirements of designing novel protein sequences, Section 4.2 conducts additional assessments on the local recovery, generation diversity, and foldability. Furthermore, Section 4.3 comprehensively explores the role of conditions in training CPDiffusion, including additional WT proteins in the training dataset, conservative sites, equivariant graph neural networks, and secondary structure.

4.1 Protein Inverse Folding on Public Benchmarks

Several structure-aware deep learning methods have emerged to address the inverse folding task, aiming to recover sequences for a given backbone structure. As the generative module of CPDiffusion primarily aligns with this category, this section conducts a comparative analysis with existing SOTA models on three widely used benchmarks. Notably, this experiment focuses on recovering the entire protein sequence, and therefore, no conservative sites are assigned using the mask matrix.

We commence with an overview of the three benchmark datasets, the training setups, and the two evaluation metrics used for baseline comparison.

The first and most widely-used benchmark **CATH 4.2** was collected by Ingraham *et al.* [3] from the **CATH** hierarchical classification of protein structure [9]. This benchmark comprises $\sim 20,000$ chains that are no longer than 500 AAs from a pool of non-redundant domains (with a 40% threshold). We follow the public split of the train, validation, and test sets, yielding a total of $18,024 : 608 : 1,120$ chains after additional overlapping removal steps to avoid data leakage. Evaluation is segmented

Table S1: Zero-shot prediction test performance on **CATH 4.2**.

**Model**	**Perplexity ↓**			**Recovery Rate % ↑**		
	Short	Single-chain	All	Short	Single-chain	All
StructGNN [3]	8.29	8.74	6.40	29.44	28.26	35.91
GraphTrans [3]	8.39	8.83	6.63	28.14	28.46	35.82
GVP [11]	7.23	7.84	5.36	30.60	28.95	39.47
GCA [12]	7.09	7.49	6.05	32.62	31.10	37.64
AlphaDesign [13]	7.32	7.63	6.30	34.16	32.66	41.31
ProteinMPNN [14]	6.21	6.68	4.57	36.35	34.43	49.87
PiFold [15]	6.04	6.31	4.55	39.84	38.53	51.66
CPDiffusion	**5.49**	**6.21**	**4.06**	**45.27**	**42.77**	**53.70**

Table S2: Zero-shot prediction test performance on **TS50** and **T500**.

	**TS50**		**T500**	
	Perplexity ↓	Recovery ↑	Perplexity ↓	Recovery ↑
StructGNN [3]	5.40	43.89	4.98	45.69
GraphTrans [3]	5.60	42.20	5.16	44.66
GCA [12]	5.09	47.02	4.72	47.74
GVP [11]	4.71	44.14	4.20	49.14
AlphaDesign [13]	5.25	48.36	4.93	49.23
ProteinMPNN [14]	3.93	54.43	3.53	58.08
PiFold [15]	3.86	**58.72**	3.44	60.42
CPDiffusion	**3.71**	56.32	**3.23**	**61.22**

into three groups of test proteins: **short** (proteins with fewer than 100 AAs), **single-chain** (proteins composed of a single chain in PDB), and **all** (all proteins in the test set). The other two benchmarks, **TS50** and **T500** [10], encompass distinct test datasets of 50 and 500 proteins, respectively. The number of training sets for both benchmarks contains $9,888$ structures. The overlap between **CATH** and **TS50/T500** in the test set is as small as 4 structures.

There are several hyper-parameters for training the diffusion module. For **CATH 4.2**, the total time step is set to 500, and a cosine schedule for noise addition [16] is employed for the forward diffusion. The trainable neural network $f_\theta(\cdot)$ consists of 6 EGC layers of 128 hidden neurons. The model undergoes 200 epochs of training with an Adam [17] optimizer. The batch size and learning rate are set to 64 and 0.0005, respectively, with a dropout rate of 0.1 to prevent overfitting. For **TS50** and **T500**, most settings remain the same, except for the number of hidden neurons, which is increased to 256.

The quality of recovered protein sequences is assessed using two common metrics. *Perplexity* measures the alignment of the model's predicted amino acid probabilities with the actual amino acids at each position in the sequence. Lower perplexity values indicate a better fit of the model to the data. *Recovery rate* evaluates the model's ability to reconstruct the correct amino acid sequence from inputs, *e.g.*, protein backbone. It is calculated as the proportion of amino acids in the predicted sequence that

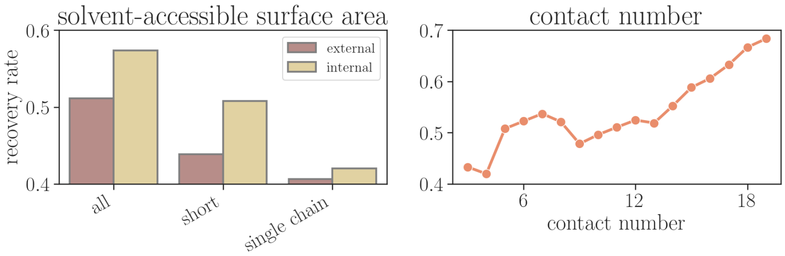

Fig. S1: Recovery rates of buried and surface residues.

matches the original sequence. A higher recovery rate signifies a better capability of the model to predict the original sequence from the structure.

Table S1 compares CPDiffusion's performance in recovering proteins in **CATH 4.2** test set. Across all three groups of test proteins, CPDiffusion achieves the lowest perplexity and the highest recovery rate compared to baseline methods. Similarly, for **TS50** and **T500** (Table S2), CPDiffusion again demonstrates top test performance over baseline methods. Notably, all baseline performance scores for the three benchmarks are retrieved from [15] following the standard training and testing split provided in the original study, which CPDiffusion followed as well.

While low perplexity and high recovery rates have become the standard benchmarks for assessing the quality of a deep learning method in generating protein sequences, the intricate relationship between protein structure and sequence challenges the validity of these two metrics. The ability to fold different sequences into a single structure [18] and the potential disruption of an entire protein due to a mutation in a crucial site (such as the catalytic site) pose significant challenges. Since it is commonly believed that amino acids (AAs) situated within a protein's interior tend to be more conserved compared to those exposed on the surface, our investigation delves into the local recovery performance of CPDiffusion based on the positions of AAs, as illustrated in Fig. S1. We follow [19] and define the AA conservation jointly by the Solvent-Accessible Surface Area (SASA) and the contact number, which denotes the count of neighboring AAs within 8 Å in 3D space. Specifically, we define SASA< 0.25 as internal AAs. For the latter contact number, an AA with a larger contact number indicates a higher chance of it locating on the surface of a protein. Our findings indicate that across all three classes of proteins in **CATH 4.2**, the buried core residues showcase a higher native sequence recovery rate, whereas the active surface AAs exhibit a lower sequence recovery rate.

4.2 Quality of Generated Novel Sequences

Structure-based novel sequence design typically aims to produce multiple AA sequences that exhibit diversity and high confidence in expressivity and foldability. In this regard, this section conducts additional comparisons on diversity and reliability

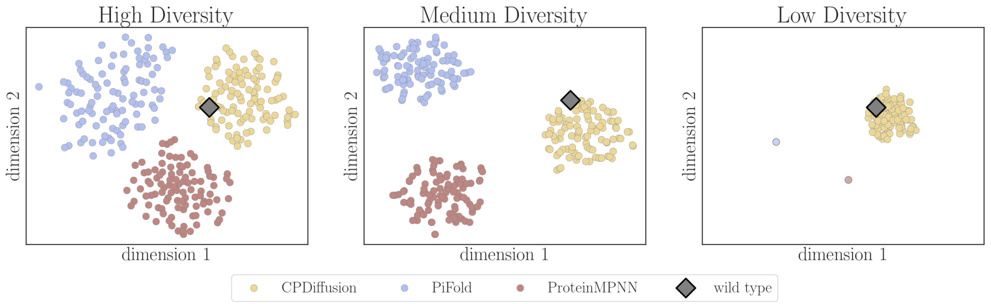

Fig. S2: t-SNE visualization of generated 100 samples for 3FKF backbone. The recovery rate is thresholded at 50%.

for CPDiffusion with two baseline models that perform the top in the previous section, *i.e.*, ProteinMPNN [1] and PiFold [2].

Diversity

In the generation of multiple novel proteins, a model's capability to output diverse sequences folding to the same backbone and performing the same function is crucial. This section investigates the diversity of CPDiffusion in comparison to the baseline methods. We sample 100 sequences for an arbitrary backbone in **CATH 4.2** test dataset (PDB ID: 3FKF) and visualize the t-SNE [20] results in Fig. S2. We adjust temperatures in $\{0.5, 0.1, 0.0001\}$ for the baseline methods and sample step in $\{1, 10, 50\}$ for CPDiffusion during inference to approximately simulate scenarios of high, medium, and low diversity. At the same level of diversity, CPDiffusion encompasses the WT sequence, while the other two methods fail to include the WT 3FKF within their sample region. Meanwhile, inferencing with a large sample step (50) allows CPDiffusion to generate diverse sequences, whereas the other two methods revert to deterministic results. This further substantiates the superiority of our model in simultaneously achieving sequence diversity and a high recovery rate.

Reliability

The reliability of the designed diverse sequences is another crucial aspect of generative models. We approach the reliability of novel sequences by their structures from three perspectives: foldability, confidence, and deviation from the native structure. Specifically, we select the first 100 backbones (arranged alphabetically by their PDB ID) from the test dataset and generate 100 sequences for each backbone for comparative analysis. The structures of these novel sequences are predicted using AlphaFold2. The results, presented in Table S3, encompass key metrics. In particular, *foldability* is defined following [21], which measures the quality of a novel sequence using the TM score between its AlphaFold2 structure and the native structure. A high TM score indicates that the predicted structure closely aligns with the native one. A design is considered successful or foldable if the TM score exceeds 0.5. The *confidence* is assessed

[1] https://github.com/dauparas/ProteinMPNN
[2] https://github.com/A4Bio/PiFold

Table S3: Reliability of the designed novel sequences.

Method	Success	TM score	avg pLDDT	avg RMSD
PiFOLD	85	0.80 ± 0.22	0.84 ± 0.15	1.67 ± 0.99
ProteinMPNN	94	0.86 ± 0.16	0.89 ± 0.10	1.36 ± 0.81
CPDiffusion	94	0.86 ± 0.17	0.86 ± 0.08	1.47 ± 0.82

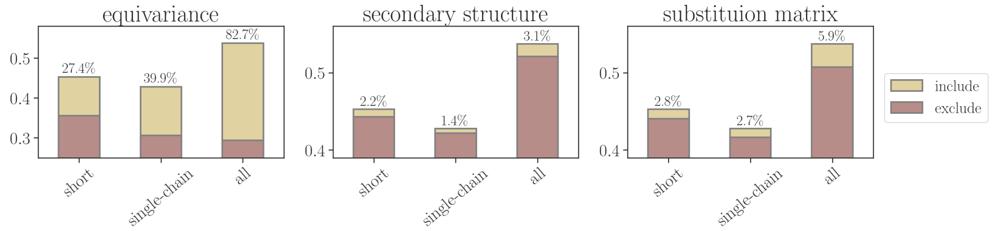

Fig. S3: Recovery rates influenced by various modules. The percentage improvement of including each module is reported in the figure.

by pLDDT from AlphaFold2, where a predicted structure is generally considered of high confidence if the value is the overall pLDDT higher than 0.8. The *structural difference* is measured by the average RMSD of aligned protein pairs between the AlphaFold2 structure and the native structure.

Overall, CPDiffusion demonstrates high pLDDT, low RMSD, and substantial foldability, signifying its capability to generate novel sequences with reliably predicted structures. It is noteworthy that certain proteins, such as 1BCT, 1BHA, and 1CYU, pose challenges for all three methods in achieving high foldability scores. These proteins have structures determined through NMR, which is an experimental technique that analyzes protein structures in a buffer solution. Due to the presence of multiple structures for a single protein in NMR studies, assigning relatively lower foldability scores by folding tools is reasonable.

4.3 Significance of Conditions

As CPDiffusion incorporates multiple conditions during training, this section delves into the impact of these conditions through ablation studies using **CATH 4.2**.

Fig. S3 presents a comparison of recovery rates between ablation models on the **CATH 4.2** test set and the complete CPDiffusion. Three modules undergo investigation, including the equivariant graph neural network (replaced by GCN [22] in ablation), the secondary structure (omitting the DSSP-based encoding during the denoising phase in ablation), and the BLOSUM62 substitution matrix (replaced by uniform perturbation in ablation). In the figure, the performance of ablation modules is highlighted in pink, and the results of complete CPDiffusion, including all modules, are represented in yellow.

All three modules contribute to the improved recovery performance of the ablation model, with the most significant gains observed in long protein sequences (those

Table S4: Overall recovery rate on generating Ago proteins.

Model	# Ago	Test-50 Recovery %	KmAgo Recovery %
ESM-IF1	0	42.90	35.08
ProteinMPNN	0	50.26	48.60
	all	75.02	65.70
CPDiffusion	0	43.55	36.64
	100	60.78	42.88
	300	72.49	47.40
	all	77.47	64.62

exceeding 100 AAs and with more than one chain in the PDB structure). Notably, the inclusion of equivariance in learning the geometry of proteins yields the most substantial enhancement. This encoding captures symmetry in the denoising neural network, enabling it to respect the arbitrary placement of protein structures in 3D space.

5 Impact of Additional Training Data

The complete CPDiffusion is trained on $\sim 20,000$ WT proteins from **CATH 4.2** plus 694 pAgos from [23]. To validate the effectiveness of including the additional pAgos in the training set, we design two experiments that investigate the effect of including (1) *additional pAgos on training* ***different models*** (Section 5.1); (2) ***different numbers*** *of pAgos on training CPDiffusion* (Section 5.2). We evaluate the model performance from three dimensions, including the overall recovery rate, the recovery of the PIWI catalytic motif, and the recovery of other conservative sites.

5.1 Comparison on Different Models

We assess the impact of including all pAgos entries in the training set by comparing the prediction performance of the models trained with and without this task-specific dataset. Two types of comparisons are established. The first evaluation involves randomly selecting 50 test pAgos, where the remaining 643 pAgos, if used for training, will be included in the training set. The second evaluation picks the WT KmAgo for testing, leaving all the remaining 693 pAgos for training. The overall recovery performance of CPDiffusion is compared with other structure-based sequence generation methods, ESM-IF1 [3] and ProteinMPNN. Notably, ESM-IF1 is pre-trained on ~ 20 million AlphaFold2 protein structures. Thus, we infer only the pre-trained version using the provided checkpoint to ensure predictions from a sufficiently trained model. For ProteinMPNN, we re-trained the entire model with the same set of training data used for training CPDiffusion, including **CATH4.2** and the pAgo dataset.

Table S4 reports the performance of models trained on different numbers of Ago proteins using the two evaluation criteria introduced above. The second column (# Ago) provides the number of Ago proteins used in training. All protein instances are picked randomly from the 643 training Ago proteins. The next two columns report the recovery rate averaged on the 50 test Ago proteins and the recovery rate of the

[3] https://github.com/facebookresearch/esm

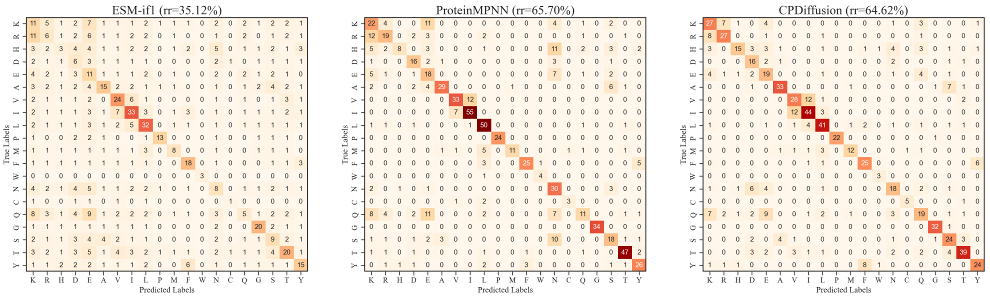

Fig. S4: Confusion matrix of the three models on generated KmAgo sequences.

KmAgo protein, respectively. Of all three models we tested here, the recovery rate of ESM-IF1 is the lowest at around 40% and 35%. While the model scale and the closed-source training program prohibit re-training ESM-IF1 with additional pAgo data, the performance gain on both PROTEINMPNN and CPDiffusion after introducing same-family proteins to the training data. This proves the importance of reshaping the generative model with specialized proteins, which rely heavily on a considerably small training scale to implement. We also show on CPDiffusion that increasing the number of pAgo family data for training enhances the overall performance on the generation task in terms of the global sequence recovery rate.

In addition to the overall recovery rate, Fig. S4 visualizes the AA-wise recovery with confusion matrices. For ESM-IF1 the pre-trained version was employed, and for PROTEINMPNN and CPDiffusion were trained on **CATH 4.2** plus all WT pAgo proteins. For all three models, 100 KmAgo sequences were generated and calculated to obtain the average scores for evaluation. The global recovery rate is reported on the title of each subplot. Comparing the confusion matrices of PROTEINMPNN and CPDiffusion reveals that both methods can generate better results than ESM-IF1, which, to some extent, due to the utilization of additional pAgo datasets during training. Although CPDiffusion does not exhibit a significant advantage on the overall recovery rate over PROTEINMPNN, its performance in recovering polar AAs is superior, a pivotal type of AA in protein-nucleic acid interactions. Additionally, CPDiffusion demonstrates a significant advantage in predicting Cysteine (C) compared to PROTEINMPNN. While C is typically present in small quantities in proteins, it plays a vital role in the solubility and stability of proteins [24]. On the other hand, PROTEINMPNN shows better recovery for Valine (V), Isoleucine (I), and Leucine (L), compared to CPDiffusion. However, these three AAs have similar properties and are to some extent interchangeable. This interchangeability is well reflected in the predictions made by CPDiffusion.

Next, we validate the validity of the generated KmAgos. Specifically, we generate 100 sequences respectively by PROTEINMPNN and CPDiffusion to count the occurrence of AAs on the PIWI catalytic motif designed by both models. The results are visualized in Fig. S5. The four sites are pivotal to the enzymatic activity of KmAgo, and mis-generating any of them would result in catalyst deactivation. As shown in the upper row, all three models achieve poor generation performance when the training

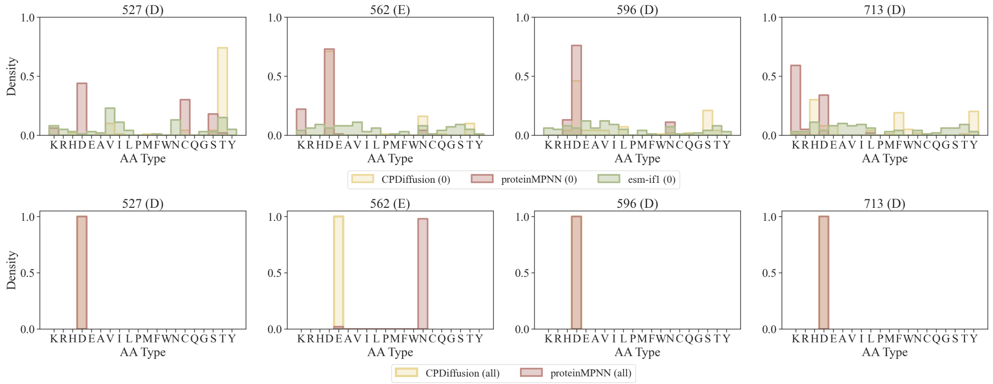

Fig. S5: AA Distribution of the PIWI catalytic motif in generated KmAgo sequences with 0 (row 1) or all (row 2) additional pAgo dataset.

dataset does not include pAgo proteins, indicating the importance of a valid model to allow fine-tuning or re-training with enhanced datasets for a particular protein. Furthermore, in the lower row, we compare the generation performance on CPDiffusion and ProteinMPNN. Here ESM-IF1 is excluded due to the significant resources required to retrain the model. While both methods manage to capture the conservation on 527, 596, and 713, ProteinMPNN recovers the 562nd site incorrectly as N (Asparagine). Consequently, all generated AP KmAgos would be dysfunctional.

5.2 Comparison on Different Number of Training Data

We next investigate the impact of including different volumes of WT pAgos in the training set. Similar to before, we train different versions of CPDiffusion with various numbers of WT pAgos that are randomly selected from the dataset (excluding WT KmAgo) and visualize the results in Fig. S6-S7 for the distribution of the PIWI catalytic motif and other conservative sites, respectively. Overall, training with additional Ago proteins is essential for the model to understand the conservation on all the 36 sites, where introducing more pAgos constantly enhances the model's ability. On the other hand, as visualized in Fig. S6, while including arbitrary numbers of pAgo in training enables the model to lock AA type to D (Aspartic Acid) at positions 596 and 713, only the complete CPDiffusion (in yellow) trained on all pAgos manages to correctly generate AAs at positions 527 and 562. Consequently, it is sufficient to train the model with a relatively small amount of pAgo datasets of 693 proteins.

It is also notable that some conservative sites in Fig. S7 do not converge to a single AA type. This result implies CPDiffusion's ability to increase the diversity of the novel sequences while maintaining the generated AAs within the group that shares similar properties. For instance, position 543 has a high chance of generating V (Valine) in addition to I (Isoleucine), both of which are hydrophobic AAs.

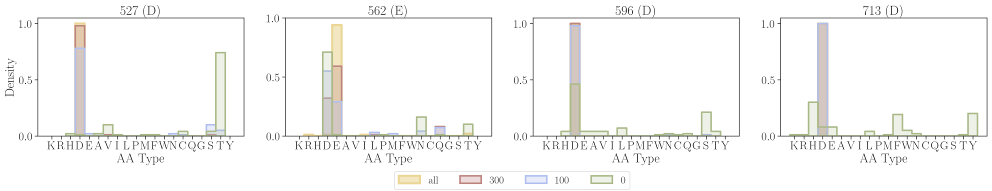

Fig. S6: AA Distribution of the PIWI catalytic motif in generated KmAgo sequences by CPDiffusion trained with different numbers of pAgos.

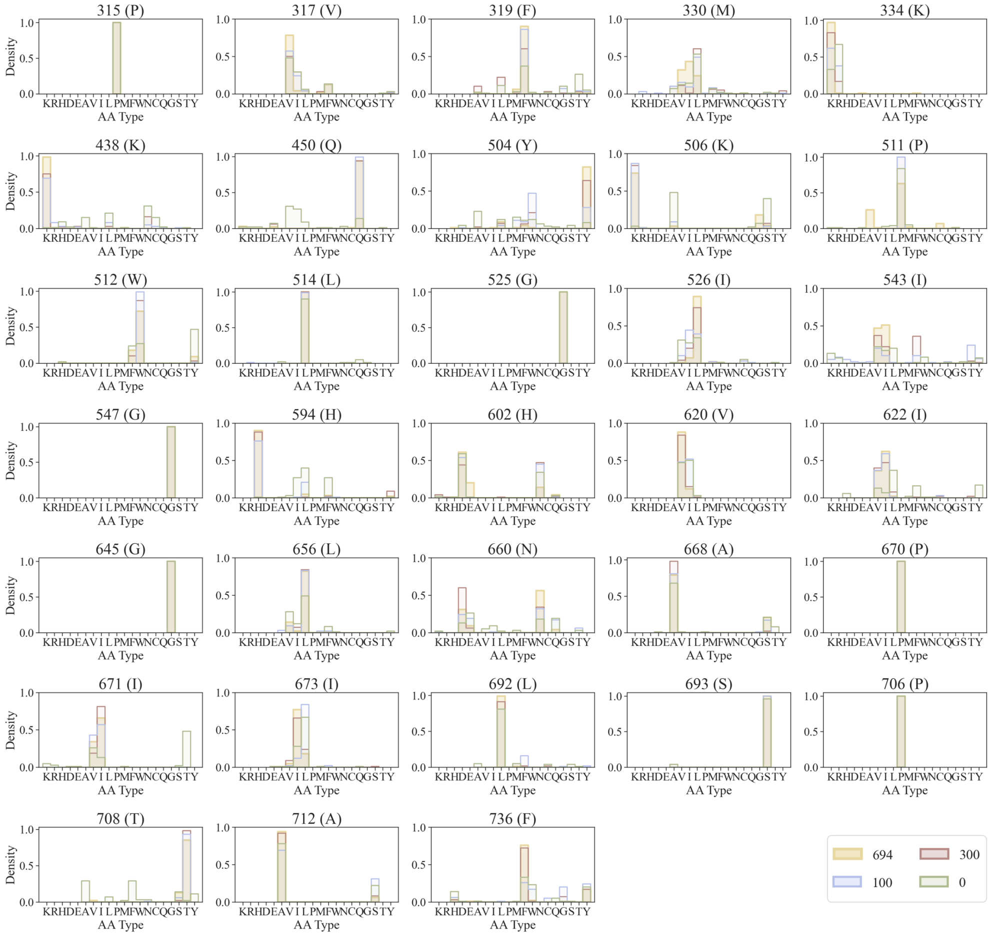

Fig. S7: Conserved residues distribution in generated KmAgo sequences by CPDiffusion trained with different numbers of pAgos.

6 Supporting Material for CPDiffusion-Based Novel Protein Sequences Generation and Evaluation

This section utilizes KmAgo and PfAgo as examples to summarize the screening and experimental validation process of the novel protein sequences generated by CPDiffusion. Specifically, Section 6.1 outlines supporting information for the structural evaluation based on AlphaFold2 for all 100 sequences generated using the WT KmAgo backbones, with additional details provided for the 27 selected APs. This demonstration aims to illustrate the filtering process introduced in Section 2 of the maintext. Section 6.2 then provides a summary of the wet-lab experiments on KmAgo, detailing the validation methods and experimental results. Subsequently, Section 6.3 supplements additional bioinformatics analyses and structural analyses for the 27 APs of KmAgo. As a supplementary case, Section 6.4 briefly introduces the filtering and experimental validation of APs for PfAgo. Here, the AP based on KmAgo and PfAgo is labeled as Km-AP and Pf-AP, respectively.

6.1 AlphaFold2-based *In Silico* Screening

The residue-wise pLDDT (by AlphaFold2) and RMSD were used for pre-screening the generated sequences for KmAgo. In Fig. S8-S34, we visualize line plots for the selected 27 Km-APs and compare them to those of WT KmAgo. For comparison, Fig. S35-S40 display pLDDT plots of six randomly selected negative entries. The pLDDT line of these negative samples, in comparison to WT KmAgo's, all exhibit notable local gaps. Fig. S41 presents details on the RMSD and TM scores of all 100 generated sequences, with the 27 Km-APs highlighted in red. All 27 Km-APs exhibit considerably small RMSD values ($\leq$ 2.5 Å) and large TM scores ($>$ 0.9), indicating that all generated sequences are reasonably believed to fold into a structure similar to WT KmAgo [25, 26]. Consequently, we conclude that all generated sequences demonstrate satisfying reliability, with the chosen 27 Km-APs achieving a slightly superior state. We additionally check the 3D structure and electrostatic surface of the MID domain and PIWI domain, both of which are consistent for WT KmAgo and Km-APs, as shown in Figs. S42-Fig. S43.

6.2 Wet Lab Experimental Evaluations

Expression, Purification, Activity, and Thermostability of Km-APs

The sequences of the 27 selected sequences for wet experiment evaluation are listed in Tables S5-S13. These proteins are expressed in *E. coli*. The construction of associated plasmids, both with and without GFP, is illustrated in Figs. S48 and S49, respectively. The SAXS results demonstrate that Km-APs stay as monomer in buffer (Fig. S50 and Table S14). Upon successful expression of all 27 Km-APs, their secondary structures and overall packings are examined using CD spectra (Fig. S51) and SAXS (Fig. S50). Both results of Km-APs are compared to those of WT KmAgo, suggesting the correct folding of the Km-APs.

Subsequently, we investigate the cleavage activity of the Km-APs on nucleic acids by first assessing the ssDNA cleavage activity at 37 °C with two evaluation assays. *In*

vitro results are displayed in Fig. S52, and normalized quantitative performance is presented in Figs. S55 and S58. We also conduct the cleavage activity experiments of the Km-APs on ssDNA with different ratio of protein:gDNA:tDNA to demonstrate that the activity of Km-AP21, Km-AP22, Km-AP23, and Km-AP25 is indeed enhanced Fig. S59.

In Fig. S54, the fluorescence polarization assay on KmAgo and Km-AP23 (the best-performed Km-AP with 8.6 times DNA cleavage activity enhancement over WT KmAgo) is conducted to study the binding affinity to gDNA and tDNA. The results reveal that the dissociation constant (Kd) of Km-AP23 is lower than that of KmAgo, demonstrating increased binding affinity of Km-AP23 to gDNA and tDNA. Furthermore, EMSA (Fig. S53) and Michaelis-Menten kinetics analysis (Fig. S60 and Table S15) demonstrate that Km-AP23 has a high affinity for gDNA and high cleavage efficiency with tDNA, respectively.

Furthermore, we assess the thermostability of the Km-APs by incubating them at 42 °C for 2 minutes (Fig. S56) and 5 minutes (Fig. S57), followed by assays examining ssDNA cleavage activity. The results indicated that 10 out of 27 Km-APs exhibited greater thermostability than the WT KmAgo.

Cleavage Activity Enhancement of Km-AP23 over Other Mesophilic Ago Proteins and on Different Guide/Targets

To confirm the enhancement of Km-AP23 on DNA cleavage activity at 37 °C, we design comprehensive evaluations with various baseline WT pAgo proteins and guides/targets. Firstly, we compare the performance of Km-AP23 with four other WT mesophilic pAgo proteins, including *Brevibacillus laterosporus* Argonaute protein (BlAgo), *Paenibacillus borealis* Argonaute protein (PbAgo), *Clostridium butyricum* Argonaute protein (CbAgo), and *Synechococcus elongatus* Argonaute protein (SeAgo). The results are reported in Fig. S61, where Km-AP23 exhibits significantly higher cleavage activity than other mesophilic pAgo proteins. Next, we explore the cleavage assay of Km-AP23 on different guide and target ssDNA from four distinct viruses and diseases: SARS-CoV-2 (the one used for the majority of experiments), KRAS, PIK3CA, and EGFR. The corresponding guide and target DNA sequences are listed in Table S16. Fig. S62 compares the cleavage activity of Km-AP23 and WT KmAgo for these four different ssDNA, where AP23 consistently exhibits higher cleavage activity than WT KmAgo.

Nucleic Acids Preference of Km-AP23 in Cleaving Different DNA and RNA

We further investigate the nucleic acid preference of Km-AP23 by comparing its cleavage activity with WT KmAgo on different guide/target ssDNA/ssRNA, listed in Table S17. The results are displayed in Fig. S63. Km-AP23 shows enhanced cleavage activity than Km-WT on both DNA and RNA when utilizing ssDNA as a guide. However, it demonstrates comparable or reduced cleavage activity on DNA and RNA when employing ssRNA as a guide, which could be attributed to the high binding free energy to the substrates (Fig. S64). The above results suggest that Km-AP23 does

not modify its preference for nucleic acids, as KmAgo exhibits higher cleavage activity when using ssDNA as a guide.

6.3 Bioinformatics and Structural Analyses for Sequential Evaluations

Evolutionary Analysis of KmAgo and Km-APs

We examine the sequence identity of 27 Km-APs to the WT KmAgo, pAgo proteins (excluding the WT KmAgo), other protein families, and among themselves (Fig. S44-Fig. S47). Comparisons between Km-APs and the WT KmAgo, as well as among Km-APs, indicated sequence identity ranging from approximately 48% to 69% and 46% to 80%, respectively. In comparison to all pAgo proteins and proteins from broader families, the sequence identity ranged within 35% and 45%. The conservative patterns preserved by CPDiffusion were validated in Fig. S97. When training with the WT pAgo dataset, CPDiffusion managed to secure the DEDX catalytic sites in the PIWI domain, which is critical for pAgo proteins to achieve cleavage functionality.

Mechanism of the Reduced and Enhanced Cleavage Activity in Km-APs

There are several Km-APs exhibiting notable reduced or enhanced cleavage activity. We thus make interpretations of their mechanism based on their local secondary structure changes in the catalytic region.

Fig. S65 visualizes the area around the catalytic sites for Km-AP9, Km-AP8, and Km-AP19 that exhibit lower activity than the WT KmAgo. In Km-AP8, the E562 in the loop region forms a small alpha helix (highlighted in red), and this structural change might hinder E562 from inserting into the catalytic pocket due to increased steric hindrance. As for Km-AP9 and Km-AP19, there are missing beta sheets near D527 (highlighted in orange and green), leading to a reduction in electrostatic interactions (Table S18 and Fig. S67) and potentially destabilizing the structure of the catalytic pocket.

Fig. S66 investigates the local structure of Km-AP22, Km-AP23, and Km-AP27 around their catalytic motif, where certain AAs within the turn fold into beta-sheets (highlighted in green). This observation indicates the presence of more hydrogen bonds and salt bridges compared to the WT KmAgo (Table S18). The increased interaction around the catalytic sites could enhance the structural stability of the catalytic pockets (Fig. S67), which is crucial for the catalytic motif to cleave the target DNA [24, 27].

6.4 Novel PfAgo Sequence Generation and Evaluation

The same generating, screening, and evaluating procedures were conducted for PfAgo as we did on KmAgo. The pLDDT curves for the selected 15 Pf-APs are visualized in Fig. S68-Fig. S82 in comparison to the WT PfAgo. All 15 experimental Pf-APs have their RMSD lower than 1 Å and TM score higher than 0.9 (Fig. S83), indicating a high structural consistency of WT PfAgo and Pf-APs. Fig. S84 shows the structure of 15 Pf-APs aligned with WT PfAgo, and Fig. S85 compares the electrostatic surface of the MID domain and PIWI domain for WT PfAgo and Pf-APs. These results demonstrate a high similarity of structure and electrostatic surface between WT PfAgo and Pf-APs.

The sequences of Pf-APs are reported in Table S19-S23, for which we conduct wet experiments for activity examination. All Pf-APs are successfully expressed and purified (the SDS-PAGE is shown in Fig. S90. The SAXS demonstrate that the Pf-APs are correctly folded (Fig. S91 and Table S24). The thermostability of Pf-APs is evaluated in Fig. S95, where the melting temperature of Pf-APs is at $\sim 50°C$, lower than that of WT PfAgo (95°C). This can be attributed to the fact that our training dataset primarily consists of pAgo proteins from mesophilic prokaryotes (Fig. S101), which results in Pf-APs adopting temperature properties in mesophilic pAgo proteins. Subsequently, we conduct the ssDNA cleavage assay on KmAgo, PfAgo, and Pf-APs to evaluate the performance of the generated proteins. We then examine the ssDNA cleavage activity of Pf-APs at 45°C, and compare them with the ssDNA cleavage activity of WT PfAgo at both 45°C and 95°C, and WT KmAgo at 45°C. Note that WT PfAgo exhibits its highest activity at 95°C, while KmAgo is totally unfolded at 95°C. As shown in Fig. S92 and Fig. S93, all Pf-APs demonstrate ssDNA cleavage activity. Additionally, Pf-APs have higher cleavage activity than Pf-WT with different ratio of protein:gDNA:tDNA (Fig. S94). Furthermore, all Pf-APs exhibit enhanced cleavage activity compared to PfAgo at 45°C. Among the 15 Pf-APs, 11 show increased cleavage activity compared to KmAgo at 45°C, and 6 out of 15 surpass PfAgo's cleavage activity at 95°C.

We further perform sequence identity analysis on Pf-APs to WT PfAgo, pAgo proteins (excluding the WT PfAgo), other protein families, and among themselves (Fig. S86-Fig. S89). Comparisons between Pf-AP and WT PfAgo as well as among Pf-APs, indicated sequence identity ranging approximately $57 - 62\%$ and $50 - 60\%$, respectively. In comparison to all pAgo proteins and proteins from broader families, the sequence identity ranged within 41% and 44%. Similar to KmAgo, the generated Pf-APs also well-capture the conservative patterns in WT PfAgo (Fig. S96 and Fig. S98). Note that the conservative sites (excluding the catalytic sites DEDX) are based on alignments to the pAgo family dataset, where the residue conservation scores are provided in Fig. S99.

7 Related Work

The diffusion probabilistic models have demonstrated promising performance in generating samples from continuous-time spaces, as seen in applications such as image synthesis [28] and audio generation [29]. In the context of molecule design, this technique has been utilized to simulate conformations for small molecules or molecule motifs [30–32].

Despite its effectiveness in generating continuous outputs, the discrete nature of the AA space poses challenges for the rapid development of protein sequence generation using diffusion models. Existing methods for sequence generation predominantly rely on protein language models or structure-based representation learning. Methods falling into the former category leverage protein language models to infer pairwise spatial relationships of AAs based on evolutionary information. These models are typically pre-trained on large datasets of protein sequences and generate novel sequences through autoregressive prediction [33–35] or by following a BERT-style

generative framework with masked language modeling [36–38]. In contrast, structure-based approaches to protein design formulate fixed-backbone protein design as a conditional sequence generation problem. They predict node labels representing AA types using invariant or equivariant graph neural networks [3, 11, 39–41].

Although diffusion models have not been applied to protein sequence generation until recently [42, 43], they are intriguingly connected to many existing methodologies. For example, masked language models can be viewed as discrete diffusion-absorbing models when trained with a parameterized objective. Autoregressive models, on the other hand, can be perceived as deterministic diffusion processes [44]. While autoregressive models assign a conditional distribution to each token, the overall dependency along the entire AA sequence is recast through independently executed diffusion processes. In comparison, diffusion probabilistic models, as an extension of Langevin Monte Carlo with learnable score functions, employ an iterative prediction methodology that generates less noisy samples, demonstrating potential in capturing the inherent diversity in real data distributions. Moreover, it is possible for a diffusion-based framework to insert heuristics through generating conditions to guide the generation process with domain prior knowledge. This unique characteristic further underscores the promising role that diffusion models could play in advancing the field of protein sequence design.

8 Outlook of CPDiffusion

This study introduces a novel pipeline for generating sequences tailored to a given protein backbone. Central to this workflow is a diffusion-based generative model known as CPDiffusion, designed to generate protein sequences while adhering to specified conditions, such as immutable positions and their corresponding amino acid types. We have showcased the remarkable potential of this generative pipeline through its application to two endonucleases naturally active at distinct temperatures. The newly generated sequences, despite exhibiting considerably low sequence identities, display a high likelihood of surpassing the activity of their associated WT protein templates. This suggests the promising prospect of extending our method to design novel proteins in other families with lower sequence identity and enhanced functionality.

Although our primary focus is on the design of endonucleases, this section delves into potential future research and application directions, as well as ethical considerations associated with the proposed generative technique in a broader context.

8.1 Potential Applications of CPDiffusion in Nucleic Acid-based Diagnostics and Protein Library Expansion

Designing endonucleases with enhanced cleavage activity not only facilitates gene editing but also holds potential applications in isothermal nucleic acid detection. Traditionally, Ago-based nucleic acid detection has been predominantly associated with thermophilic proteins due to their high cleavage activity [45, 46]. However, this method necessitates operation at high temperatures, requiring additional heat sources and resulting in non-portable detection equipment. In contrast, mesophilic Ago-based detection allows testing at moderate temperatures with portable equipment, but its

lower enzymatic activity poses challenges for fast diagnostics [47, 48]. Our CPDiffusion-generated mesophilic Ago proteins exhibit excellent cleavage activity, positioning them as potential key enzymes for isothermal nucleic acid detection.

Moreover, if protein design methods demonstrate a high success rate in generating functional protein sequences, they offer a new avenue for protein engineering and the potential expansion of the sequence library and protein fitness landscape. Traditional approaches involve modifying natural protein sequences to enhance their functions by mutating one or more amino acids, improving aspects such as thermostability, catalytic activity, and resistance to acidity or alkalinity [49, 50]. However, even with significant time and financial investment in multi-round mutation, these mutagenesis approaches may still fall short of meeting practical needs. In contrast, CPDiffusion provides the possibility to generate novel sequences that do not exist in nature. These sequences could exhibit high activity, stability, diverse substrate selectivity, *etc*, compared to naturally existing ones. The generated sequences offer fresh blueprints, accelerating subsequent evolutionary processes to engineer proteins that meet practical demands. Furthermore, protein sequences generated by CPDiffusion expand the sequence library, providing researchers with a more diverse range of options to explore for desired protein functions.

8.2 Scalability of CPDiffusion in Protein Generation

In this work, we have demonstrated the reliability of designed sequences by CPDiffusion using two types of Argonaute proteins, KmAgo and PfAgo, each possessing multiple domains and complex functions. The positive results suggest the power of CPDiffusion in generating novel protein sequences for a given backbone structure to achieve enhanced functionality. As a general technique, we expect this pipeline to be applied to proteins from different families in the future. We attribute the success of our design, based on our exploration in this study, to the quality of the additional training dataset and the conditions provided to the model.

The former is pivotal for the model to understand the central connection between sequence-structure-function for a particular protein family. In this study, the pAgo dataset we employed provides a diverse set of pAgo proteins from different physiological temperatures, species, nucleic acid preferences, and biofunctions. Nevertheless, they share similar conserved structures with low sequence similarities (for example, the structure similarity and sequence identity between templates (KmAgo and PfAgo) and other pAgo proteins are shown in Fig. S100). Such construction patterns implied in the training dataset gently guide the generated protein sequences of a particular protein family. Consequently, the model efficiently learns the essential construction patterns for pAgo proteins, such as the conservative patterns and the sequence-function projections.

The other key factor for successfully designing and running the model, as mentioned previously, is the condition provided to guide the generation. A representative example is the secondary structure encoded into the model during the denoising phase, which actively contributes to the protein's functionality. For instance, transmembrane α-helices play essential roles in protein functions, such as passing ions or other molecules and transmitting a signal across the membrane. Especially for secondary structures

around the functional region, as we have investigated previously for the inactive and highly-functional Km-APs, they could potentially become the key to a designed protein being functional.

8.3 Broader Impact of CPDiffusion in Gene Editing and Protein Design

We have discussed the potential application of our developed protein sequence generation technique for promoting the development of protein design, such as enzyme engineering. While this method holds substantial potential for facilitating scientific research and biological discoveries, the potential risks of developing and applying such techniques should also be noted.

From the perspective of designing new endonucleases for biomedical applications, such as gene editing, our study, while not proposing a powerful editing tool ready for use, does have the potential to design prototypes of novel endonucleases with different properties in theory, such as nucleic acid preferences. As observed in this study, when training the current version of CPDiffusion with WT proteins from the same family, it is very unlikely to directly generate proteins with new functionalities on top of enhancing the existing enzymatic activity. On the other hand, when training on a protein family with different functionalities, it is not totally impossible for the generated sequences to acquire certain properties of the majority of training proteins. For instance, the Pf-APs generated from CPDiffusion trained on a large portion of pAgo proteins living in environments of moderate temperatures have a reduced enzymatic temperature. However, a steep hurdle exists in successfully designing such proteins. An immediate obstacle would be preparing a proper training dataset, which has been proven to be the key to generating high-quality novel sequences. Also, the expression and examination protocol of the novel sequences might be totally different, making it even harder to develop and validate before deploying in practice.

References

[1] Ganea, O.-E., Huang, X., Bunne, C., Bian, Y., Barzilay, R., Jaakkola, T.S., Krause, A.: Independent SE(3)-equivariant models for end-to-end rigid protein docking. In: International Conference on Learning Representations (2021)

[2] Zhou, B., Lv, O., Yi, K., Xiong, X., Tan, P., Hong, L., Wang, Y.G.: Accurate and definite mutational effect prediction with lightweight equivariant graph neural networks. arXiv:2304.08299 (2023)

[3] Ingraham, J., Garg, V., Barzilay, R., Jaakkola, T.: Generative models for graph-based protein design. Advances in Neural Information Processing Systems **32** (2019)

[4] Satorras, V.G., Hoogeboom, E., Welling, M.: E(n) equivariant graph neural networks. In: International Conference on Machine Learning, pp. 9323–9332 (2021)

[5] Lugmayr, A., Danelljan, M., Romero, A., Yu, F., Timofte, R., Van Gool, L.: Repaint: Inpainting using denoising diffusion probabilistic models. In: Proceedings of the IEEE/CVF Conference on Computer Vision and Pattern Recognition, pp. 11461–11471 (2022)

[6] Henikoff, S., Henikoff, J.G.: Amino acid substitution matrices from protein blocks. Proceedings of the National Academy of Sciences **89**(22), 10915–10919 (1992)

[7] Trivedi, R., Nagarajaram, H.A.: Substitution scoring matrices for proteins-an overview. Protein Science **29**(11), 2150–2163 (2020)

[8] Song, J., Meng, C., Ermon, S.: Denoising diffusion implicit models. In: International Conference on Learning Representations

[9] Orengo, C., Michie, A., Jones, S., Jones, D., Swindells, M., Thornton, J.: CATH – a hierarchic classification of protein domain structures. Structure **5**(8), 1093–1109 (1997) https://doi.org/10.1016/S0969-2126(97)00260-8

[10] Qi, Y., Zhang, J.Z.: Densecpd: improving the accuracy of neural-network-based computational protein sequence design with densenet. Journal of chemical information and modeling **60**(3), 1245–1252 (2020)

[11] Jing, B., Eismann, S., Suriana, P., Townshend, R.J.L., Dror, R.: Learning from protein structure with geometric vector perceptrons. In: International Conference on Learning Representations (2021)

[12] Tan, C., Gao, Z., Xia, J., Li, S.Z.: Generative de novo protein design with global context. arXiv preprint arXiv:2204.10673 (2022)

[13] Gao, Z., Tan, C., Li, S.Z.: Alphadesign: A graph protein design method and benchmark on alphafolddb. arXiv preprint arXiv:2202.01079 (2022)

[14] Dauparas, J., Anishchenko, I., Bennett, N., Bai, H., Ragotte, R.J., Milles, L.F., Wicky, B.I., Courbet, A., Haas, R.J., Bethel, N., *et al.*: Robust deep learning–based protein sequence design using proteinmpnn. Science **378**(6615), 49–56 (2022)

[15] Gao, Z., Tan, C., Li, S.Z.: Pifold: Toward effective and efficient protein inverse folding. In: International Conference on Learning Representations (2023). https://openreview.net/forum?id=oMsN9TYwJ0j

[16] Nichol, A.Q., Dhariwal, P.: Improved denoising diffusion probabilistic models. In: International Conference on Machine Learning, pp. 8162–8171 (2021). PMLR

[17] Kingma, D.P., Ba, J.: ADAM: A method for stochastic optimization. In: Proceedings of International Conference on Learning Representation (International Conference on Learning Representations) (2015)

[18] Rost, B.: Twilight zone of protein sequence alignments. Protein engineering **12**(2), 85–94 (1999)

[19] Gong, H., Zhang, H., Zhu, J., Wang, C., Sun, S., Zheng, W.-M., Bu, D.: Improving prediction of burial state of residues by exploiting correlation among residues. BMC bioinformatics **18**(3), 165–175 (2017)

[20] Maaten, L., Hinton, G.: Visualizing data using t-sne. Journal of machine learning research **9**(11) (2008)

[21] Wu, K.E., Yang, K.K., Berg, R.v.d., Zou, J.Y., Lu, A.X., Amini, A.P.: Protein structure generation via folding diffusion. arXiv preprint arXiv:2209.15611 (2022)

[22] Kipf, T.N., Welling, M.: Semi-supervised classification with graph convolutional networks. In: ICLR (2017)

[23] Ryazansky, S., Kulbachinskiy, A., Aravin, A.A.: The expanded universe of prokaryotic argonaute proteins. MBio **9**(6), 10–1128 (2018)

[24] Lisitskaya, L., Aravin, A.A., Kulbachinskiy, A.: Dna interference and beyond: structure and functions of prokaryotic argonaute proteins. Nature communications **9**(1), 5165 (2018)

[25] Xu, J., Zhang, Y.: How significant is a protein structure similarity with tm-score= 0.5? Bioinformatics **26**(7), 889–895 (2010)

[26] Sander, C., Schneider, R.: Database of homology-derived protein structures and the structural meaning of sequence alignment. Proteins: Structure, Function, and Bioinformatics **9**(1), 56–68 (1991)

[27] Sheng, G., Zhao, H., Wang, J., Rao, Y., Tian, W., Swarts, D.C., Oost, J., Patel, D.J., Wang, Y.: Structure-based cleavage mechanism of thermus thermophilus argonaute dna guide strand-mediated dna target cleavage. Proceedings of the National Academy of Sciences **111**(2), 652–657 (2014)

[28] Rombach, R., Blattmann, A., Lorenz, D., Esser, P., Ommer, B.: High-resolution image synthesis with latent diffusion models. In: Proceedings of the IEEE/CVF Conference on Computer Vision and Pattern Recognition, pp. 10684–10695 (2022)

[29] Yang, D., Yu, J., Wang, H., Wang, W., Weng, C., Zou, Y., Yu, D.: Diffsound: Discrete diffusion model for text-to-sound generation. IEEE/ACM Transactions on Audio, Speech, and Language Processing (2023)

[30] Jing, B., Corso, G., Chang, J., Barzilay, R., Jaakkola, T.S.: Torsional diffusion for molecular conformer generation. In: Oh, A.H., Agarwal, A., Belgrave, D., Cho, K. (eds.) Advances in Neural Information Processing Systems (2022). https:

//openreview.net/forum?id=w6fj2r62r_H

[31] Trippe, B.L., Yim, J., Tischer, D., Baker, D., Broderick, T., Barzilay, R., Jaakkola, T.S.: Diffusion probabilistic modeling of protein backbones in 3d for the motif-scaffolding problem. In: International Conference on Learning Representations (2023). https://openreview.net/forum?id=6TxBxqNME1Y

[32] Corso, G., Stärk, H., Jing, B., Barzilay, R., Jaakkola, T.S.: Diffdock: Diffusion steps, twists, and turns for molecular docking. In: The Eleventh International Conference on Learning Representations (2023). https://openreview.net/forum?id=kKF8_K-mBbS

[33] Madani, A., Krause, B., Greene, E.R., Subramanian, S., Mohr, B.P., Holton, J.M., Olmos Jr, J.L., Xiong, C., Sun, Z.Z., Socher, R., et al.: Large language models generate functional protein sequences across diverse families. Nature Biotechnology, 1–8 (2023)

[34] Notin, P., Dias, M., Frazer, J., Hurtado, J.M., Gomez, A.N., Marks, D., Gal, Y.: Tranception: protein fitness prediction with autoregressive transformers and inference-time retrieval. In: International Conference on Machine Learning, pp. 16990–17017 (2022). PMLR

[35] Shin, J.-E., Riesselman, A.J., Kollasch, A.W., McMahon, C., Simon, E., Sander, C., Manglik, A., Kruse, A.C., Marks, D.S.: Protein design and variant prediction using autoregressive generative models. Nature Communications **12**(1), 2403 (2021)

[36] Lin, Z., Akin, H., Rao, R., Hie, B., Zhu, Z., Lu, W., Smetanin, N., Verkuil, R., Kabeli, O., Shmueli, Y., *et al.*: Evolutionary-scale prediction of atomic-level protein structure with a language model. Science **379**(6637), 1123–1130 (2023)

[37] Meier, J., Rao, R., Verkuil, R., Liu, J., Sercu, T., Rives, A.: Language models enable zero-shot prediction of the effects of mutations on protein function. In: Advances in Neural Information Processing Systems, vol. 34, pp. 29287–29303 (2021)

[38] Rives, A., Meier, J., Sercu, T., Goyal, S., Lin, Z., Liu, J., Guo, D., Ott, M., Zitnick, C.L., Ma, J., *et al.*: Biological structure and function emerge from scaling unsupervised learning to 250 million protein sequences. Proceedings of the National Academy of Sciences **118**(15), 2016239118 (2021)

[39] Hsu, C., Verkuil, R., Liu, J., Lin, Z., Hie, B., Sercu, T., Lerer, A., Rives, A.: Learning inverse folding from millions of predicted structures. In: Chaudhuri, K., Jegelka, S., Song, L., Szepesvari, C., Niu, G., Sabato, S. (eds.) Proceedings of the 39th International Conference on Machine Learning. Proceedings of Machine Learning Research, vol. 162, pp. 8946–8970. PMLR, ??? (2022)

[40] Zhou, B., Zheng, L., Wu, B., Tan, Y., Lv, O., Yi, K., Fan, G., Hong, L.: Protein engineering with lightweight graph denoising neural networks. bioRxiv, 2023–11 (2023)

[41] Tan, Y., Zhou, B., Zheng, L., Fan, G., Hong, L.: Semantical and topological protein encoding toward enhanced bioactivity and thermostability. bioRxiv, 2023–12 (2023)

[42] Watson, J.L., Juergens, D., Bennett, N.R., Trippe, B.L., Yim, J., Eisenach, H.E., Ahern, W., Borst, A.J., Ragotte, R.J., Milles, L.F., et al.: De novo design of protein structure and function with rfdiffusion. Nature, 1–3 (2023)

[43] Yi, K., Zhou, B., Shen, Y., Lio, P., Wang, Y.G.: Graph denoising diffusion for inverse protein folding. In: Thirty-seventh Conference on Neural Information Processing Systems (2023)

[44] Austin, J., Johnson, D.D., Ho, J., Tarlow, D., Berg, R.: Structured denoising diffusion models in discrete state-spaces. Advances in Neural Information Processing Systems **34**, 17981–17993 (2021)

[45] Song, J., Hegge, J.W., Mauk, M.G., Chen, J., Till, J.E., Bhagwat, N., Azink, L.T., Peng, J., Sen, M., Mays, J., *et al.*: Highly specific enrichment of rare nucleic acid fractions using thermus thermophilus argonaute with applications in cancer diagnostics. Nucleic acids research **48**(4), 19–19 (2020)

[46] Liu, Q., Guo, X., Xun, G., Li, Z., Chong, Y., Yang, L., Wang, H., Zhang, F., Luo, S., Cui, L., *et al.*: Argonaute integrated single-tube pcr system enables supersensitive detection of rare mutations. Nucleic acids research **49**(13), 75–75 (2021)

[47] Li, X., Dong, H., Guo, X., Huang, F., Xu, X., Li, N., Yang, Y., Yao, T., Feng, Y., Liu, Q.: Mesophilic argonaute-based isothermal detection of sars-cov-2. Frontiers in microbiology **13**, 957977 (2022)

[48] Marsic, T., Gundra, S.R., Wang, Q., Aman, R., Mahas, A., Mahfouz, M.M.: Programmable site-specific dna double-strand breaks via pna-assisted prokaryotic argonautes. Nucleic Acids Research **51**(17), 9491–9506 (2023)

[49] Currin, A., Swainston, N., Day, P.J., Kell, D.B.: Synthetic biology for the directed evolution of protein biocatalysts: navigating sequence space intelligently. Chemical Society Reviews **44**(5), 1172–1239 (2015)

[50] Yang, K.K., Wu, Z., Arnold, F.H.: Machine-learning-guided directed evolution for protein engineering. Nature methods **16**(8), 687–694 (2019)

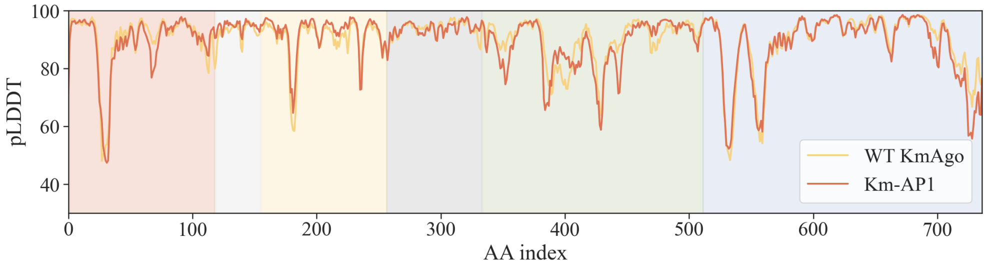

Fig. S8: Comparison of pLDDT of WT KmAgo and Km-AP1.

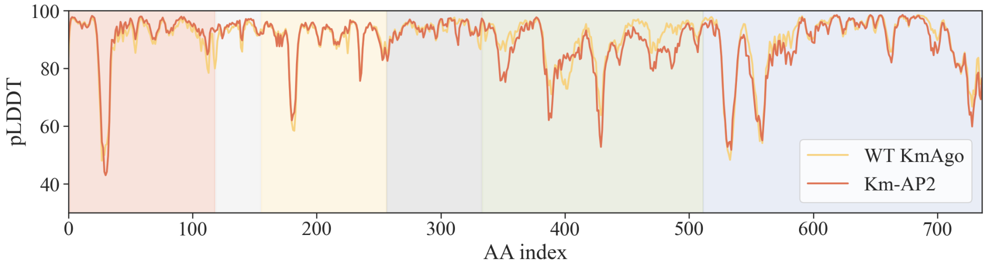

Fig. S9: Comparison of pLDDT of WT KmAgo and Km-AP2.

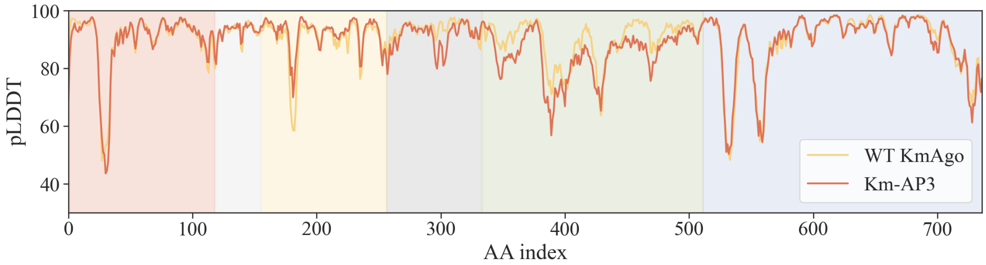

Fig. S10: Comparison of pLDDT of WT KmAgo and Km-AP3.

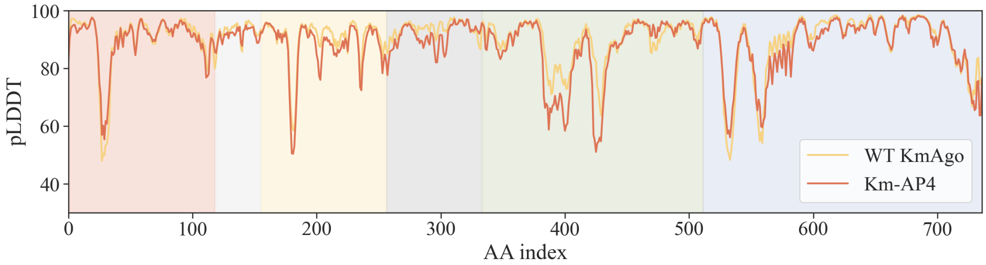

Fig. S11: Comparison of pLDDT of WT KmAgo and Km-AP4.

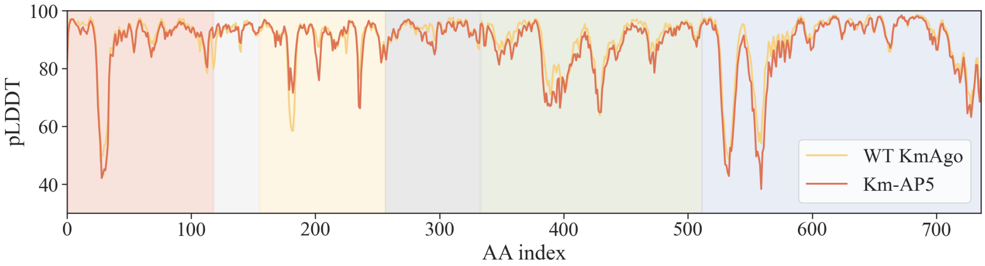

Fig. S12: Comparison of pLDDT of WT KmAgo and Km-AP5.

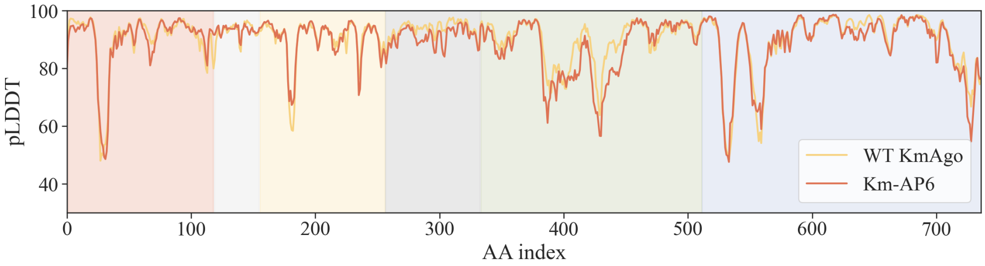

Fig. S13: Comparison of pLDDT of WT KmAgo and Km-AP6.

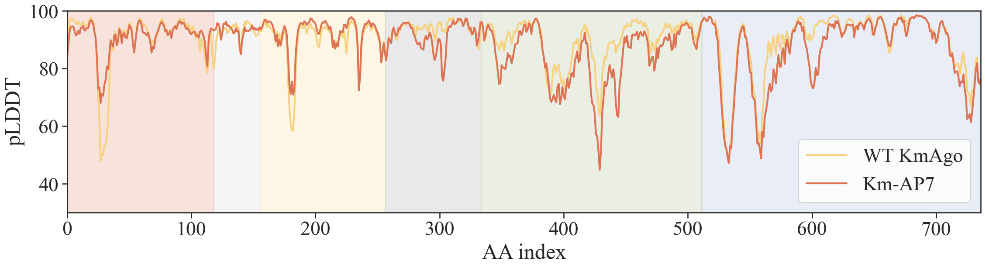

Fig. S14: Comparison of pLDDT of WT KmAgo and Km-AP7.

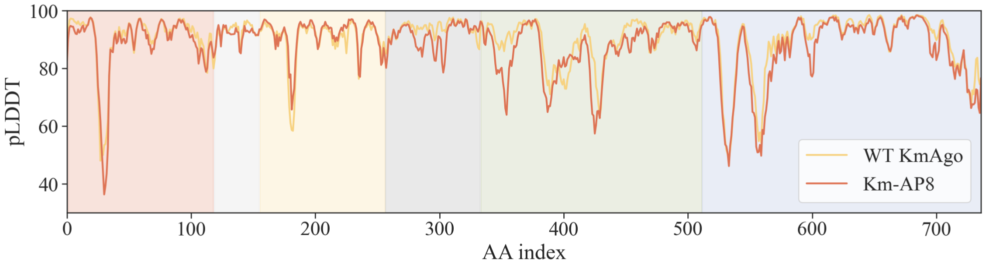

Fig. S15: Comparison of pLDDT of WT KmAgo and Km-AP8.

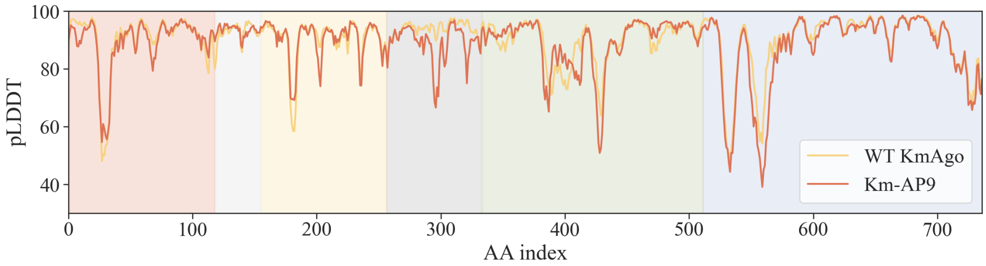

Fig. S16: Comparison of pLDDT of WT KmAgo and Km-AP9.

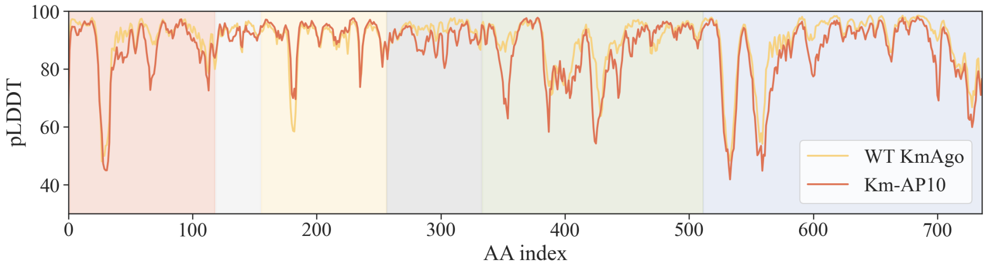

Fig. S17: Comparison of pLDDT of WT KmAgo and Km-AP10.

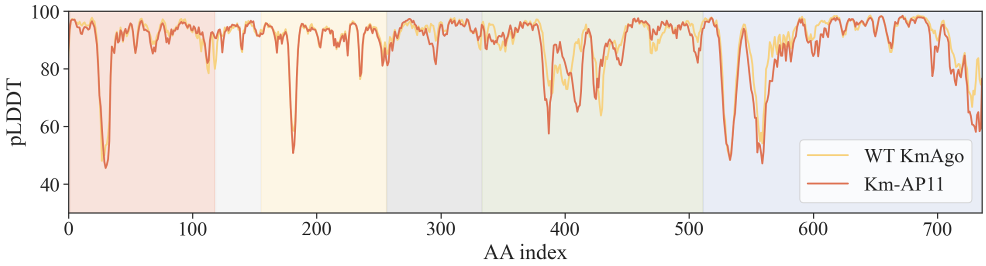

Fig. S18: Comparison of pLDDT of WT KmAgo and Km-AP11.

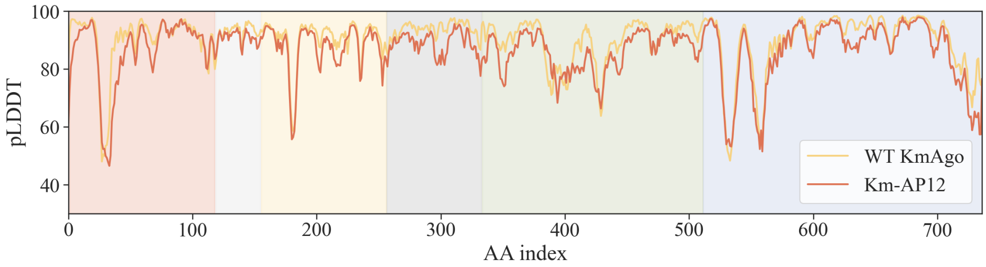

Fig. S19: Comparison of pLDDT of WT KmAgo and Km-AP12.

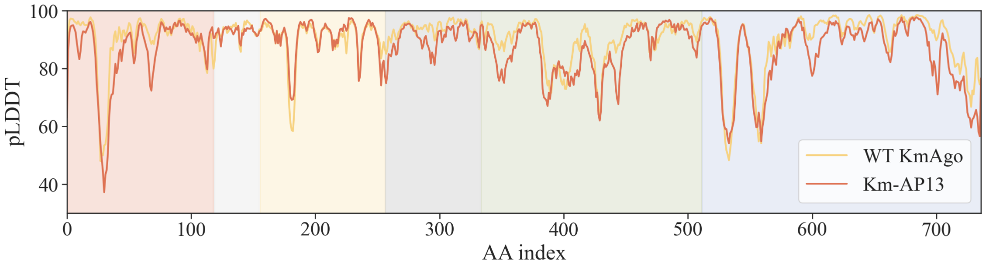

Fig. S20: Comparison of pLDDT of WT KmAgo and Km-AP13.

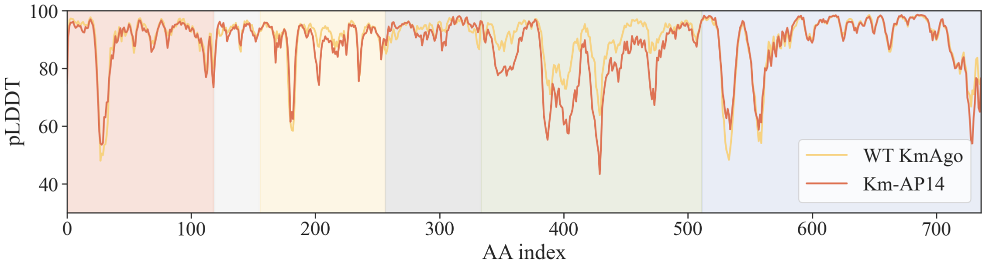

Fig. S21: Comparison of pLDDT of WT KmAgo and Km-AP14.

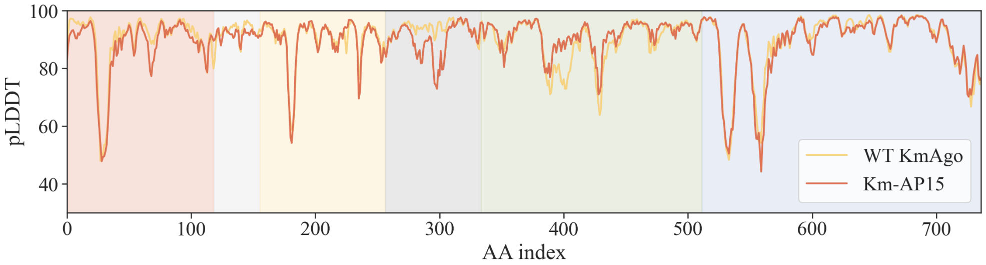

Fig. S22: Comparison of pLDDT of WT KmAgo and Km-AP15.

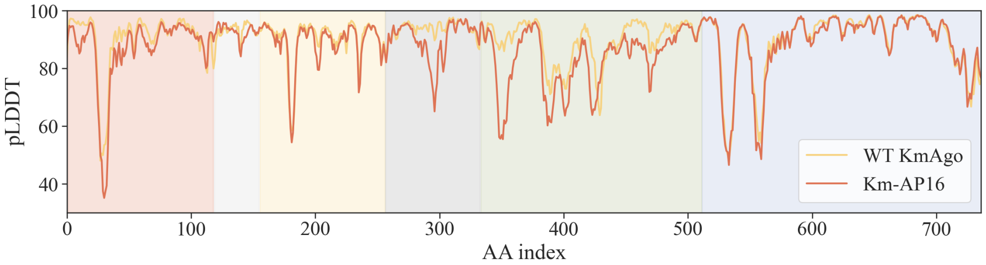

Fig. S23: Comparison of pLDDT of WT KmAgo and Km-AP16.

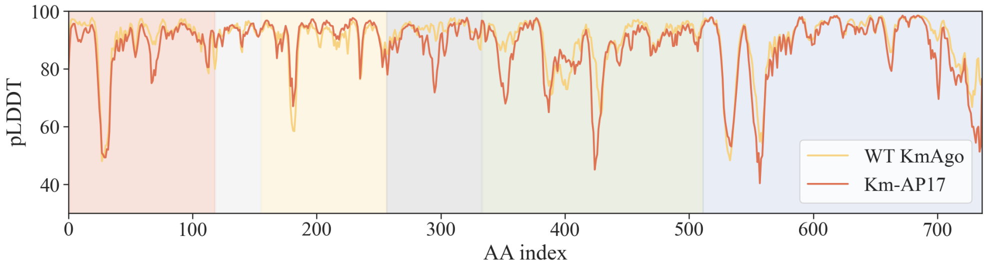

Fig. S24: Comparison of pLDDT of WT KmAgo and Km-AP17.

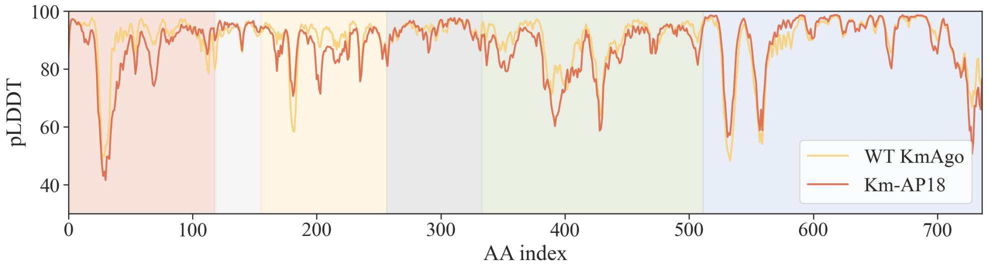

Fig. S25: Comparison of pLDDT of WT KmAgo and Km-AP18.

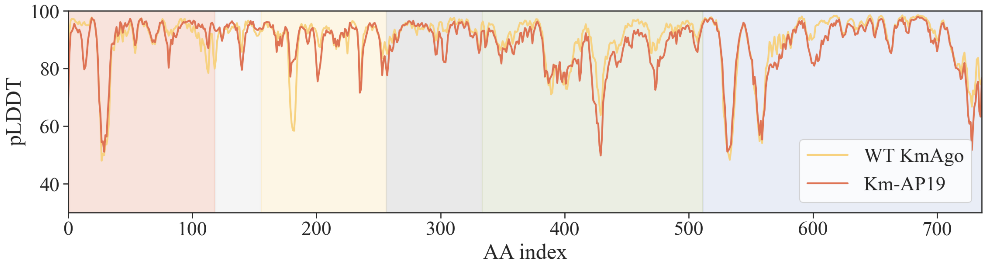

Fig. S26: Comparison of pLDDT of WT KmAgo and Km-AP19.

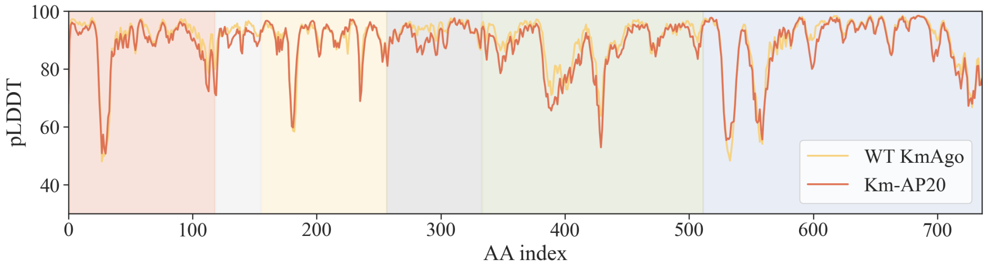

Fig. S27: Comparison of pLDDT of WT KmAgo and Km-AP20.

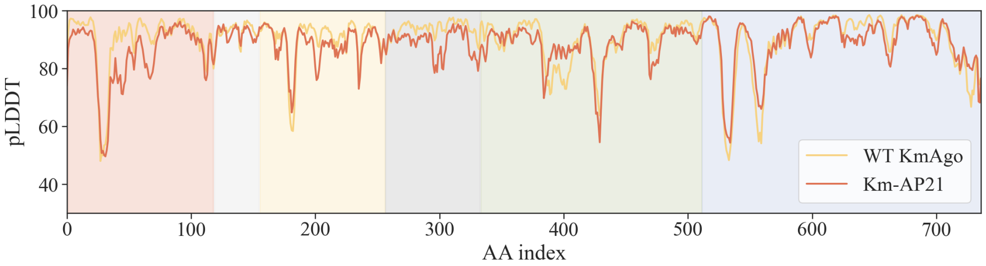

Fig. S28: Comparison of pLDDT of WT KmAgo and Km-AP21.

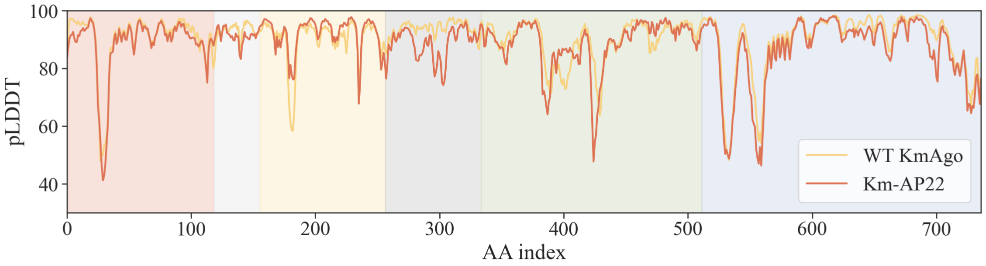

Fig. S29: Comparison of pLDDT of WT KmAgo and Km-AP22.

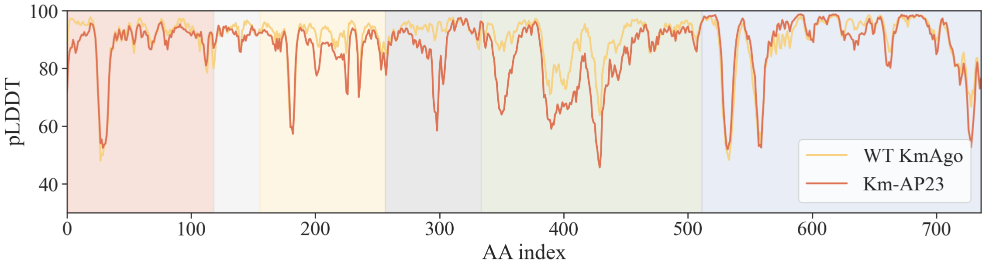

Fig. S30: Comparison of pLDDT of WT KmAgo and Km-AP23.

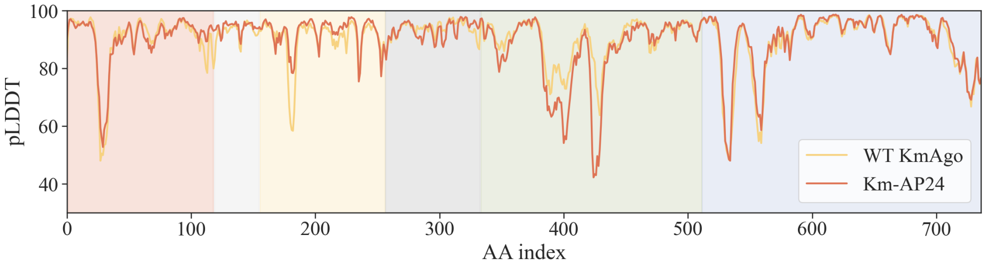

Fig. S31: Comparison of pLDDT of WT KmAgo and Km-AP24.

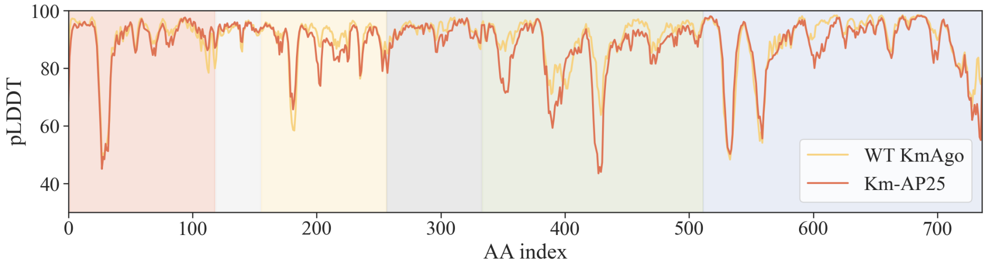

Fig. S32: Comparison of pLDDT of WT KmAgo and Km-AP25.

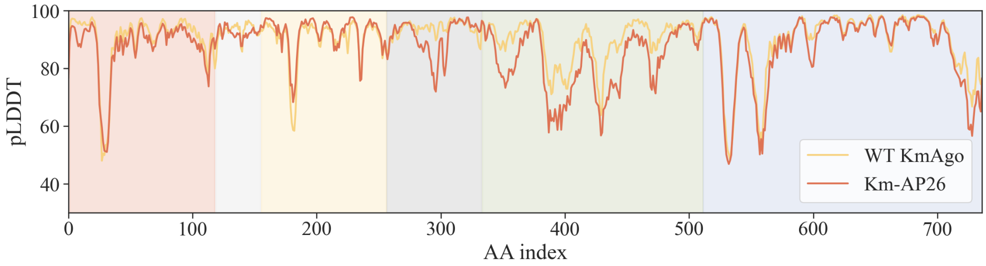

Fig. S33: Comparison of pLDDT of WT KmAgo and Km-AP26.

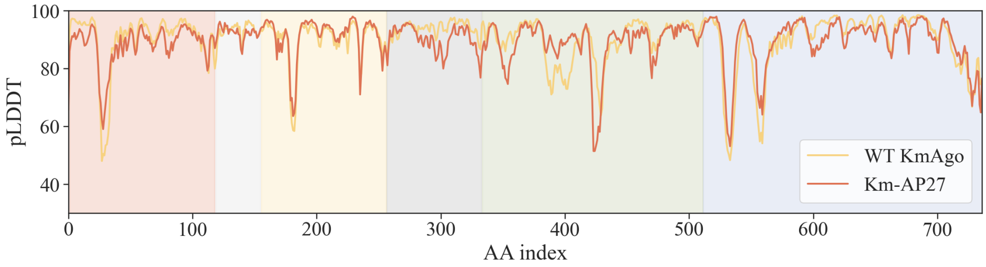

Fig. S34: Comparison of pLDDT of WT KmAgo and Km-AP27.

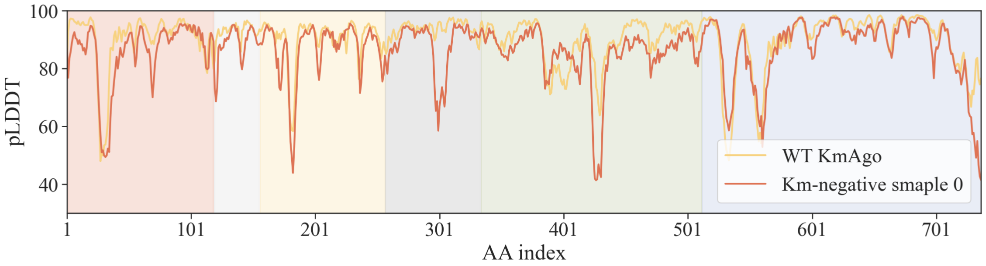

Fig. S35: Comparison of pLDDT of WT KmAgo and negative sample (No.0).

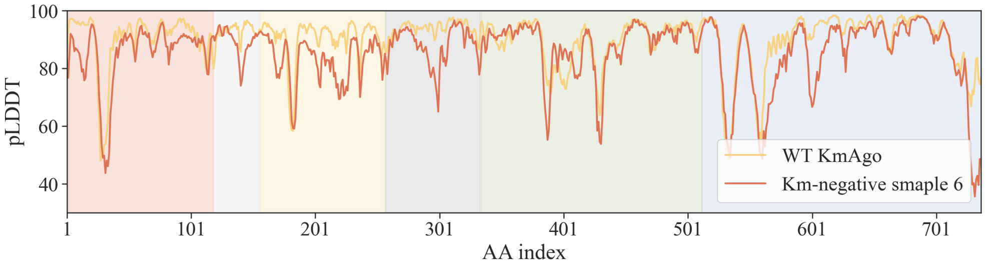

Fig. S36: Comparison of pLDDT of WT KmAgo and negative sample (No.6).

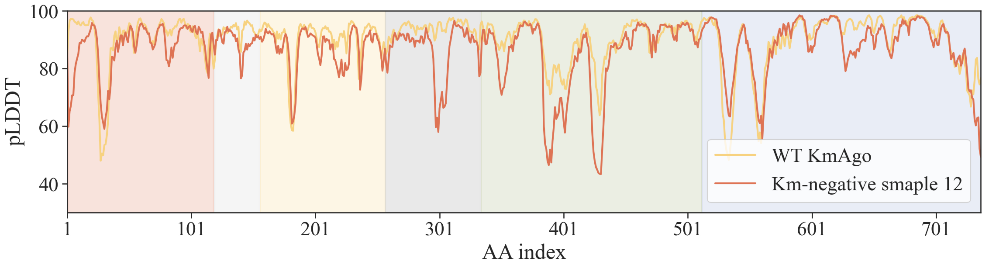

Fig. S37: Comparison of pLDDT of WT KmAgo and negative sample (No.12).

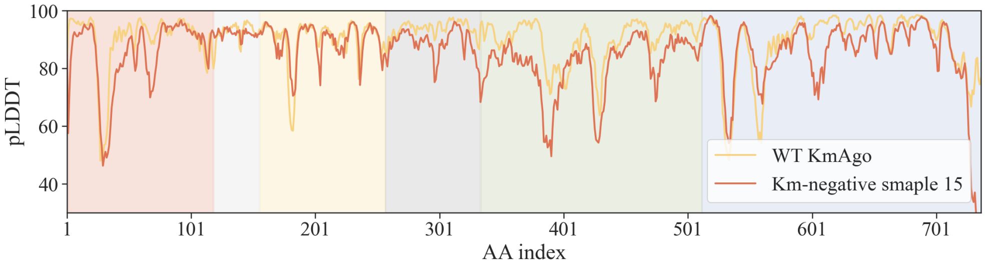

Fig. S38: Comparison of pLDDT of WT KmAgo and negative sample (No.15).

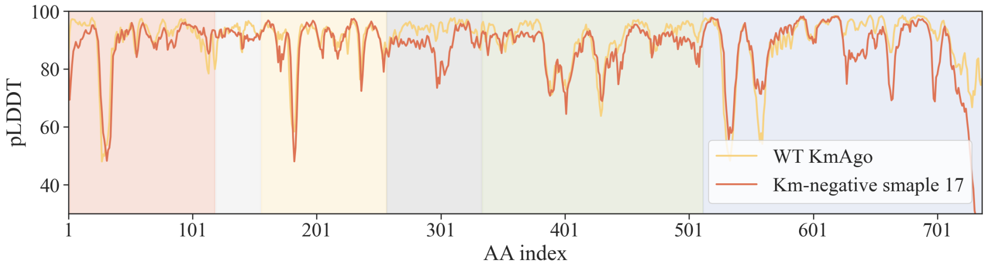

Fig. S39: Comparison of pLDDT of WT KmAgo and negative sample (No.17).

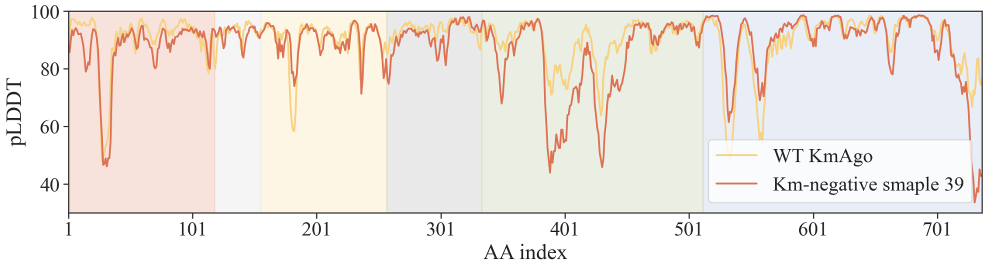

Fig. S40: Comparison of pLDDT of WT KmAgo and negative sample (No.39).

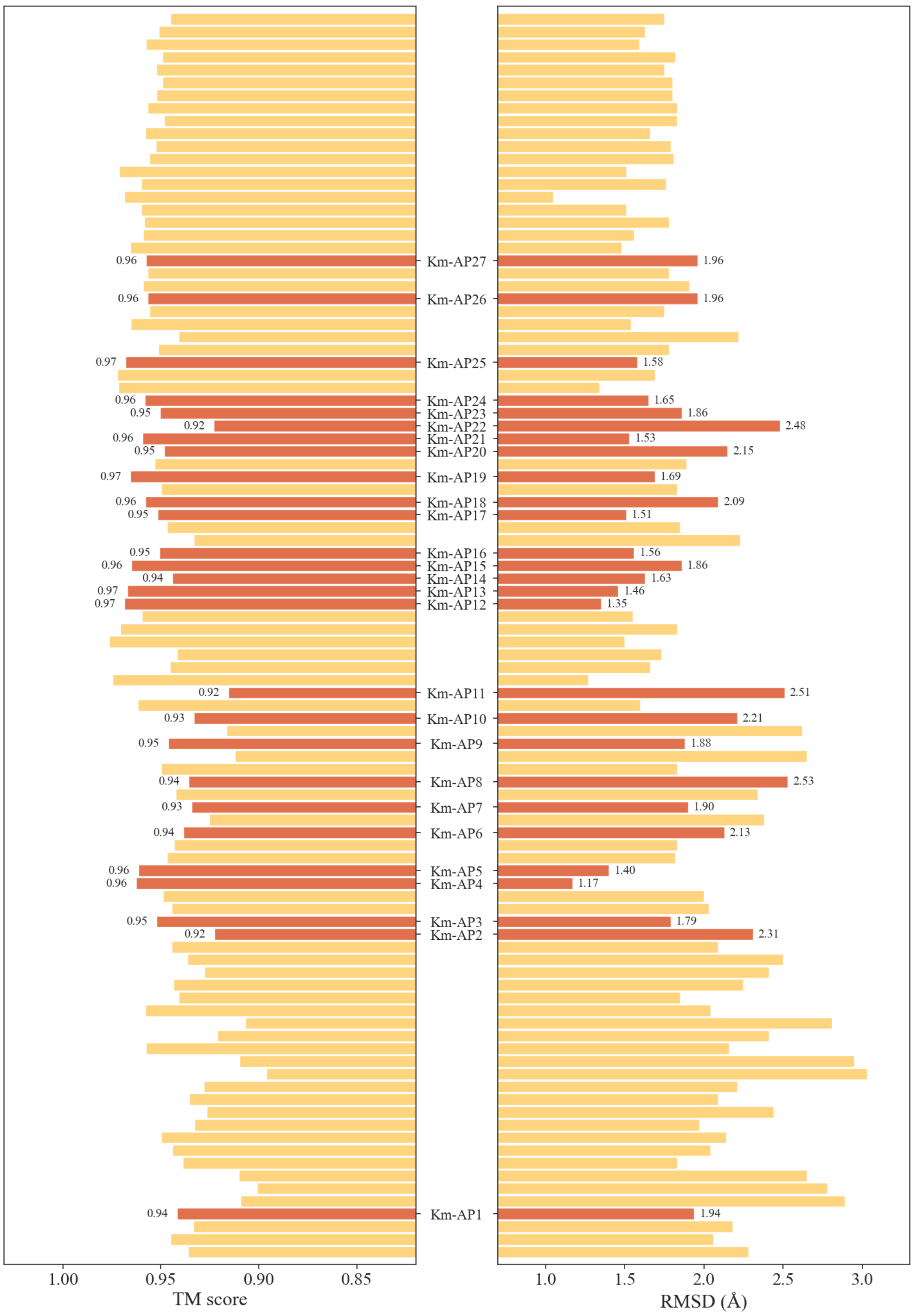

Fig. S41: RMSD (right) and TM score (left) of all 100 generated KmAgo sequences. The 27 Km-APs are highlighted in red.

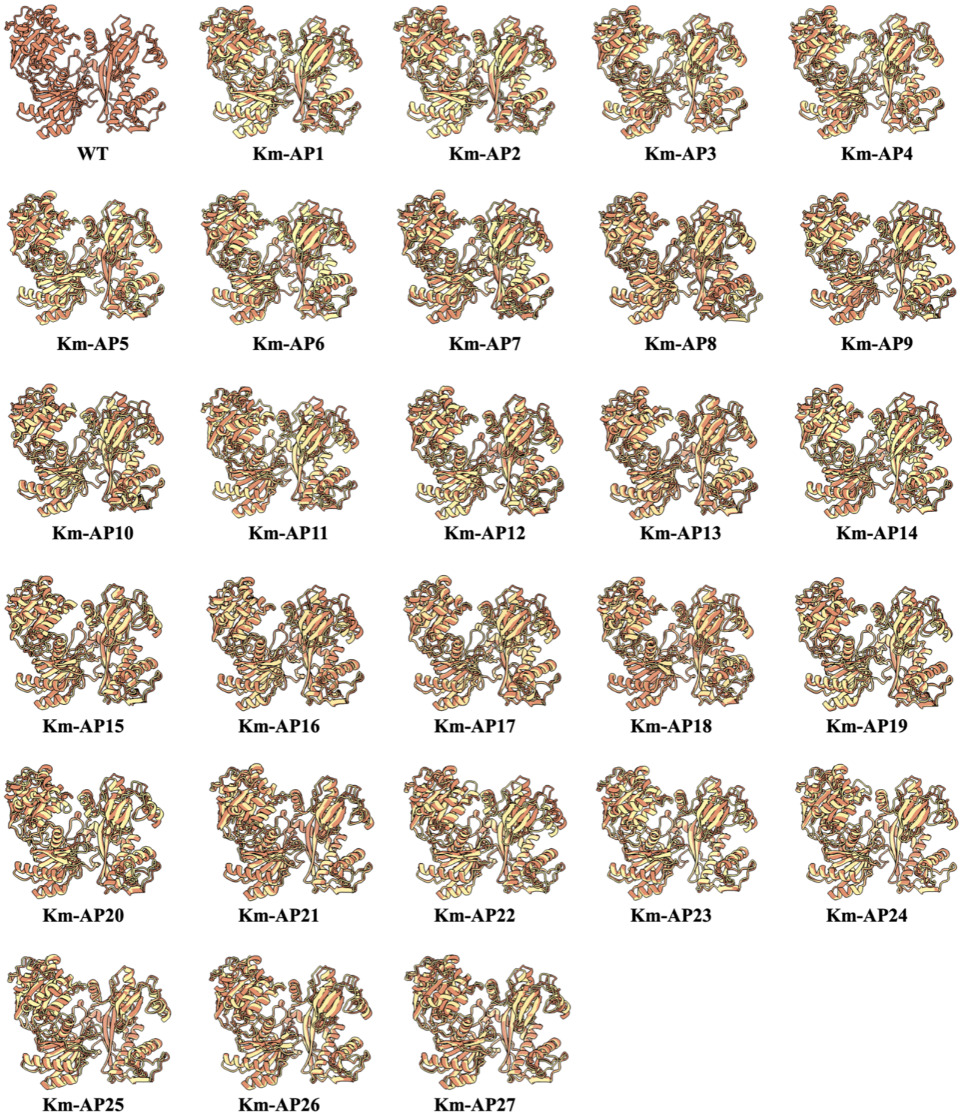

Fig. S42: Structural comparison of KmAgo and Km-APs folded by AlphaFold2.

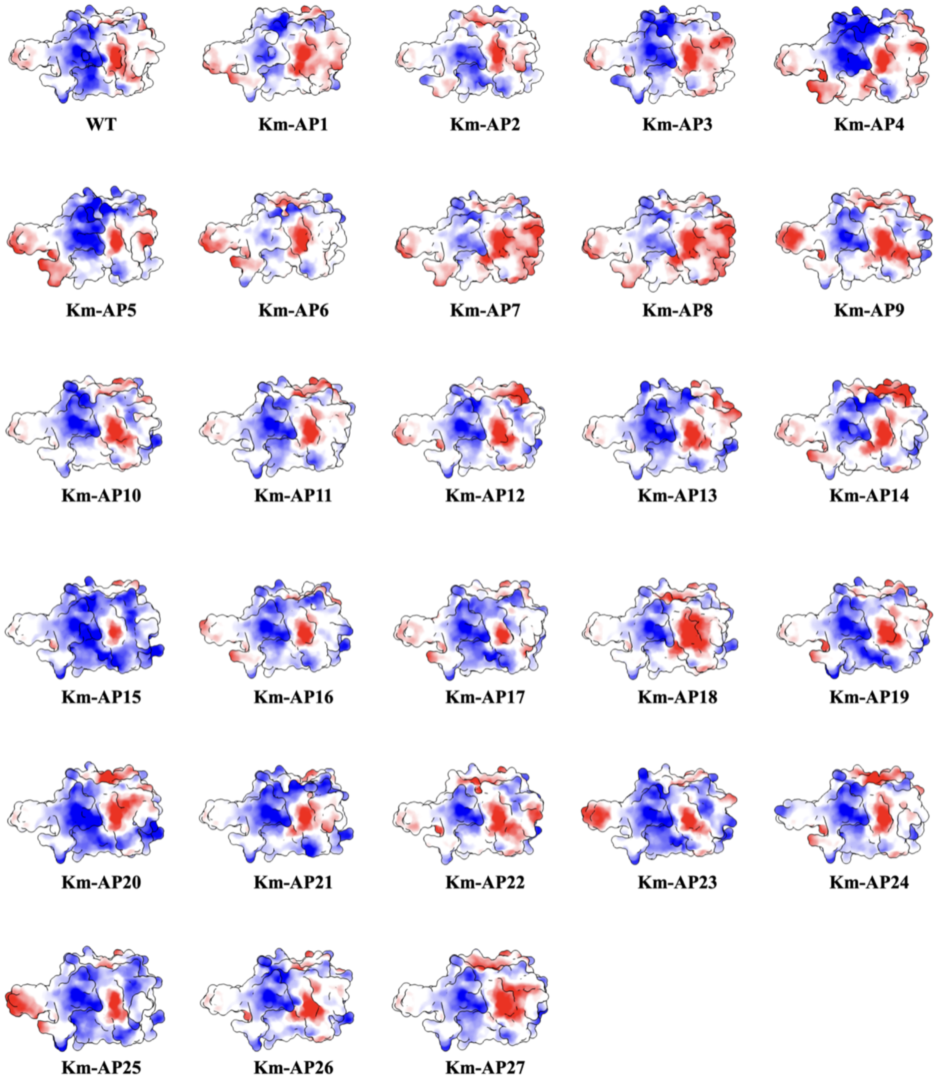

Fig. S43: The electrostatic surface of MID domain and PIWI domain in KmAgo and Km-APs.

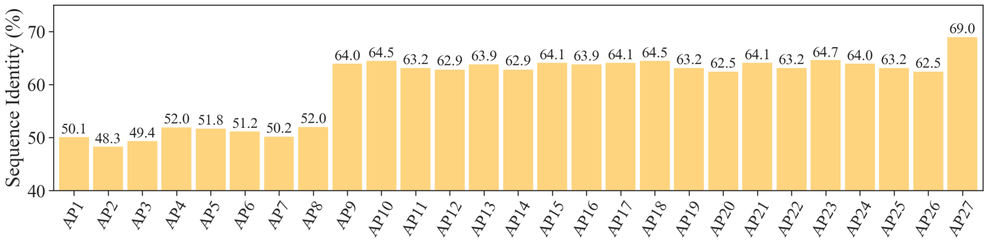

Fig. S44: Sequence identities of Km-APs with the WT KmAgo.

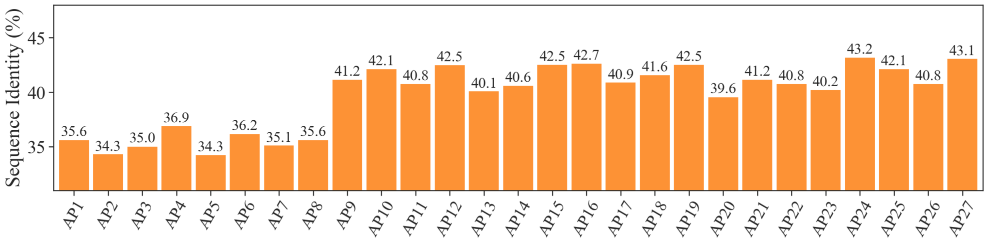

Fig. S45: Sequence identities of Km-APs with the most similar pAgo proteins (excluding WT KmAgo) in the training dataset.

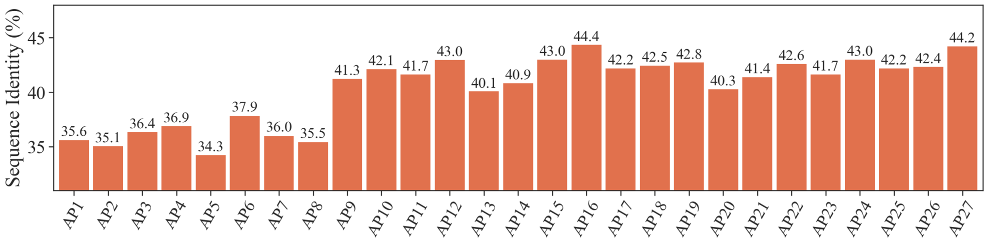

Fig. S46: Sequence identities of Km-APs with the most similar protein sequence in NCBI NR (excluding WT KmAgo).

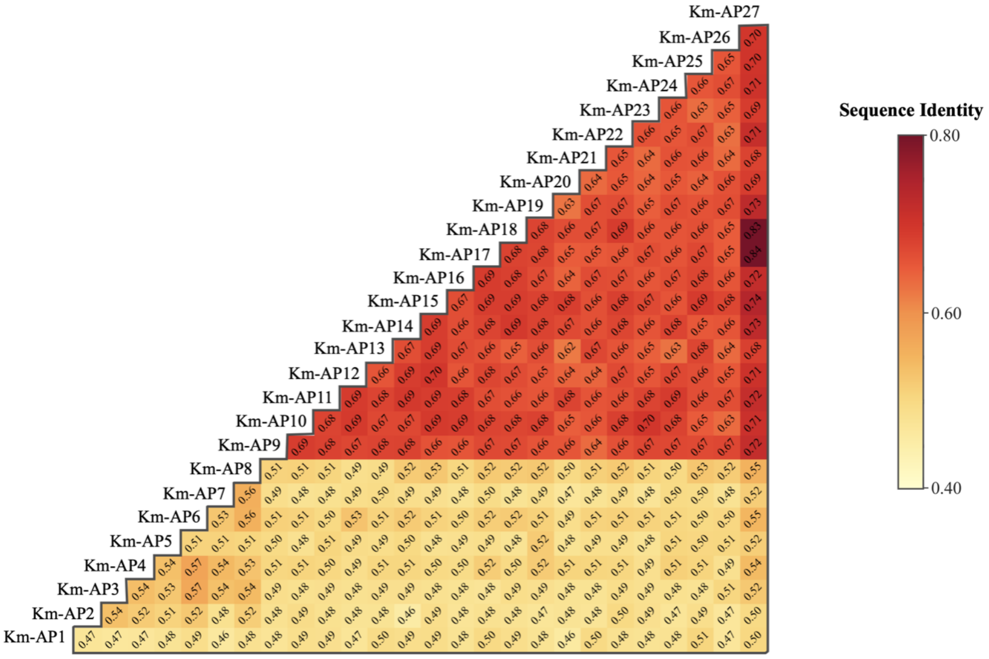

Fig. S47: Pairwise sequence identities of 27 Km-APs.

Table S5: Sequence specifications of Km-APs (1-3).

**AP**	**Sequence**
Km-AP1	M Q P H V S R M R A K E K A D E I E V Y V L T F P R R I D I D I R E G G T<br>D I M Q E W Q R A N G L P M A Y A N Y T V I S F V P I K H L S G Y T P E<br>K T E K R K L N V N S P F E R A L L E R L L Y N A L I Y C A Q K E L I W K<br>K L G K S L W E N E T Q Q I D K V C I H K A V E L K V E I L N D K I I I G<br>W D M V F S F S F I L T L Q D M I E E G E T I R P G M K V L F S D S R V R<br>R V Y T I L E V A P F G V L D R C P L Y K C S V Y D Y Y N E R G A Q Y M<br>L R L L T R S T R V I Y C R S H K E R L L Y A A L L L R P L C T L D T L L<br>P D T T V D L G K L I K L S A D E R L K Y L L R T L N L L R A Q F R Y L T<br>F A P D P F T I T K L G Y K M E N L A V P K I Q A H K D F A T V A S A L L<br>S N K L Y K G G N I K I A I L W D T N L F L K H D I S F H D L F A F V A L L<br>Q E L S L D L G I N V T I S P A T K A I A G W I N S M F F N H I D E R V L<br>A L E S L V A K T T V I A V I G S S I V A D E R T R F Y Q L L K E E T G G<br>L A D I A T Q I V T E K T I T A F K K I L E E N G L I D F K P T D S E D I L<br>K V I E C L K N S S F Y Y N I L N L I L G A A V G A G L S P Y T L A N N L<br>H A D L F I G L D V K F A D G V V G N G M M Q I I G S Q G H L I H R G M<br>G T G Y M P G E K I Q S S T L A S L I E E M I K A Y H D E F D R Y P K H I<br>V I H R D G F C R E D V S L V E K I C A Q Y Q I T F D L V Q I I K F P M L<br>K I C Y F D A T D N N F K T K K G L V L K N G N T A F L A S I Q P E E K<br>V G M A Q P I K I K E I T H T L P I S K V V E D I F C L S Y M N I H A L N K<br>T R L P W T I H Y A D N S S D A H Q Y G M V K M Y T G D K V A C P Y V
Km-AP2	M Q A Y V T R I I S K E R L N E L E V Y I M T F P R K I S V N I E D G V R<br>Q I M Q A W Q K A N A A P L A W K N Q I V I A F V P I R H L T G F T P Q<br>N V E E R K L D I D S P Y D R A L L E R L L R E S L V H T S E K N L Q M M<br>K M G K A I R R Q E V K K I R K I L I H R A V E I N V E I V D N D I C V G F<br>D I T Y K F S F V E T I Q D M L E Q G K P L R P G M R V V E S N S H V K R<br>V F E V I E V A P Y G V T D R C P I L N S S V W E Y F S Q R G A Q H I L R<br>L L T R A T K V V H V R T R H E R L S Y A A S L L K P L C T F D T M Q P<br>V E Q I E V S K I V R L S A N E R M R L M I R T L S E L K A Q Y R H L T Y<br>S P N P L S I S K C G F R I E E L S T P K I F M D R N Y A T V M A G I L N<br>L K M W K G G D I E I S I F W D E R F K E K H Q I S E H D V Y N F V S I L<br>E K L A K S H G I N L S V S T A F E A V D G F W T S E Y W E H I D E R I K<br>S L E P L V S Q C T V I A F I T N T I R D D Q F T R A Y Q L M K K Y F G G<br>K M N I S S Q V V H E K V I T A Y Q R I L D E H G L V N F R P N N L L Q V<br>E K A I N V I K N T S L Y R T I L N I M L G M Y V G A G I L A F T L S E E T<br>N A D L Y I G L D V I I V D G V N G T G M V N V V G S R G E L V H H A R<br>L N G H M P G E M V Q S D I L A S T L K K G V K A F E T R Y D K H P E H<br>V V V H R D G L M K E N L S L I K K I A A E F Q I T F D L V D V I K R P Q<br>H R I G F F D S V T N S F S T K K G T V Y K R G D E A Y L C A T N P E K<br>E V G M S Q P V R V E V V T R S L P I D E I V Q D I F S L S R L N I A S L L<br>K M R L P L T I H Y A D K S G L A H L R G M I N T Y S A N Q T G M P F V
Km-AP3	M E A Y I T E M R S R E K L D Q V E V Y S L T Y P R K F D N D V W L G N<br>K Q I M Q A M Q K A N G L P L A F L D D T I V S F S P V R Q L E G F T P L<br>N I E Q R K F N V D S P F E R A L L E R F V R E S L V F T G E R N L Q C I<br>K I G R A I M K Q Q V K Q I K E V I V Y K A V E I H V N I I D D N I I I G F<br>F I T F K F S F T E T L Q D L I Q E G K V V R P G L K V V F S D S K D R K<br>V F E L V D V A P Y G V S D R N P L F Q C S I Y D Y Y S E K G A Q W I L<br>R T L S R G T K V I Y V R T R Y Q E L E F A S E I L K P V C T F D T M Q P<br>V E N I E V S R L V R L S S N E R M R T S I K T I D Q L Y A Q F R H L T F A<br>P D P F T I S E C G F R I E Q L S Q P K F H F D K N F S T T T S G I L S C K<br>V Y R G G D V K I A I L F D Q R F Y E E L D I T D H D V Y D F L S I L K K<br>L A I N K G I T C T L S S S W K A I S G K F T L D Y F R Q V T N M V K S L<br>E P L Y S E T V V L A F V T N T L M N N E Y T R Y Y E K M K Q E M G G<br>E M N I S L E I V N E K V I T A Y K R L L Q Q H G L I N L K P F N L E Q I E<br>R L V E L I R N T S F Y K T I L N L L L G L Y I K S G I L P Y L L A E D L H<br>A D V F V G L D V V I I S G D V G S G M I S V V G S Q G E L V K R S I G V<br>G F L P G E I I Q S S I L S D T [illegible] K E V L K A Y Q D R F Q K Y P E H V V I<br>H R D G F M R E N I S L I H K I M S E Y Q I T F D V V E I T K K P Q R R F<br>A L Y N S V D N T F S T E K G L V F R R G N T A Y L I A T E P L K K V G<br>M A L P V R L K Q V T K T L P F D Q V I Q D I F D L S F L N H A A L N K<br>M R L P I T I N Y A D L S G L G Y L R G E I N S Y S G E M T A M P Y V

Table S6: Sequence specifications of Km-APs (4-6).

**Protein**	**Sequence**
Km-AP4	M E M Y V T E M T A R Q K L E E L M V Y V Y V F P R R E T H N V W I G<br>T H L I M Q A W Q K S N G Q P L A F L H Q T I L A F S P I R H W T G Y T P<br>E K T Q K R K I N I T S P F D R S L L E R L L K N A M I F T A E K N L K W<br>R R L G R S L M K I E V Q Q I K R I I I Y K C I K I H V E V I D N R I V V G F<br>H L T H E F E F T E T L Q D M I Q K G K V V R P G L K V V L S D S K V K<br>K V Y E L V E V A P F G V F D R D P L L Q C S I Y Q F F V Q R G A Q H I L<br>R T L T R G T K C V E V R S Y Y E E L L F P A C L L R P L C T I D T L E P<br>V D V V E L G R E V R L T A D K R M R D M L R T M T Q L R A Q Y R Y L<br>T F A P Q P Y S I S E M G F K L E T L A S P R V H F D K N Y A T T T A G L<br>E T S K C Y K G G N I E V A V L L D T R F R E K L E I T D H D V Y N F V S<br>I L E R L A K N L G V E I T V S T S T Q A V R G R F T T D F Y Q Q I T H E I<br>L S L E S L F S Q C T I L A F V S S S M I S N Q Y T R T Y D K M K Q E F G G<br>E W N I A A Q I I N E R T I S A Y Q K L L D Q N G L T D L R P W N K L E V<br>L K L I N V I K N T S F Y R T V Q N I L L G L Y V K A G I Q P W T L A N N<br>T N S D L Y V G I D V L W I C G D V G A G M M S L V G S K G E L V Q R S<br>R I S G F L P G E M I N A D T L S D T L E R L L K A F E T R F K R Y P E H<br>I T V H R D G K M R E D I S L V K K I M A K Y Q I T Y D F V D V I K K P N<br>R R L A F F D A V N N T Y S T K Q G T V Y R R G N T A F V V T T D P L E<br>E V G M A R P V K I K Q I T K S L P I E E I V Q D I Y N L S T L H I A A V E<br>K M R L P L S I N F A D E A G N A Y L R G M V N E Y T A F K T S L P F V
Km-AP5	M K G F V S E M V A R Q K A N Q I E V Y A L D F P R K I D I D I W N G T<br>K L I M E A M E K A N N Q P L A F V E Q T I I T F V P I R Q Y N G F V P E<br>E V Q Q R H F D I T S P F E R G L L E R L I K E A L V F T A E R H L E M M<br>R I G K S L Q K Q Q T K Q L N E V I V Y K A I E L H V E I V D N R I N V G<br>Y F I T Y E F S Y V Y T L L D M L E E G K V L R P G M R V I I S D S F V R<br>R T Y V V S E V A P Y G V S D R C P L F N V S V Y N F F V K K G A Q H I L<br>R T L T R S T R V V H V R S R F Q E M E Y G A C I L R P V C T L E T M L<br>P T D Q E D I S R H I R L S S D E K M K R G I R I I S E L Y A E F R H L S Y<br>S P E P W T I A Q C G F K I Q E L S A P K V H F E R N F A T T S S G V M S<br>G K L Y R G G D I E I A V L L D Q R L R L K H Q V T L K D I W N F V S I L<br>K K L A K S Q G V N V T V S T A T Q A V A G K V T T N F F Q H L S E K V<br>E S L E P L V S E C V V L A I V T S T F V S N E K T R F Y Q L M K K V F G<br>G E W D I S I Q I V T D R V I I A Y M K I L D Q N G M I D F K P D D K E Q<br>C N K C I E L I R D T S F F Y T L M N I L L G M L V G A G I L C W I L A D T<br>L H S D M Y V G L D V L H V N G D I G G G F M S L V G S K G H L V H K A<br>R L P G F Q P G E K I E A D T L S D M I E K S L R A F H S R F N R L P E H<br>I V V H R D G K M R E N V S L V K K I A S E Y E I T F D I V E V T K R P R<br>R R F A Y F D A V D N T F S C Q Q G T V F K A G N T A F L I A T E P L E<br>E V G M S Q P I K I K Q V T R S L P I S E I I K D A F N L S Y Q H I A A L Q<br>K A R L P V S I H S A D L A G V G Y S R G Q V N P Y S G D K T N M P F V
Km-AP6	M K A Y I T E M V A K E R L N E L K V Y V L D Y P R K H D I L N Y I D E<br>E E I R E A W Q R S N G Q P L A F N E R T I L A F T P V K H F Q G W T P<br>L N V E Q R H I N V D S P F D R S L M E R L V K N S I V Y T G Q K N L H Y<br>Q K V G K A M K R L L V K Q I K K I L I H E A I E I H V R V I E N E I I I G<br>S D L T H K F R F S Y T I L D M L E E G K T V R P G L R V V L A D S K D<br>Q R V Y E L V H V A P Y G V S D R C P L F N C S I Y D F Y S K H G A Q F<br>Q L R T L T R A T R C V Y V R S R F Q E L E F A S T L L R P L C T M D T<br>L Q P E E V I E V S K E T K L T A S K R M R L Q L R L I D Q L Y A Q F R Q<br>Y T F A P D P F T I S E C G F K L L E L S T P K F H F H K D Y A T I T A G<br>I L T A K V Y K G G E V K I S V L Y D T R L R L K L Q I S D K D I Y S F L<br>A I L E R L A K L H G F T I S V S S A W K A V A G K F T T T Y W E Q I T L<br>L I H S L E S L V S Q T V I L A F V S S E M I A D L F T R F Y E L L K E E F<br>G G K Y D I S A Q I V T E K V L T A F Q R I L E Q N G L I N L R P F N Q E<br>Q V N R A I N L I R N S S L Y Y T I L N I M L G M F V K S G I L P Y I L A E<br>N L H S D L F V G L D I I H I S G V V G V G M M S I I G S K G E L I Q R S P<br>V S G Y M P G E M I N S S T L S S T L E K S L K A F E D K Y N K Y P K H I<br>V V H R D G K W R E N L A L V E K I M A K Y E I T Y D I V N I I K F P N H<br>R L A Y Y D S V N N T Y S T K Q G T V Y R K G N T A F V I S T D P L K E<br>V G M S Q P V K I E Q V T H T L P I E Q V I K D I Y D L S Y L N I A A L N<br>K M R L P V S I H S A D N A G N A Y S R G E I N T R S G E K T S M P Y V

Table S7: Sequence specifications of Km-APs (7-9).

Protein	Sequence
Km-AP7	M K A W I T E R V A K E K A N Q L E I Y S Y T Y P R K E A D N V Y V G N<br>K L I M Q A L M K S N G K P L A Y L E N T I I S F S P V K Q R N G F T P L<br>N T E K R H F N I T S P F E R S L F E K F I K N S L V Y S A Q R H L E W R<br>K I G R A I Q E L E T Q Q I Q Q V N I F E A I R V N V E I V D D K I C V G F<br>H L I H K F S Y T K T L L D M I K Q G K V V R P G M K V V F S D S K D R<br>K T Y E V V E V A P Y G T T D R N P L L Q S S I W D Y F V N K G A Q H L<br>L R T L S R S S K V V E V R S Y F Q K L M F A A S I L K P V C T L E T L E<br>S D E V I D I G R H T K L S A S Q R L K L Q L K I I S Q L K A Q F R R L T F<br>S P N P W T I S R M G Y K K Q T L S S P R F K F D K D F A T T V S G I R T<br>C K V Y K G G N V K I A V F F D M S F Y E K L Q I T E K D V E N F I A I L<br>E H L A E D Q G I E I S V S P S S E A V V G R W T T N T Y E H I D H R S N<br>S L E P L F S E T T V I A F V S T S L M D N L Y T R Y Y Q K C K K V C G<br>G K A D I A S Q I I T E R T L T A Y K R L L Q Q N G M I D F K P E N K Q E<br>V L K C I N I L R D S S F Y Y T I M N Q M L G L Y V K A G I I P W V L S N<br>N T N A D V Y V G I D L L I I S G V V G S G E M S V V G N R G E L V K H S<br>M I T G F L P G E Q I K S T T L A S M L K K A I K A F E T K F Q R Y P E H<br>V T I H R D G K W R E N V A L V E K L M S K F E I S Y D V V D V V K F P<br>N L R F A Y H D S V D N T W S C Q K G T V Y K R G N E A Y L I S T N P D<br>E K V G M S I P I K I E Q I T H S L P F E E V I Q D I Y C L S Y L N I A S L N<br>R C R L P V T I H F A D M A S V A Y N R G Q I T S F S G D L T S L P Y V
Km-AP8	M K A W I T E R V A K E K A N Q L E I Y S Y T Y P R K E A D N V Y V G N<br>K L I M Q A L M K S N G K P L A Y L E N T I I S F S P V K Q R N G F T P L<br>N T E K R H F N I T S P F E R S L F E K F I K N S L V Y S A Q R H L E W R<br>K I G R A I Q E L E T Q Q I Q Q V N I F E A I R V N V E I V D D K I C V G F<br>H L I H K F S Y T K T L L D M I K Q G K V V R P G M K V V F S D S K D R<br>K T Y E V V E V A P Y G T T D R N P L L Q S S I W D Y F V N K G A Q H L<br>L R T L S R S S K V V E V R S Y F Q K L M F A A S I L K P V C T L E T L E<br>S D E V I D I G R H T K L S A S Q R L K L Q L K I I S Q L K A Q F R R L T F<br>S P N P W T I S R M G Y K K Q T L S S P R F K F D K D F A T T V S G I R T<br>C K V Y K G G N V K I A V F F D M S F Y E K L Q I T E K D V E N F I A I L<br>E H L A E D Q G I E I S V S P S S E A V V G R W T T N T Y E H I D H R S N<br>S L E P L F S E T T V I A F V S T S L M D N L Y T R Y Y Q K C K K V C G<br>G K A D I A S Q I I T E R T L T A Y K R L L Q Q N G M I D F K P E N K Q E<br>V L K C I N I L R D S S F Y Y T I M N Q M L G L Y V K A G I I P W V L S N<br>N T N A D V Y V G I D L L I I S G V V G S G E M S V V G N R G E L V K H S<br>M I T G F L P G E Q I K S T T L A S M L K K A I K A F E T K F Q R Y P E H<br>V T I H R D G K W R E N V A L V E K L M S K F E I S Y D V V D V V K F P<br>N L R F A Y H D S V D N T W S C Q K G T V Y K R G N E A Y L I S T N P D<br>E K V G M S I P I K I E Q I T H S L P F E E V I Q D I Y C L S Y L N I A S L N<br>R C R L P V T I H F A D M A S V A Y N R G Q I T S F S G D L T S L P Y V
Km-AP9	M Y G F I T E I V A R E R A E E L K V Y Y V L V F P R F L S C T V F I S V K<br>E I M L G W Q R A N G L P L A F V E I T I V A F S P V R H M V G Y T P K<br>N V E E R K I N V T S P F E R A T L E R L L K N S L I F T G E K N L E L K<br>Q V G H A L E S H Q V K E I E R I I I H E A V E I T V N I V E D R I I L G F<br>D L T Y Q Y K Y T Y T I Q D M I E Q G K P L R P G E R V I I S N S K V K K<br>V Y D V V D L A T Y G V F D R C P L L Q T S V Y Q Y F C E K G A Q H V L<br>R L L T R S T R V I E V R T K F E E L V Y A A T L L R P L C T L E T M Q P<br>N E V I N L A R L I K L S A S K R M R T S E R F I S Q V W A N Y K H F T Y<br>A P N P F T I A R Q G Y K L R E L S T P K V Q F D K T Y A T K V S G V K<br>T G K L Y K G G N I K I A L L L D T D F K L K H N V T E E D I F A Y I A L<br>L E Q I S I A H G V T V T I S D A I K A V N G R F T L D F F E H I A E K I K<br>S L K P L F D Q T T V L A F I S G E Y E S N E Y T R F Y Q L L K Q V F G G<br>K D D I A S Q V I T E K V I Q A F Q K L L E K H G L L D F K P N D D Q Q I<br>N K V V N I L K D D E F F Y V V E N I L L G V Y V K S G I Q P W I L A N T<br>T H S D L F V G L D V V K V D G K V G H G M A S V V G S Q G E L I K R Q<br>P D P S F E P G E K I R D D L L A S R L K Q T I R S Y H S R F Q E F P E H I<br>T I H R D G F W R E E T S L L E K I M S H Y E I T Y D I V E V I K K P N R<br>R L A L Y D S T T D T F S T Q Q G L V Y K R G D E A Y L C A T D P E Q K<br>V G M A Q P I K I H Q N T R T L P F E M V I E D V Y C L S K L H I A S M N<br>K M R L P V T V E S A D L A A T A F E R G Q I M P K S G N R T S L P F V

Table S8: Sequence specifications of Km-APs (10-12).

**Protein**	**Sequence**
Km-AP10	M Q A Y I T E I V S R E R A N E L K V Y Y V L H F P R Y L S T T V F L G I K<br>E I M E A W Q R A N G L P L A Y V D I T I M A F S P V R H L C G Y T P E<br>R V E E R H L N V D S P Y E R A L L E R L L K N A L I F T A E K H L E A E<br>Q S G K S L Q S H Q V L E I E Y I I I Y Q A I E L T V N I D D D R I K I G F D<br>L T H Q F E Y T H T L Q D M I E Q G K S V R P G M R V V I A N G E V L K<br>A Y E V V E L A P Y G V H D R C P L L Q T S I Y Q Y Y V E K G A Q H V L<br>R L F T R G T R L I E V R S K F E R L V F A A T L L K P V A T L E T M Q P<br>N Q Q Q N L S K I I K L S A D E R M R T T L R L I S E L E A Q Y K H F T Y<br>A P N P L T I A R N G Y K L R E L A T P K V K F E R D Y A T V V S G I Q<br>N G K L Y K G G N V K V S I L I D S E M K E K H N I T D E D I Y T F I A L<br>L E Q M A K A Q G I N V S I S T T W K A V S G Y F T Q D F F E Q I A E R I<br>L S L Q P L I A Q T T V I A F I P S A L E S D E Y T R W Y E I L K Q Q F G G<br>K W D I S A Q V I T E R V I Q A F Q R I L E L H G L L N F K P N D D E Q V<br>D R V I D V L K D E E F Y Y T V V N I L L G I Y V K A G I E A W I L A N T<br>M H S D F F I G L D V V K K N G N H G N G M A Q V I G S Q G H L I K R Q<br>R D P S F E P G E K I L A S L L A T M I K R V I K A Y E S L F N E K P E H I<br>T I H R D G F W R E D I S L I E K V M A H W E I T Y D I V E V I K K P N R<br>R L A F Y D S T T N T W S T R Q G T I Y K R G D T A Y L C S T N P E Q R<br>I G M A Q P I K L F Q I T K T L P F E H V V E D V Y N L S F L H D H S V N<br>K M R L P A T I H S A D L F A N A Y E R G E V M S R S G N R V S L P F V
Km-AP11	M Q P F I T E I V S R T K A D E L K V Y Y V L V F P R K E S D T V K L G V K<br>E I M Q A W R R A N D S P L A Y V E E T I V A F S P V R H M V G Y T P L<br>K V E E R K L N V T S P F E R A L L E R L I K N A L I F T A E R H L Q A Q<br>Q V G H A A Q S Q K T L E I E Q V L I Y K A I E F E V R I V N D R V L L G<br>F D L T Y Q F D Y T Y T I Q S M I E K G K P V R P G M R V V H A N S F V<br>K K T Y D L V H V A P Y G V F D R C P L F Q T S L Y Q F Y V E K G A E F<br>L L R L F T R S T R V I Q V R S L F E M L I Y A D T L L K P L C T L Q T M<br>E P N Q V E N V A R S I R L S A S K R M K A Q L R L L S E V Y A E Y K H F<br>T Y A P N P F S I A R Q G Y K L E E L S T P K V K F E K D F A T I V S A V<br>K T G K L Y K G G E I R I A I L L D T N M K L R H Q I T D E D I Y A F I S L<br>L K K M A E A H G I D C T I S T A I K A V A G R F T I N F F E N I A E R I E<br>S L K P L I A Q T T V L A V L P G A L E S D E K T R L Y E L K K Q F F G G<br>K W D I A S Q V I T E K V L E A F R R I K D K H G L E D F K P N D N D Q<br>V E R A I N V L K N D E F F K T V Y N I L L G I Y V K A G I Q P W I L A N<br>T L H A D F F I G L D V V L V N G N T G P G M S T V V G S Q G Q L I L R<br>V R G P G H L P G E K I R D D L L A N Q I E Q I L K S Y E S I F D R Y P E<br>H I T I H R D G F W R E D I S L L E K I F S H F E I T Y D I V D V I K K P N<br>R R L A F Y D S T T N T F S T R Q G T V Y E R G N E A F L C S T Q P Q Q<br>K V G M A Q P V K L H Q N T K T L P F E H I I Q D I Y N L S K L H F H A L<br>N K T R L P A T I H Y A D L S A T A Y Q R G Q I M S Y S G N R T S L P F V
Km-AP12	M E V Y I T E I V A R T K A N E L K V Y E F H F P K Y L S K V V F D G V<br>K E I M Y A L Q R A N G S P L A Y N E E T I V A F S P V R H M V G W T P<br>L R V E E R H I D V N S P F E R A L L E R L L K N S L I F T G E R H L H A<br>K Q L G K A A Q L T K T L E I E E V L I Y E A V E L L V N I V N D E I I L G<br>F D L T H R F K Y T E T V Q D M I E Q G K S V R P G L R V I I Q N S Y V E<br>K T Y D V V E V A P Y N V F D R C P I L Q T S I Y Q F Y V D K G A E F I<br>L R L L T R A T R V V Q V R S K F Q R L E Y A A T L L R P L C T L D T M<br>Q P N S V E N V S R V I K L S A S E R M K L T L R I L S E L E A Q Y R H F<br>T F A P N P F T I A R Q G Y K L K E L A V P K V Q F D R D Y A T V V S G<br>V K T G K L Y K G G N I R I S L L L D S E L K L R L E I T E E D L Y T F I S<br>L L E Q L A E N Q G V N I T I S T A T K A V S G Y F T Q T F F Q N I A E L<br>I E A L K P L F S Q T V V L A F L P A A L E A D K K T R F Y E I K K Q Q F<br>G G K D D I P T Q V I T E K V L E A F R K L L Q Q F G L I N F R P N D D N<br>Q C N K A L D I L K D S E F F Y T V L N I L L G V Y V K A G I I A W I L A<br>N D A H A D L F I G L D V S K K N G N T G H G M A S V I G S Q G Q L I N R<br>V R Y S G Y L P G E E I R D D [illegible] L A N H L K Q T I K A Y E S L F D E F P E<br>H I T I F R D G F S R E E I A L I E K V M S H Y E I T Y D I V N V I K K P N<br>R R L A Y Y N S T D N T W S T R Q G T I Y K R G D E A F L C A T D P L Q<br>K V G M A Q P I K I H Q V T R T L P F E H V V K D V F D L S F L H N H S L<br>N K M R L P A T I H T A D L L A N A Y E R G E I M S K S G N R V S L P F V

Table S9: Sequence specifications of Km-APs (13-15).

**Protein**	**Sequence**
Km-AP13	M Y P F I T E L Q A K E K A N E I E V Y V F H F P R F L S D N V F L A V<br>E D I E Q A W R R A N A Q P L A Y V E E T I V A F T P V R H M V G Y T<br>P E K V E E R H L D V D S P Y E R A L L E R L I K N S L I F T A E R N L E<br>A K K S G K A L E S N L T Q E I K R T I I H E S V E F T V N I I N D Q I I L<br>G F D L T Y Q F E Y T Y T L Q D M I E Q G K P V R P G M K V V I Q D G<br>K V F Y E Y E V V E V A P Y G V D D R C P L F Q T S L R Q F Y V E K G<br>A E F L L R L F T R S T R V V Q V R S R F E H L I W A A T L L K P L C T<br>F E T M K P N Q V I N L S K V I R L S A S Q R M R T S L R F L S Q V Y A<br>Q Y K H L T F A P N P F T I A R Q G Y K R K E L A T P K V K F D R D Y<br>A T I V S G V K N S K L Y K G G N I K V S V L L D T D F K Q E F H I T E<br>E D I F Q F I A L L Q K V A E A Q G I N C T I S T S W K A V A G R W T Q<br>D F F E H V A L K I E A L K P L F A K T T V L A F I P G A L K S D E K T<br>R W Y E L L K Q K F G G K W D I S T Q V I T E K T I Q A F K K L L N K<br>N G L D D F K P N D D E Q V N R V V D V L K D D S F Y Y T V L N I L L<br>G V Y C K A G I E P W V L A N T T H S D L F I G L D V V N K N G V V G<br>T G M M V L V G S Q G F L V K Y V R G P I F E P G E Q I Q D D L L A N<br>T L E Q V L K S Y H S H F Q E Y P K H I T V F R D G K F R E N T S L L E<br>K I M A H Y E I T Y D I V N I V K K P N R R L A Y Y D S V T N T W S T K<br>R G T V Y K K G N E A L L C T T N P E Q K V G M A Q P I K I H Q N T R<br>T L P F T H V V E D V Y N L S F L H H H A V L K M R L P L T I H Y A D L<br>I A T G Y E R G Q I M P R S G N R T S L P F V
Km-AP14	M E A Y I T E I Q A R T K A N D I K V Y T L T F P R K L S K T T I I G V K<br>Q I M E G L E R A N G S P L A Y V E E T I V A F T P I R Q M V G Y T P E N<br>V E E R K I N T T S P F E R S L L E R L V K N S L I F T A E R H L Q A Q Q<br>I G K S A R L T Q V L Q I E R I L I H K A F Q L E V N I D N D R I I L G F D<br>L T H K F E Y T Y T L Q S M I E Q G K S V R P G M R V I I A N S K T R K<br>T Y T V V H V A P F N V F D R C P L F Q T S I Y Q F Y V K K G A Q Y V L<br>R L F T R S T R V I Q V R S F H E K L I F A A T L L R P L A T K E T M Q P<br>N Q V I N I S K A I K L S A S Q R M K L T L R L I T Q V Y A H Y R H F T Y<br>A P D P F T I S R N G Y K L E D L A T P K V L F Q K D Y A T K V S G V K<br>T G K L Y K G G N I R I S L L L D S N L K L K F E I T E E D L Y A F I A L L<br>E K I A E N Q G I N V T I S T T W K A V A G R F T K D F F E H I A E Q I E<br>S L K P L I A Q T T V L A F I S S S L L S D E K T R F Y E V L K Q F F G G L<br>W D I H A Q V I T E K T L E S W Q K L L N K H G L I D L K P N D N I Q C N<br>R A I N I L K N D E F F Y T V I N I L L G V Y T K S G I E P W V L S N T L H<br>S D F F V G L D V V Q K N G V N G T G M S S V V G S Q G R L I L R V R G<br>P G F L P G E E I R D S L L A T M I K R V I K S Y H S L F K E F P K H I T I<br>H R D G K W R E E E A L I E K I A A H Y Q V T Y D I V E V I K K P N R R<br>L A Y Y D S V T N T F S T Q Q G L V Y K K G D E A F L C T T D P E E K V<br>G M A Q P I K L H Q V T K T L P F D H V V E D V Y S L S K L H I H S M N<br>K S R L P A T I D Y A D L A A T A Y Q R G E I M P Y S G D R T N L P F V
Km-AP15	M F P Y I T E I I A R S K A D E I K V Y V F I F P R K L S T T V Y L S V K E<br>I M Y S W Q R A D G S P L A Y V E I T I V A F T P I R H L V G Y T P E R V<br>E E R H L D T D S P Y E R A L L E R L V K N S L I F T A E K H L H A Q Q S<br>G K A L Q L V Q T L E I E R I L I Y E A V Q F D V N I D N D R I L M G F D<br>L T H R F D Y S Y T L Q D M I E Q G K T L R P G M R V R I A N S E V F K<br>T Y E V V N V A P Y G V F D R C P L L Q T S I Y Q F Y N E K G A E Y I L<br>R L F T R A T K V I Q V R S K F Q K L I F A A T L L K P L C T F E T M Q P<br>N D Q Q N V A R A I K L S A S E R M Q T T L R L L S E V Y A Q Y R H L T<br>F A P N P F T I A R Q G Y K L K Q L A T P K V K F E R D Y A T I V S G I K<br>T S K L Y K G G N I R I S L Q L D S D F Y Q K H H I T D E D I Y T F I S L L<br>K K I A E A Q G I D V T I S D A T K A V L G R F T L N F F E H I S L K I K<br>A L E S L F A K T T V L A F I S G S L K S D E K T R F Y E I L K Q Q F G G<br>K D D I S S Q V I S E Q T F E A F Q K I L Q Q H G L I D F K P N D N Q E V<br>Q K A I D V L K N D S F Y Y T V L N I L L G I Y T K A G I E P W I L A N P<br>T H A D L F I G L D V V K K N G L N G N G M S T V V G S Q G H L I R R A<br>R D S G Y L P G E K L R P D L L A T L I E K T I K S Y E S K Y N R F P K H<br>I T I H R D G F W R D E I S L I E K I M A H Y E I T Y D L V E I I K K P N R<br>R L A F Y N S T T N T W S T R K G L V Y K R G N E A Y L C A T D P L Q K<br>V G M A N P I K I R Q V T K T L P F E Q I V E D V Y Q L S F L H I H S L N<br>K M R L P L T I H T A D K L A T G Y N K G Q I H P R S G H R T S L P F V

Table S10: Sequence specifications of Km-APs (16-18).

**Protein**	**Sequence**
Km-AP16	M H P Y I T E I Q S R N K A D E L E V Y T L H F P R K L S T N T Y L S I K<br>K I A F G W Q R A N G S P L A Y V E E T I V A F S P V R Q Y V G Y T P L<br>R I E E R K L N V T S P F E R A L L E R L I K N A L I F T A E R H L E A K R<br>I G H A L Q S H K V K E L E E V L I H R A I E L T V N V D D D R I L V G F<br>D Y T Y Q F E Y S Y T I Q S M I E Q G K P I R P G E K V V H A N S R V K Y<br>V Y E V V H I A P Y G V F D R C P I L Q T S I Y Q H Y V E K G A Q F V L R<br>T F T R A T R V V Q V R S F F E R L S Y A A T L L K P L C S L E T M Q P<br>V S V L N V G K L I R L S A S K R M K L T L R I L T Q V Y A Q Y K H L T Y<br>A P D P F T I A R Q G Y K R K E L S T P K V K F D Y D Y A T V D S G I K<br>N S K L Y K G G N I R V S L L L D D D L K L K L E I T D E D I Y A F I A L L<br>K K L A E A Q G I T I T I S D A W K A A N G R W T Q D F F E H V A E K I<br>E S L R P L F A Q T T V L A F I S A E L E S D H K T R F Y E L L K K F F G<br>G L D D I S A Q V I T E K T L Q A Y Q R V L N K N G L T D F K P N D N E<br>E C D R V I D I L K D D E L Y Y T V Y N I L L G I Y V K S G I S A W I L A D<br>T T H A D L F I G L D V V N V N G K V G N G M S T L I G C Q G Q L I K R<br>V K Y N S F L P G E K I L P D L L S D Q I K R V I K A Y E S L Y D E K P E<br>H I T I H R D G F N R E D T S L V Y K V L A H W E I T Y D I V E I I K K P N<br>R R M A F H N S D T N T W S T R Q G L I L K R G N E A F V C S T N P E E<br>K V G M A Q P I R I F Q V T K T L P F E H I I E D V Y K L S F L H I H S L N<br>K M R L P A T I N Y A D L I A N A Y Q R G Q V M P R S G N R T S L P F V
Km-AP17	M E P F I T E I V A R Q K A N E L L V Y Y V L H F P R K L V T T T Y D G V<br>K D I K K G W Q R A N E Q P L A Y A E E T I V A F S P I R H M V G Y T P<br>Q R E E E R K I D V T S P Y E R A L L Q R L L K N A L I F T A Q K H L Q A<br>Q K I G H A L Q S H Q V Q E I E R I L I Y K A V E L D V N I V N D R I V L G<br>F H L N Y Q F D Y T L T L Q D M I E Q G K P I R P G M K V I E S D G E T<br>F Y T Y E V V H V A P Y N V F D R C P I L Q T S I Y Q Y Y V E K G A Q F I<br>L R L L T R S T R V I E V R S F F E K L I K A A T L L R P L C T L E T M Q<br>P Q Q V L N L S R L I K L S A S K R M K D T L R L L S Q V Y A Q Y R H V<br>T F A P N P L T I A R L G Y K L K D L A T P K V H F D R D Y A T V L S G<br>I K S A K V Y K G G N I K V S L L I D S E L K L K M N I T E K D I Y Q F I A<br>L L E S L A E A Q G V N I T I S S S W K A V S G Y F T L T F F E H I A E K I<br>K S L K P L F A Q T V V L A F I S G S L L S D H K T R L Y E L L K E Y F G<br>G K W D I P S Q V I T E R T I E A F Q R L L I K H G L I D F K P D D Q D Q<br>I N K A I N T L K D S S L Y Y L V V N I L L G I Y V K S G I Q P W I L A N T<br>T H A D F F V G L D I V K E N G L T G P G M S T V V G K Q G Q L I Q Q Q<br>R G K S Y L P G E K I R A D L L A S L I E Q V I K A Y E S L Y K E H P H H<br>I T I H R D G F F R E E T S L M E K I M A H Y E I T Y D I V N I I K K P N R<br>R V A D Y D A V T N T F S T K Q G L I Y K R G D E A Y L C S T N P L E K<br>V G M A Q P V R L H Q V T K T L P F S E I I E D V Y Q L S F L H H H S V N<br>K M R L P A T I H T A D K L A N A Y N R G Q V M V Y S G N K T S L P F V
Km-AP18	M Y A Y I T E I V S K S K A D E L K V Y T L H F P K R L S T N T F L S V E<br>E M M F G W Q R A N G L P L A W A E I T I V A F T P V R Q L V G Y T P<br>L Q V E E R K L N V D S P F E R A L L E R L L K N S L I F T G Q R H L Q A<br>Q Q I G R S A R L T E V Q E I E E I L I H R A I E L E V E I I D N E I E I G F<br>D L T F Q F D Y T Y T L Q D M I E K G K P V R P G M R V V H A N S R T<br>E K C Y E V I N V A P Y G V F D R C P L L Q T S I Y Q Y Y V K K G S Q Y I<br>L R T F T R S T R V V E V R S K F Q E L I F A A T L L K P L C T K E T L Q<br>P Q Q V L N V S R H I K L S A D E R M K T T L R L L T Q V Y A Q Y R H F<br>T F A P D P L T I S R N G Y K L R Q L S T P K V H F D R D H A T V T S G<br>V K N G K L Y K G G N I K V S I L L D T D F K Q K H H I T D E D L Y Q F I<br>A V L K K I A I A Q G V T C T I S T A W K A V V G R Y T L D F F E Q I A E<br>K I E S L K P L F A Q T T V L A F I S A S L L S D E F T R F Y E L Q K E V F<br>G G K D D I P S Q V I T E R T L E A F Q K L L D K H G L V D F K P D D Q I<br>Q I N K A I D I L K N D S F Y Y T V L N I L L G I Y V K A G I Q P W I L A N<br>T A H S D C F I G L D L V E K N G V Y G H G M S T V I G S Q G Q L I K R<br>Q P G P G Y Q P G E K V R P [illegible] L L S N H I K Q T I K S Y H S L F K E F P H<br>H I T I H R D G F A R E E I S I M E K I C A H Y H I T Y D I I E V I K R P N<br>R R V A F Y N T T T N T F S T Q Q G L V F K R G N E A F L C S T N P E Q<br>K I G M A L P I K I E Q V T R T L P F E H I V E D V Y C L S K L H I H S L N<br>K M R Q P L T I D T A D L I A N A Y E R G E I M P R S G N R V N V P F V

Table S11: Sequence specifications of Km-APs (19-21).

**Protein**	**Sequence**
Km-AP19	M K P Y I T E R V A R T R A E Q I K V Y I L T F P R F L S K V V R L S I K
	K I A Y G W Q R A N A Q P L A Y A E D T I V A F S P V R H M V G Y T P L
	K V E E R H L D V T S P F E R S L L E R L I K N A L I F T A E R H L Q L Q
	K I G K S L Q S L E V Q E I E Q I L I Y K A V E L E V E I I D N R I L I G F H
	L T H R F E Y S Y T L Q D M I E K G K S I R P G M R V I Y S N S E V E K V
	Y T V V N V A P Y N V H D R C P L F Q T S I Y Q Y Y V E K G A E F I L R L
	F T R S T R V I Q V R S F F Q K L I Y A S V L L R P L C T K E T M Q P T Q
	V I N V A R L I K M S S D Q R M R T T W R F M S E L Y A E Y R H L T Y A
	P D P F T I A R Q G Y K L K D L S E P K M H F D R D Y A T I T S G I L T G
	K L Y K G G E I K V S L L I D T D L K L K F N I T N E D V Y T F I A L L E
	K I A H A Q G V T V T I S T A W K A V V G R F T K D F F E H I A H L I E S
	L R P L F A Q T V V L A F L S G A L L S D E Y I R A Y D L L K Q Q F G G R
	W D I N T Q V I T E R T I Q S F Q R V L D K H G L L D F K P N D D E Q V
	N K A L D V L K D D S L Y Y T V I N I L L G I Y T K A G I Q P W V L A N T
	L H S D M F I G L D V V T K N G N F G G G M M S V V G S Q G H L V K R V
	R D P S F E P G E K L F S D L L S T M L K K T I K S Y E T L F R R F P E H
	I T I H R D G F F R E D T S L I E K I C A H W E I T Y D I V N I I K K P N R
	R I A F F N S V T N T W S T Q Q G T V Y K R G N E A F L I A T D P K E K I
	G M A Q P I K I E Q V T K T L P F E K I V E D V Y Q L S F L H I H S M Q K
	M R L P A T I Q Y A D K A A N A Y E R G Q V M A R S G N R T N L P F V
Km-AP20	M F A F I T E I K A R T R A D E L L V Y Y V F T F P R Y L S N T V F L G V K
	E I M F G L Q R A N N S P L A Y V E E T I V S F T P V R Q M V G Y T P K
	K V Q K R H I N V D S P Y E R S L L E R L L R N S M V F T G E R H L H A
	Q Q I G E A L R L H K V K Q I E N I I I Y R A I Q F E V R I D N N R I V L G
	F D Y T F Q F D Y T Y T L Q S M L E K G K S V R P G M R V I I S D S K V
	F R E Y T V V N V A T F G V F D R C P L L Q T S I Y Q Y Y V Q K G S Q H
	I L R L L T R S T K L I Q V R T R F E H L V F A S S L L K P L C T K E T L E
	P V Q Q L N L A R V I K L S A S E R M K D Q L R F V T E L R A Q Y K H L
	T Y A P D P F S I A R Q G Y K R L Q L S T P K V F F D R D Y A T V V S A I
	K T S K F Y K G G N I R V A L L L D S N F K L K H E I T D E D I Y A L I S L
	L K K I A T S Q G V D V T I S D A W K A V V G R F T L D F F E N I D E K I
	E S L K P L F A Q T T V L A F I S S S E E S N H R T R F Y E I K K R L F G G
	K W D I G S Q V I T E K F L E A Y Q K I L D K H G L I D F R P N D Q Q Q V
	Q R L V E V L K N D E F F Y T V V N I L L G I Y V K S G I V P W I L A N T
	T H A D F F I G L D V I E K N G N L G H G A I S V V G S Q G Q L I Q R A R
	G K G F E P G E K I R A D L L A S M M E R T I R S Y E S Y Y D E F P K H I
	T I Y R D G F W R E D T A L M E K I M A H F E I T Y D I V E I V K K P N R
	R I A F Y N S T T N T F S T Q Q G T I L K R G N E A Y L C S T N P L Q K V
	G M A R P V R L F Q E T R T L P F D H I V E D V Y Q L S K L H I H A L N
	K M R L P A T I H L A D K L A T A Y Q K G E I M S R S G H R T N L P F V
Km-AP21	M F V Y I T E I V S R Q R A N E L L V Y Y V F H F P R R V S T T V Y T P V E
	E I A L A L R R A N A S P L A Y V E E T I M A F T P V R H M V G Y T P L
	R V E E R H L N V T S P F E R K L L E R L V K N A L I Y T A E R N L Q A R
	K I G H A L M L N Q I M E I E E I L I Y E A V E L H V E I I D D K V L L G F
	D L N H R F D Y T Y T L Q D M I K K G K S I R P G L K V V Y S D G K T R
	Y A F T V V N I A P F G V F D R C P I L Q T S I Y D Y Y C Q K G A D H V L
	R L F T R S T R V I Q V R S F F E R L K Y A A T L L R P L C T L E T M E P
	D Q V E N V S K A I K L S S N Q R M R L T L R L L S E I E A Q Y K H L T Y
	A P D P F T I A R Q G Y K L E E L A T P R V F F Q R D Y A T V V S G I K T
	G K L Y K G G N V R V S L L L D T D F K Q R F N I T D E D I Y A F I A L L
	E K I A E R Q G I N I T I S T A W K A V A G R Y T L D F F E H I A E K I L A
	L R P L F A Q S T V L A F I S G A F L S D E Y T R F Y N L L K M I F G G N
	M D I H S Q V I T E K T L Q A Y Q K L L E Q N G L L D F K P E D D E Q C
	D R A I E V L K N D S F Y Y T V L N I L L G M Y V K A G I E A W I L A N T
	L H S D L F I G L D I V S V D G V V G P G M M T V I G S Q G H L V K R I R
	G P G Y L P G E K L R A T L L A D H V E Q A I K S Y H S E F N R F P K H
	I T I H R D G K S R E Q T A E M E K I C A H Y Q I T Y D I V E I L K K P N
	R R V A F F N S T T N T F S T K Q G T V Y Q R G N E A F L C A T N P Q Q
	K V G M A L P V R I F Q V T K T L P F S H I I E D I Y N L S F L H I A A L L
	K M R L P V T I D Y A D L I A T G Y Q R G K V M P R S G N R V N L P F V

Table S12: Sequence specifications of Km-APs (22-24).

Protein	Sequence
Km-AP22	M K P Y I T E I I S R E K A D E L K V Y T Y H F P R Y L S C N V Y I S V K<br>E I M Y A L R R A N G S P L A Y S D E T I L A F T P V R Q M V G Y T P E<br>K V E E R Y L N V T S P Q E R A L L E R L L K N S L I F T A E R H L Q F T<br>Q I G E S L R S V K V K E I E Q I L I H K S V E L D C E I I N D R I L M G F<br>D L V Y R F D Y T Y T L Q D M I E K G K S L R P G M K V V I S N S K V R<br>R T Y E L V N L A P Y G V F D R C P L L Q T S I Y Q Y Y V E K G A E Y V<br>L R L L T R S T R V I H V R S F F Q E L S F A A V L L K P L C T K E S M Q<br>P L Q V L N L S R I I K L S S S Q R M R D T L K Q L T Q V F A Q Y K H L T<br>Y A P N P L T I A R N G Y K L K E L S T P K V K F D R D Y A T I D S G L K<br>N S K L Y K G G N I K I S I L L D D D L K L K Q N I S E E D I Y S F I S V L K<br>K I A Q A Q G I N C T V S T A W K A V A G R F T K T F F E Q I D E L I L S<br>L E P L F T Q T T V L A F I S A A L E S D K Y T R F Y N L L K Q E F G G K<br>W D I C S Q V I T E R S L E A F Q R V L L Q H G L L D F R P N D D D Q C<br>N K V I D V L K N T E F Y Y T V M N I L L G I Y T K A G I E A W I L A N T<br>L H S D F F I G L D V T K F N G D T G H G M S T V I G S Q G Q L I K R V R<br>R S G Y E P G E K I K D S I L A N M L K Q T I K A Y H D R F D E F P K H V<br>T I H R D G F W K E N I A L V E K I M A H Y E I T Y D I V E I T K K P N R<br>R F A F Y N S V T N N F S T K Q G L I Y K I G N E A Y L C A T D P E Q R<br>V G M S Q P I K I E Q V S R T L P F D E I V E D V Y E L S K L H I H S V N<br>K M R L P A T I H Y A D L F A T A Y E R G E I Q V K S G H R T S L P F V
Km-AP23	M Q P Y I T E I Q A R T K A N E L E V Y V Y V F P R K D S T V V R L S V K<br>H I M E S W Q R A N G Q P L A Y V E I T I V A F T P V R H M C G Y T P L<br>R V E K R K L N V T S P Y D R A L L E R L V K N S L I F T A E R N L Q A Q<br>Q S G K A L Q S H F V K Q I E N V L I Y E A V E F N V N I V D D E I V I G F<br>D L T Y Q T D Y T E T L Q S M I E R G K S V R P G Q R V V H G N S K T L<br>Y A Y E V V H V A P Y G V F D R C P L L Q T S I Y Q Y Y V E K G A Q H V<br>L R V F T R S T K L I Q V R T L E Q K L S Y A A V L V K P L A T L E T M E<br>P Q Q V I N I S K L I K L S S S K R M Q T T M R I I S E L Y A N Y R H L T F<br>A P N P F T I A R N G Y K F R E L S E P K V Q F D R D F A T A V A G V K<br>T A K L Y K G G N I K I S L F L D T D L Y L K H E V T D H D I Y T F I A L<br>L K K Q A E S H G V N V T V S T A I K A V A G R F T E D F F E Q I D E K I<br>K A L K P L I A Q T T V I A F I P A A L L S D E K T R F Y K V L K Q V F G<br>G K D D I A S Q V I T E Q T I S A F Q K V L E K H G L D N F K P N D Q D Q<br>I D K A I D V L Q N D S F Y Y T V Y N I L L G I Y V K A G I V A W I L A E T<br>L H A D F F I G L D V T K K N G K L G T G M A T V I G S Q G Q L I L R Q P<br>G P S Y E P G E K I R A D L L A D I I K Q I I R A Y H D R F R E K P R H I T<br>I H R D G F W R E D T A I I E K I M S H W E I T Y D I V E I I K K P N R R L<br>A F Y D S D T D T W S T K Q G T V Y R K G D S A Y L C S T Q P Q Q K V<br>G M A Q P V K L H Q V T R T L P F S H V V E D V Y Q L S R L H N H S V N<br>K C R L P A T I Y Y A D K F A N A Y E R G E V H V R S G N R T S L P F V
Km-AP24	M E P Y I T E I V S R E K A N E L K V F E F T F P R Y E S D T V W L S V K<br>E I M E G L R R A D D L P L A F V E I T I V S F S P V R H L V G Y T P L N<br>V Q E R H I N V T S P F E R A L L E K L L K N S V I F T A E R H L Q A E Q<br>I G K E L Q S H K T M E I E E V L I Y K A I E L T V N I S D D R I L C G F D<br>L N H Q F D Y T R T L Q D M I E K G K T V R P G L R V I I A N S Q T L Y<br>T Y T L I N I A P Y G V F D R C P L F Q T S I Y D Y Y V K K G A Q H I L R<br>L L T R S T R V I Q V R S F F E K L S Y A S T L L K P L A T Y E T M E P V<br>S V L N V S R L I K L S A D Q R M K L T M R L L S E I Y S E Y K Y L T F A<br>P N P F S I S R N G Y K L R E L A T P K V H F D R D Y A T V V A G V K T<br>G K L Y K G G N I K I A L L L D S N L K L K M H I T E E D I Y T Y I A L L<br>E Q I A I A Q G V D V T I S D A H K A V S G K F T Q D F F E N I A L K I T<br>S L E P L F S Q T T V I A F I S G T E R S D E K T R F Y E L L K K F F G G<br>Q W D I S S Q V I T E R T L Q A F Q K L L D E H G L T D L K P D D N Q Q<br>C D K V I N I L K N S E F F Y T V Y N I L L G I Y V K A G I Q P W I L A N<br>T L H S D L F I G L D V V H K N G N L G G G M V V V I G S Q G E L V K Y<br>V R Y P G F F S G E K I F S D L A D Q L K R V I K S Y E D K F D E K P K<br>H I T I H R D G F Y R E D T A L V E K I M S H F E I T Y D I V E V I K K P<br>N R R L A F F N S T T K T F S T K R G T V Y E R G D T A Y L C S T D P L<br>E K V G M A Q P I K I H Q E T K T L P F E E I I E D I F Q L S K L H I H S M<br>L K M R L P A T I H Y A D L I A T A Y Q R G E V M A Y S G N I T S L P F V

Table S13: Sequence specifications of Km-APs (25-27).

**Protein**	**Sequence**
Km-AP25	M Q P Y I T E L M S R E R A D E L R V Y V L H F P R R L S D T V F L G I K<br>E I M Y A W E R A N G Q P L A Y V E E T I V A F T P I R H K V G W T P Q<br>R V E E R H L N V T S P F E R A L L E R L V K N S L I F T S V R H L E F K<br>R I G K S L R S H K V Q E I E E I L I H E A V E L T V N I K D D Q I L V G F<br>D L T H Q F E Y T Y T L Q D M I E K G K P I R P G M K V I I S N S F V R Y<br>T Y E L V Y V A T Y G V F D R C P L L Q T S V Y Q Y Y N E K G S E H L L<br>R L L T R S T R V I E V R S Y F Q K L I W A A S L L K P L C T R E T M E P<br>Q S Q L N L A K S I K L S S S E R M Q D S L R Q L S E V Y A H Y R H L T Y<br>P P D P F T I S R Q G Y K R K E L A T P K V Q F D R D Y A T V V S G V K<br>T D K L Y K G G N V K I S V Q L D S D L K Q K H Q I T N E D L Y A F I S L<br>L Q K L A E A Q G V N V T I S T A W K A I S G K F T L T F F E H I A E R I<br>T A L R P L F A Q T T V I A F I S G S F L A D K K T R F Y E L K K Q K F G<br>G K W N I A A Q V I T E R T I E A F R K L L D Q N G L D D L K P N D D D<br>Q I N K V I N V L K N D E L Y Y T V I N I L L G V Y V K S G I E P W I L A N<br>D T H A D L F I G L D V N K K N G V Q G P G M S S V V G K Q G Q L V Q R<br>A R D L G Y Y P G E K I R A D L L S T Q L E R T I K S Y E S R Y Q R Y P E<br>H I T V H R D G F W R E Q V S L V E K I F S H Y Q I T Y D I I E I I K K P N<br>R R V A Y F N S D D E N W S T R R G L I Y Q R G N E A F L C S T N P R E<br>K V G M A Q P I R I H Q N T R T L P F E Q I I E D V Y Q L S K L H I A S L N<br>K M R L P V T I Q Y A D L I A T A Y E R G Q I M P R S G N R T T L P F V
Km-AP26	M D A F I T E I V S R E R A D E I E V Y T F T F P R K L S T V V R L S V E<br>Q I M R G L Q R A D N S P L A Y V E E T I V A F S P I R H R V G Y T P M<br>K V E K R H L N V T S P F E R S T L E R L I K N S L I F T G E R H L H A E<br>K I G K A L E S V Q T Q E I E E V L L H N A V Q L T V N I V D N R I I C G<br>F D L T Y R F D Y T Y T L Q S L I E Q G K P I R P G E R V I H S D S E V R<br>Y V Y E V V D I A P Y G V H D R C P L L Q T S I Y Q Y Y V K K G S K H I<br>L R L L T R S T K V I Q V R T W F Q K L I Y A A T L L K P L C T K Q T M<br>E P V Q I E N V S R V I K L S S S E R M K T T L R L L D E V Y G E Y K H L<br>T Y A P N P F T I S R Q G Y K L K D L S T P K V H F E K D F A T I V S G V<br>K T G K L Y K G G N I R I S V F L D D E W K L K L E V T N E D I Y A F I A<br>L L K H I A I A Q G I T I T I S D A I K A I S G Y F T I D F F E E I S N K I Q<br>A L K P L F A Q T V V L A F V S G S L E S N E Y T R W Y N L L K Q F F G<br>G K D D I C S Q V I T E K T I E A Y Q K I L E K H G L E N L K P N D Q E Q<br>I Q R A I N I L K D D S F Y Y T V I N I L L G V Y V K S G I A P W I L A N D<br>T H A D L F I G L D V V H K N G N F G T G M A S V V G S Q G H L I R Q S<br>R G S S F E P G E K I R A D N L S N Q I K K V I K S Y E S R F Q E F P R S I<br>V I H R D G F Y R E E V A L M E K V F A H W E I T Y D I V D V I K K P N<br>R R I A Y Y N S T T N T F S T K Q G L I Y E R G N T A F L C A T D P L Q R<br>V G M A Q P V K I F Q N T R T L P I E E I I E D V F Q L S K L H I H A M L<br>K C R L P M T I D Y A D K A A T A Y E R G E I N A R S G N K T S L P F V
Km-AP27	M E A Y I T E I V S R E K A D E L K V Y Y V L H F P R K L S T T T F L S V K<br>E I K Q G W Q R A N G L P L A Y A E E T I V A F S P V R H M V G Y T P Q<br>R V E E R K I N V T S P F E R A L L E R L L K N A L I F T A Q K H L Q A Q<br>K I G K A L Q L T Q V Q E I E R I L I H K A V E L E V N I I D N R I V I G F<br>H L T Y Q F D Y T Y T L Q D M I E K G K P I R P G M R V I E S N S E T F K<br>T Y E V V N V A P Y G V F D R C P L L Q T S I Y Q Y Y V E K G A Q F I L<br>R T F T R S T R V V E V R S F F Q K L I F A A T L L K P L C T K E T M Q<br>P Q Q V L N V S R L I K L S A S K R M K T T L R L L S Q V Y A Q Y R H L<br>T F A P N P L T I S R N G Y K L K D L S T P K V H F D R D Y A T V T S G<br>V K S G K V Y K G G N I K V S L L L D S D F K L K H N I T D E D I Y Q F I<br>A L L K K L A I A Q G V T C T I S T A W K A V S G R F T L D F F E H I A E<br>K I E S L K P L F A Q T V V L A F I S G S L L S D E K T R L Y E L L K E V F<br>G G K D D I P S Q V I T E R T L E A F Q R L L D K H G L I D F K P D D Q D<br>Q I N K A I D I L K N S S F Y Y T V L N I L L G I Y V K S G I Q P W I L A N<br>T T H S D F F V G L D V V E K N G N N G H G M S T V V G S Q G Q L I K R<br>Q R G K G Y L P G E K I R A D [illegible] L A N M I E Q T I K A Y E S L F K E F P H<br>H I T I H R D G F F R E E I S L M E K I M A H Y E I T Y D I V E V I K K P N<br>R R V A F Y N T V T N T F S T K Q G L V Y K R G D E A F L C S T N P L Q<br>K V G M A Q P I K I E Q V T K T L P F E H I I E D V Y C L S K L H I H S L N<br>K M R L P L T I H T A D L L A N A Y E R G Q I M P Y S G N K T S L P F V

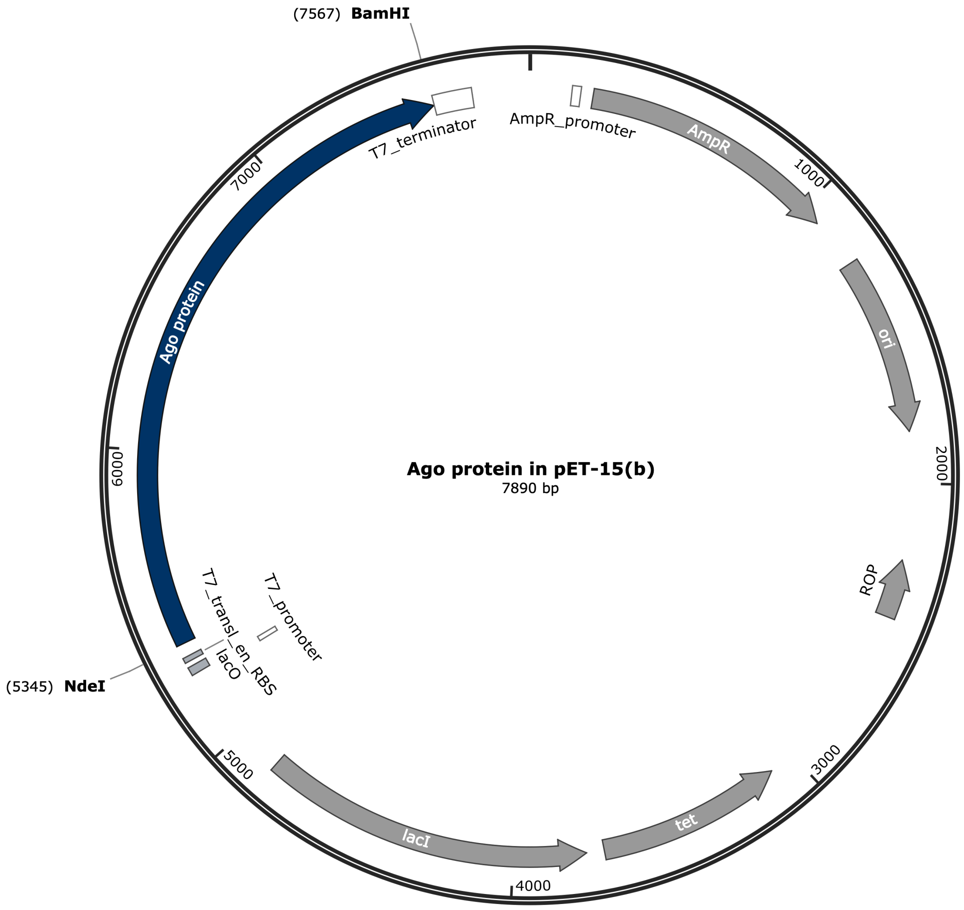

Fig. S48: The construction of plasmid of KmAgo and Km-APs linked with GFP.

Table S14: SAXS data analysis of Km-WT and Km-APs.

Protein	Rg (nm) (from Guinier)	Rg (nm) (from P(r))	Oligomeric state
Km-WT	3.17	3.16	monomer
Km-AP2	3.19	3.16	monomer
Km-AP7	3.13	3.17	monomer
Km-AP16	3.20	3.16	monomer
Km-AP21	3.17	3.16	monomer
Km-AP22	3.18	3.16	monomer
Km-AP23	3.19	3.21	monomer
Km-AP25	3.23	3.17	monomer

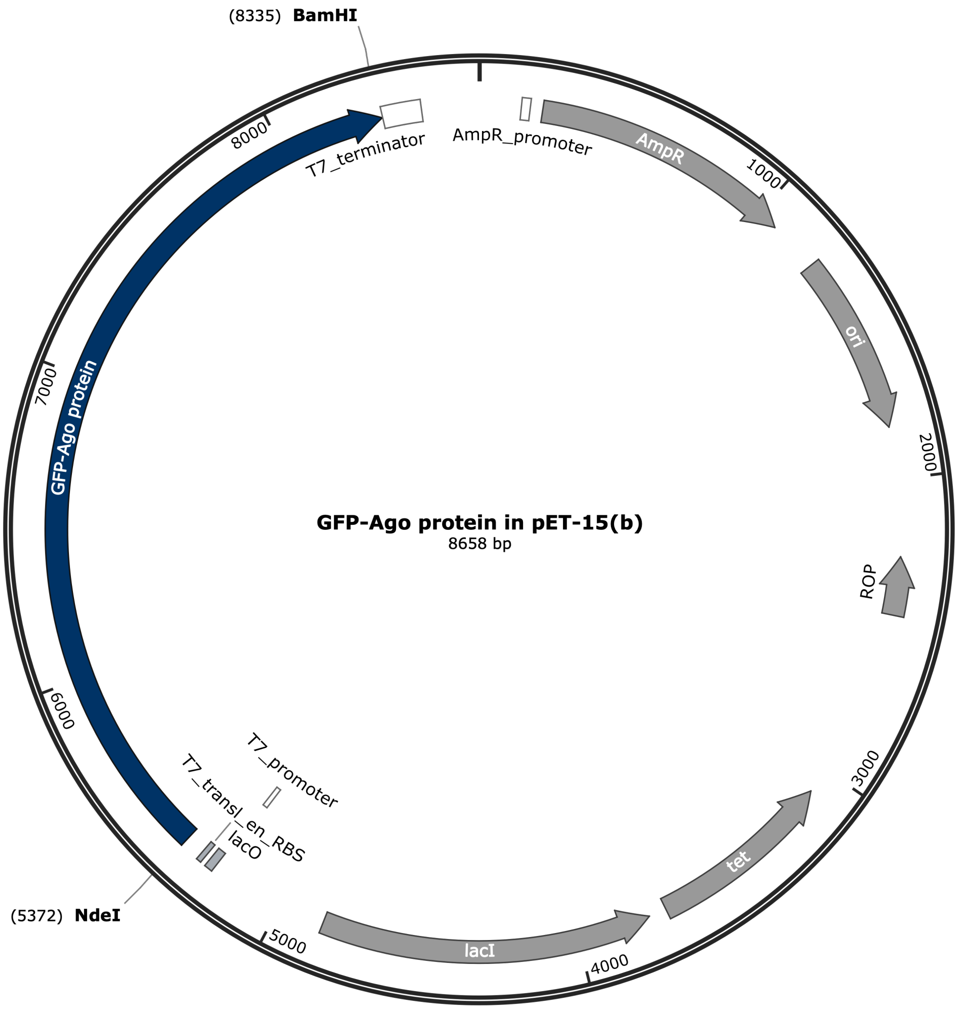

Fig. S49: The construction of plasmid of Km-WT, Km-APs, Pf-WT, and Pf-APs.

Table S15: Michaelis-Menten analysis of Km-WT, Km-AP23, and Km-AP9.

**Protein**	**T (°C)**	$\boldsymbol{K}_M(\mu M)$	$\boldsymbol{k}_{\mathrm{cat}}(\mathrm{min}^{-1})$	$\boldsymbol{k}_{\mathrm{cat}}/\boldsymbol{K}_M(\mu M \cdot \mathrm{min}^{-1})$
Km-WT	37	7.14±0.32	(7.96±0.56)x10^{-4}	1.11x10^{-4}
Km-AP23	37	1.03±0.50	(3.97±0.45)x10^{-3}	3.85x10^{-3}
Km-AP9	37	21.83±0.29	(2.80±0.41)x10^{-4}	1.28x10^{-5}

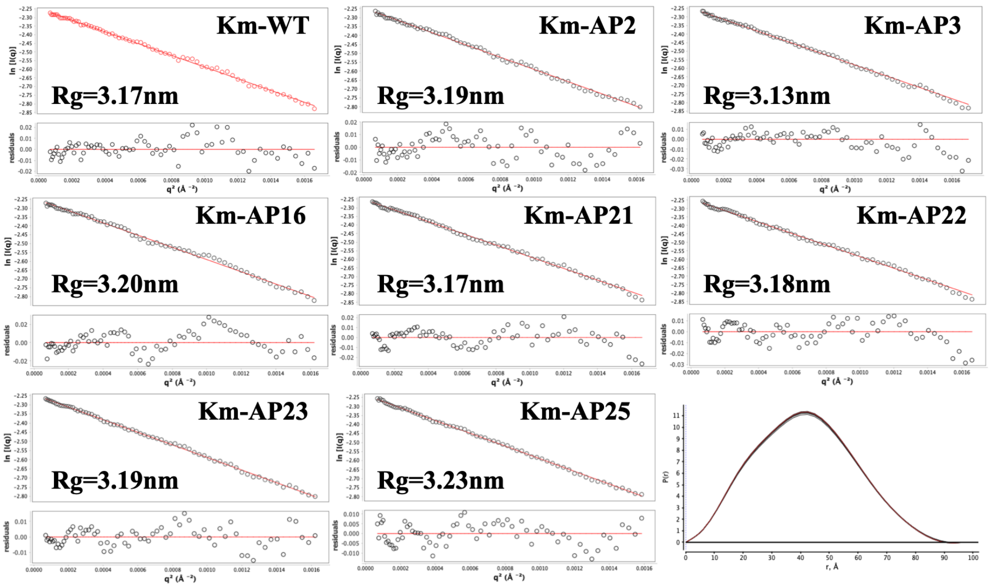

Fig. S50: Guinier plots of of Km-WT and Km-APs. The lower insets show the error weighted residual difference plots for the Guinier fitting. Last panel represents the pair distribution of Km-WT and Km-APs obtained from SAXS.

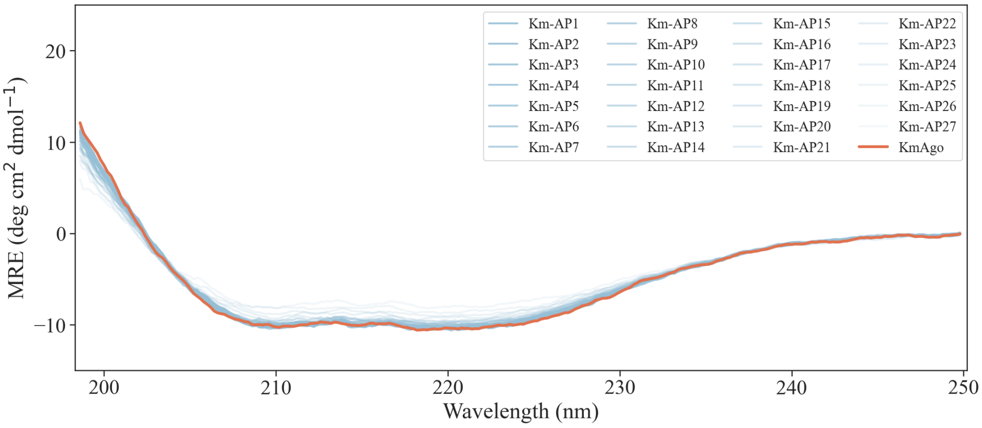

Fig. S51: CD spectra of KmAgo (red) and 27 Km-APs (blue).

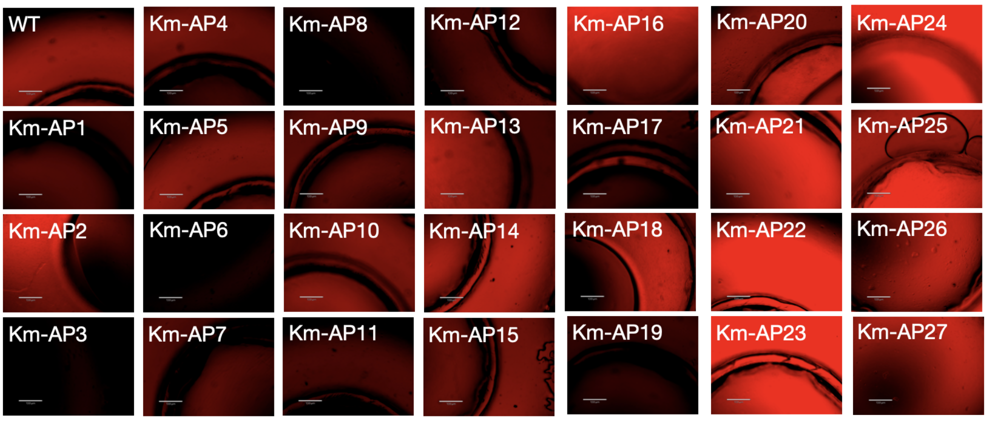

Fig. S52: Cleavage assay achieved by KmAgo and Km-APs expressed in vitro. Notice that the fluorescence intensity is not normalized by the concentration of proteins, this assay only reaches a qualitative conclusion on whether ssDNA is cleaved by the input protein.

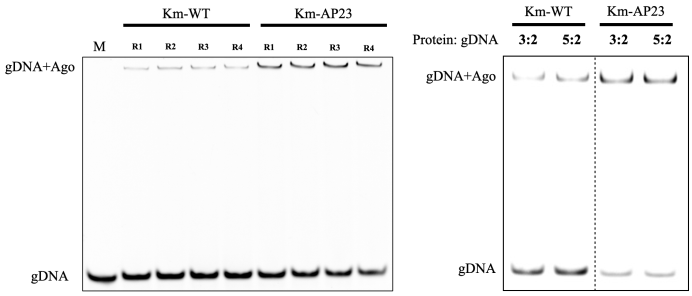

Fig. S53: Electrophoresis mobility shift assay of the binding of Km-WT and Km-AP23 to gDNA at different concentration ratios.

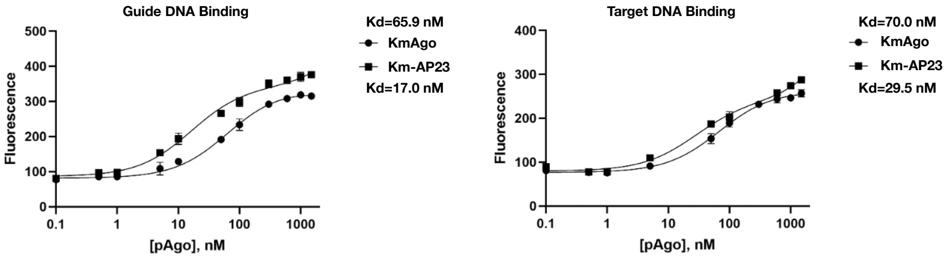

Fig. S54: Fluorescence polarization assay of the binding of KmAgo and Km-AP23 to gDNA and tDNA.

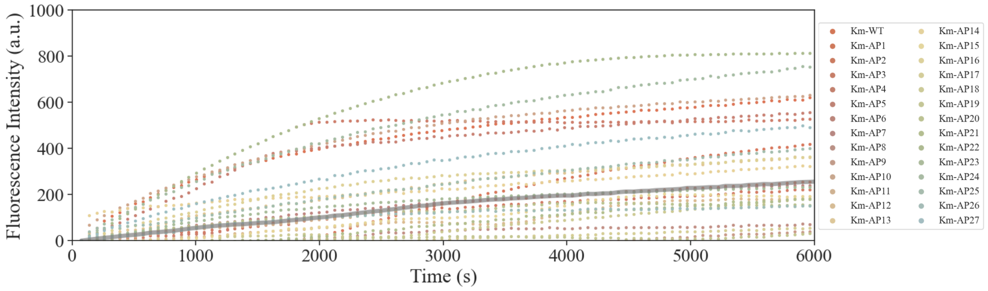

Fig. S55: The cleavage activity of KmAgo and Km-APs at 37 °C is traced by fluorescence intensity as a function of time. The cleavage for Km-WT is highlighted in gray for a clearer comparison.

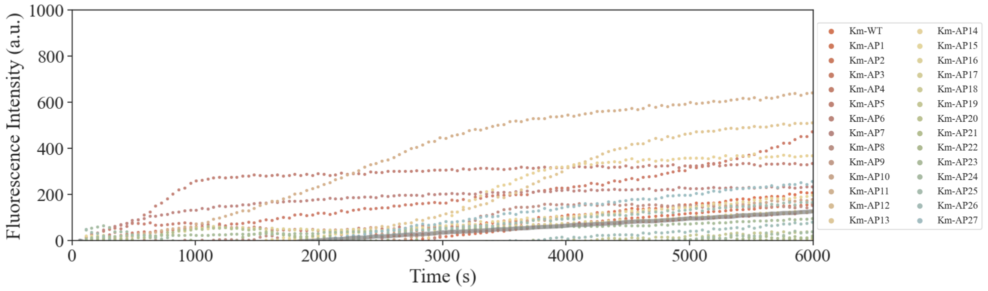

Fig. S56: The cleavage activity of KmAgo and Km-APs after incubation at 42 °C for 2 minutes. The cleavage experiment is conducted at 37 °C and is traced by fluorescence intensity as a function of time. The cleavage for Km-WT is highlighted in gray for a clearer comparison.

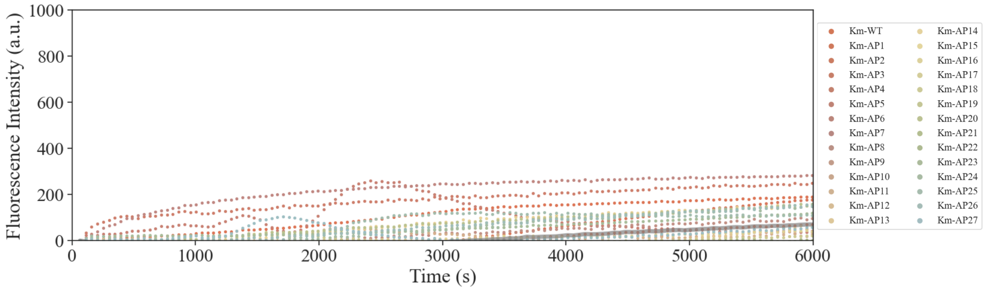

Fig. S57: The cleavage activity of KmAgo and Km-APs after incubation at 42 °C for 5 minutes. The cleavage experiment is conducted at 37 °C and is traced by fluorescence intensity as a function of time. The cleavage for Km-WT is highlighted in gray for a clearer comparison.

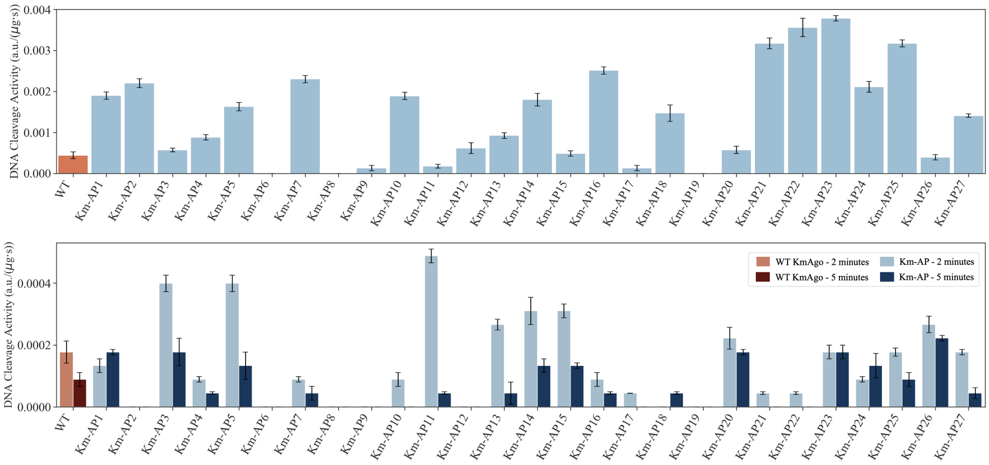

Fig. S58: The protein quantification of activity experiments of Km-APs.

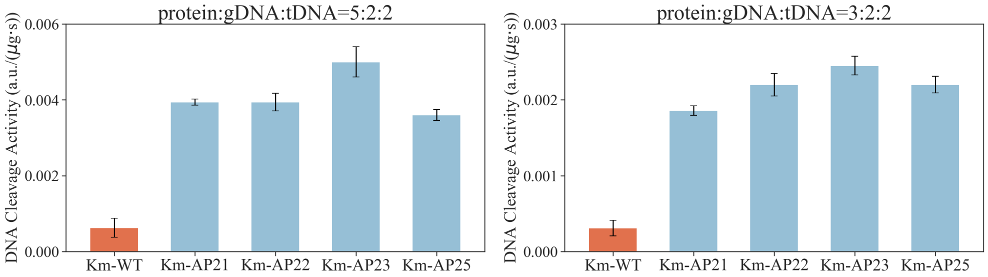

Fig. S59: The cleavage activity experiments of Km-APs under different ratios of protein:gDNA:tDNA.

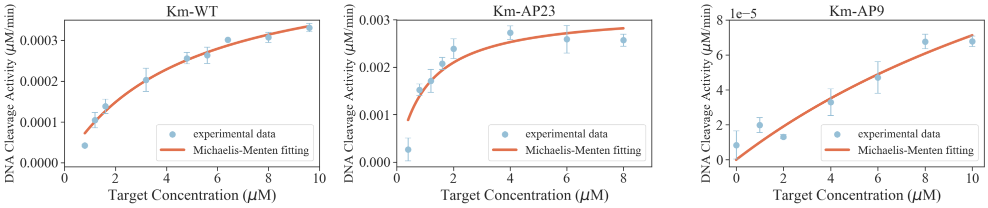

Fig. S60: Michaelis-Menten kinetics analysis of Km-WT, Km-AP23, and Km-AP9.

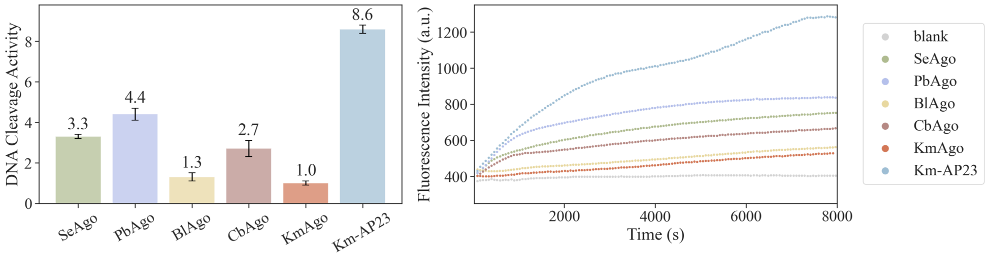

Fig. S61: Left panel: The cleavage activity of SeAgo, PbAgo, BlAgo, CbAgo, KmAgo, and Km-AP23 at 37 °C. Right panel: The cleavage activity of SeAgo, PbAgo, BlAgo, CbAgo, KmAgo, and Km-AP23 at 37 °C is traced by fluorescence intensity as a function of time. The blank indicates the sample contains guide DNA and target DNA.

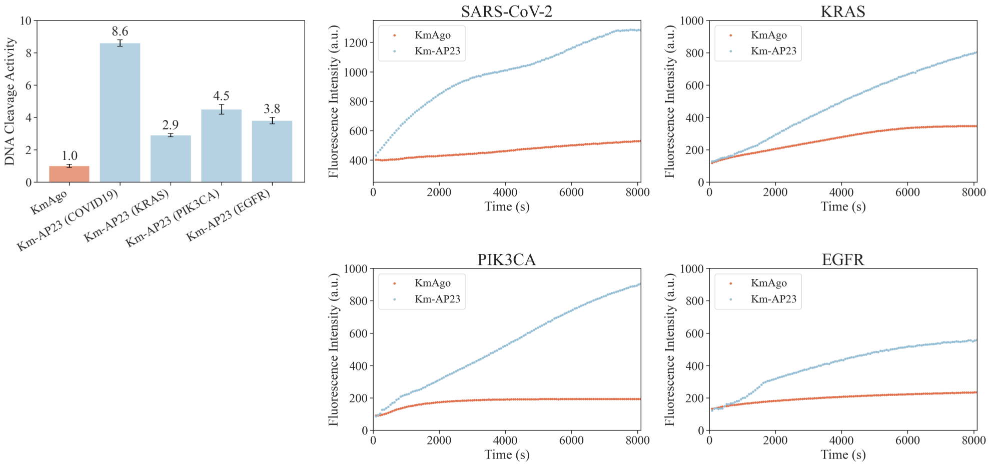

Fig. S62: DNA cleavage assay on different guide and target DNA sequences from various viruses and diseases (SARS-CoV-2, KRAS, PIK3CA, and EGFR) of KmAgo and Km-AP23.

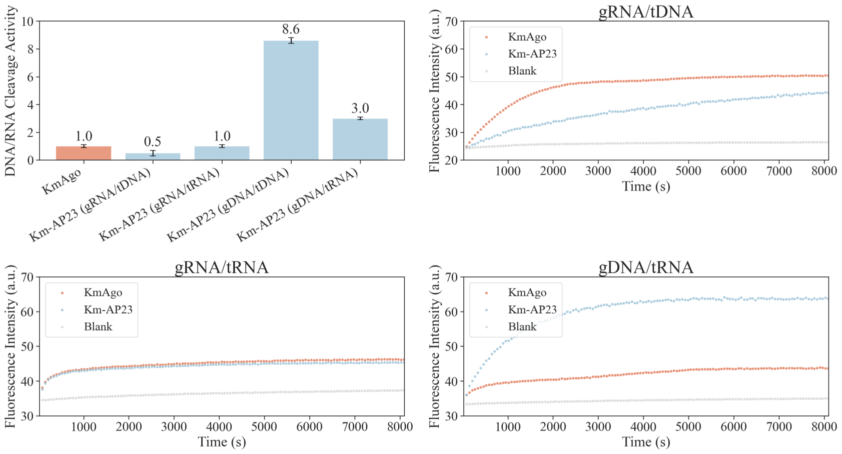

Fig. S63: Cleavage assay of KmAgo and Km-AP23 on different ssDNA/ssRNA targets when using different ssDNA/ssRNA guide. The blank indicates the sample contains guide and target nucleic acids.

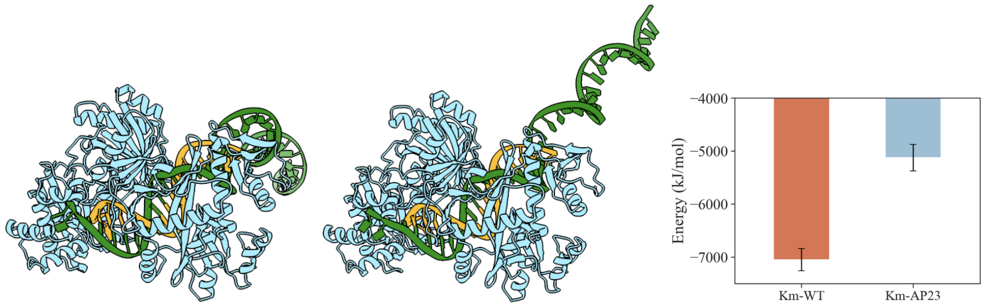

Fig. S64: Left panel: complex structure of Km-WT-gRNA-tDNA. Middle panel: complex structure of Km-AP23-gRNA-tDNA. Right panel: binding free energy of gRNA/tDNA for Km-WT and Km-AP23. gRNA, tDNA, and protein are highlighted in orange, green, and blue. The binding free energy is averaged over MD trajectory.

Table S16: Sequences of guide and target DNAs from different viruses and diseases.

Oligonucleotide	Sequence (5′-3′)	Description
SARS-CoV-2-gDNA	5′P-TGAGGTAGTAGGTTGTATAGT	
SARS-CoV-2-tDNA	5′FAM-ATATACTATACAACCTACTACCT CGTATAAATTTTTAAATAAAT-3′BHQ1	5′FAM labeled tDNA, 3′BHQ1
KRAS-gDNA	5′P-TTTGGAGCTGGTGGCG	
KRAS-tDNA	5′FAM-TAGCTGTATCGTCAAGGCACTCTTGCCTAC GCCACCAGCTCCAACTACCACAAGTTTATA-3′BHQ1	5′FAM labeled tDNA, 3′BHQ1
PIK3CA-gDNA	5′P-TCAAATCACTGAGCAG	
PIK3CA-tDNA	5′FAM-CTCCATAGAAAATCTTTCTCCTGCTCAGTG ATTTCAGAGAGAGGATCTCGTGTAGAAATT-3′BHQ1	5′FAM labeled tDNA, 3′BHQ1
EGFR-gDNA	5′P-TTTGCTTCTCTTAATT	
EGFR-tDNA	5′FAM-AAAATTCCCGTCGCTATCAAGGAATTAAGAG AAGCAACATCTCCGAAAGCCAACAAGGAA-3′BHQ1	5′FAM labeled tDNA, 3′BHQ1

Table S17: Additional Sequences of guide and target DNA/RNAs from nucleic acid preference analysis.

Oligonucleotide	Sequence (5′-3′)	Description
gDNA	5′P-AGAGGTAGTAGGTTGTAT	
tRNA	5′FAM-AAACGACGGCCAGUGCCAAGC UUACUAUACAACCUACUACCUCUU-3′BHQ1	5′FAM labeled tRNA, 3′BHQ1
gRNA	5′P-AGAGGUAGUAGGUUGUAU	
tDNA	5′FAM-AAACGACGGCCAGTGCCAAGCT TACTATACAACCTACTACCTCTT-3′BHQ1	5′FAM labeled tDNA, 3′BHQ1

Table S18: The number of hydrogen bonds and salt bridges around Km-WT and Km-APs catalytic motif DEDD.

Protein	Hydrogen Bond	Salt Bridge
KmAgo	12	2
Km-AP9	10	2
Km-AP8	10	2
Km-AP19	9	2
Km-AP21	13	2
Km-AP22	13	2
Km-AP27	12	3

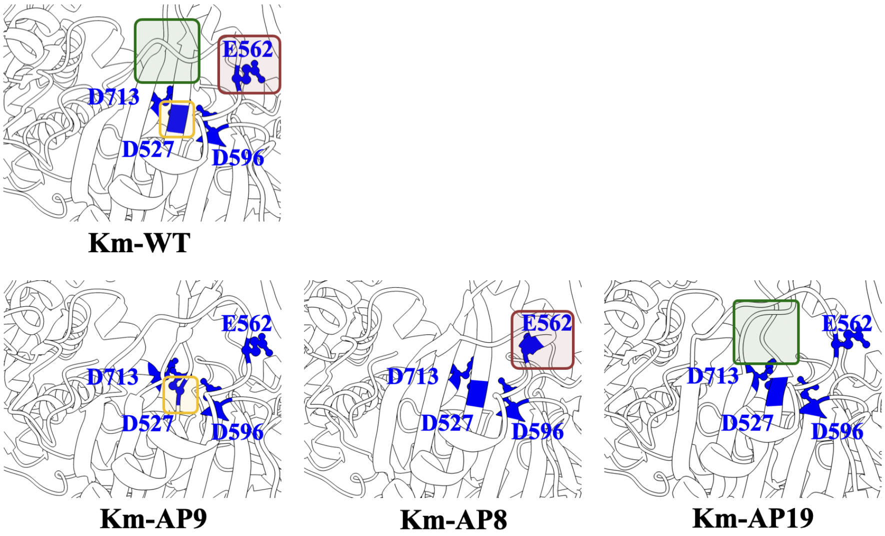

Fig. S65: Catalytic motif of KmAgo, Km-AP9, Km-AP8, and Km-AP19.

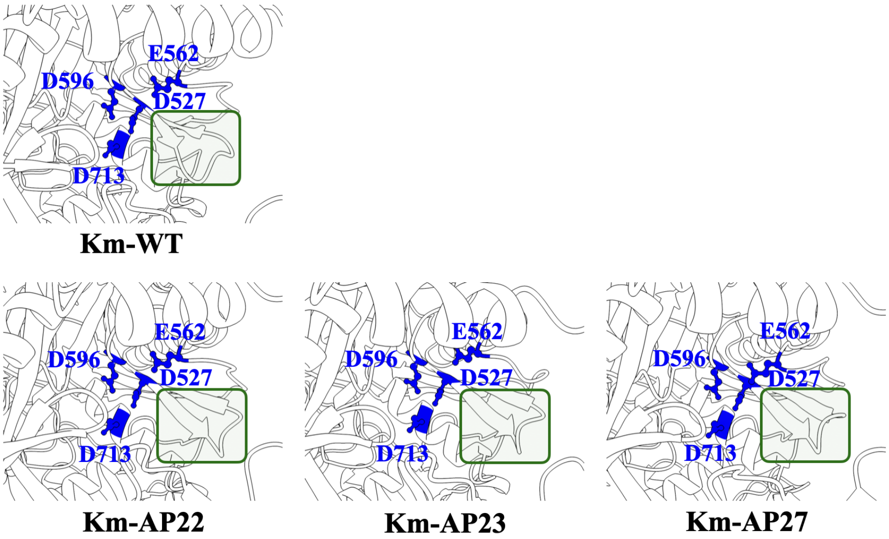

Fig. S66: Catalytic motif of KmAgo, Km-AP22, Km-AP23 and Km-AP27.

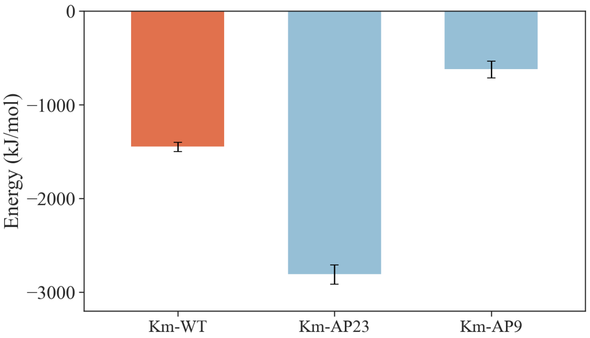

Fig. S67: The binding free energy of gDNA/tDNA in the catalytic pocket of Km-WT, Km-AP23, and Km-AP9 is averaged over the MD trajectory.

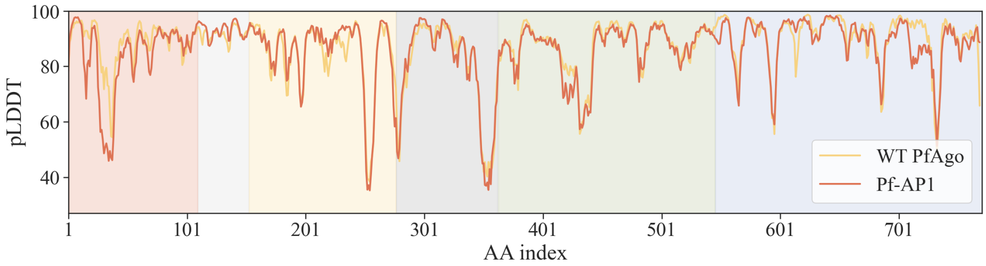

Fig. S68: Comparison of pLDDT of WT PfAgo and Pf-AP1.

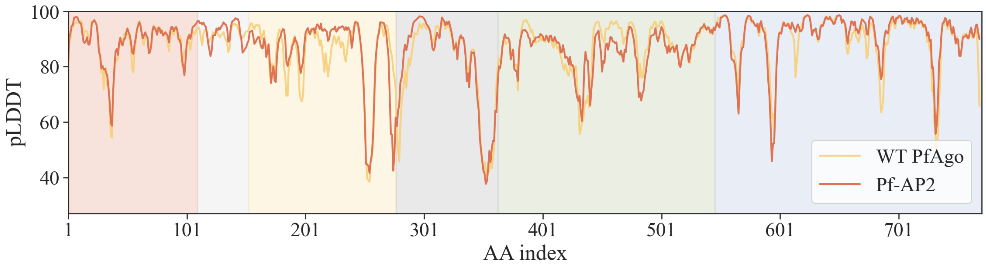

Fig. S69: Comparison of pLDDT of WT PfAgo and Pf-AP2.

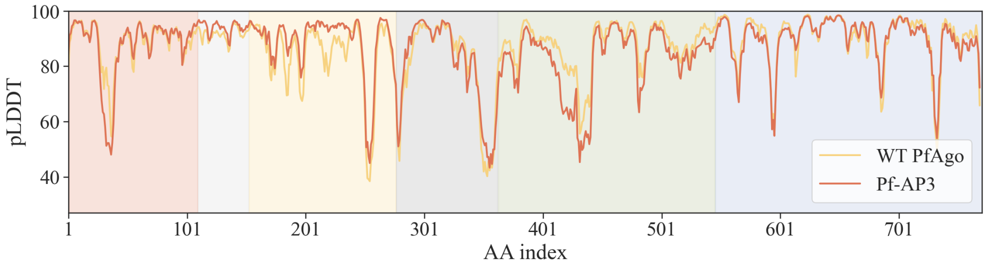

Fig. S70: Comparison of pLDDT of WT PfAgo and Pf-AP3.

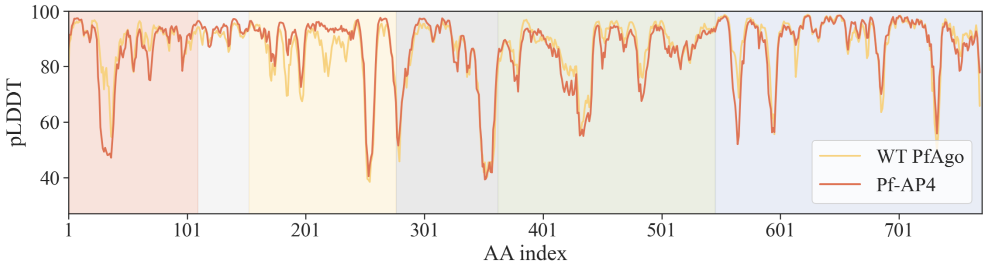

Fig. S71: Comparison of pLDDT of WT PfAgo and Pf-AP4.

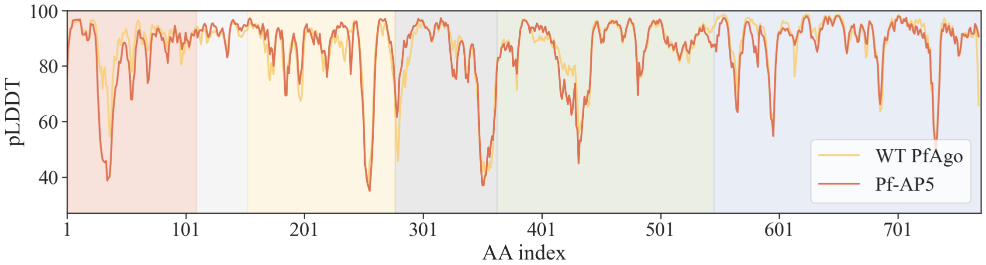

Fig. S72: Comparison of pLDDT of WT PfAgo and Pf-AP5.

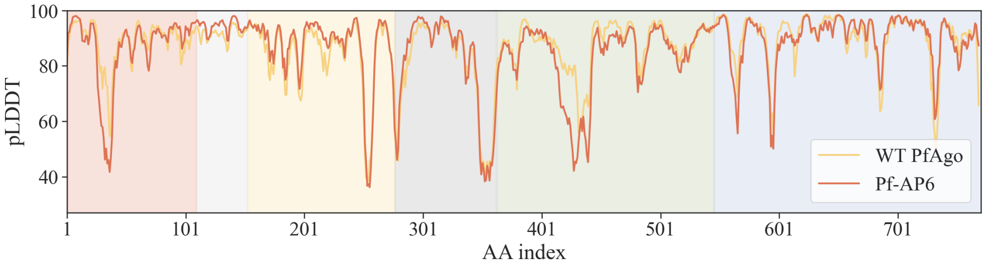

Fig. S73: Comparison of pLDDT of WT PfAgo and Pf-AP6.

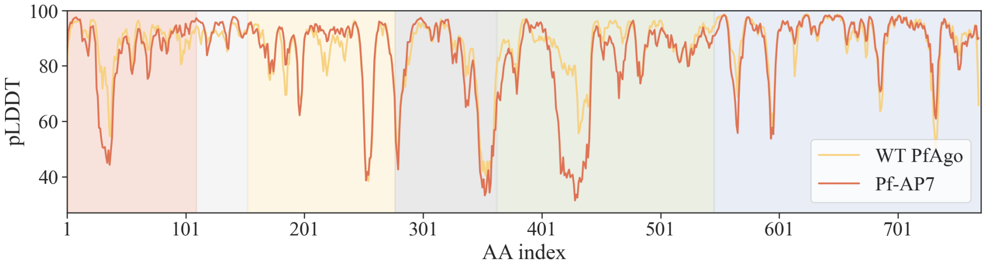

Fig. S74: Comparison of pLDDT of WT PfAgo and Pf-AP7.

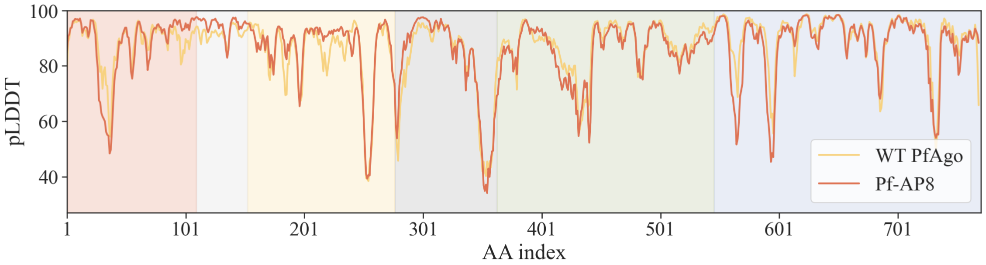

Fig. S75: Comparison of pLDDT of WT PfAgo and Pf-AP8.

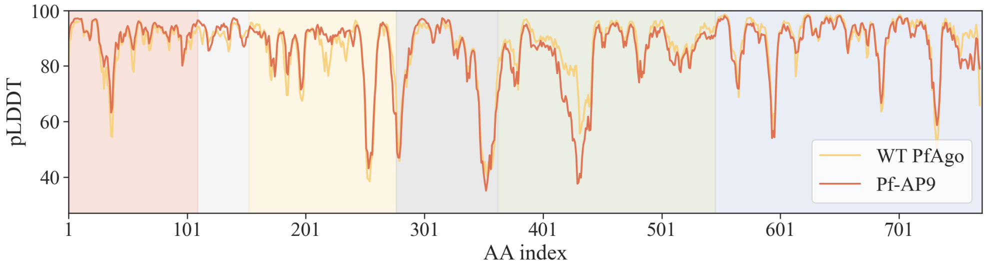

Fig. S76: Comparison of pLDDT of WT PfAgo and Pf-AP9.

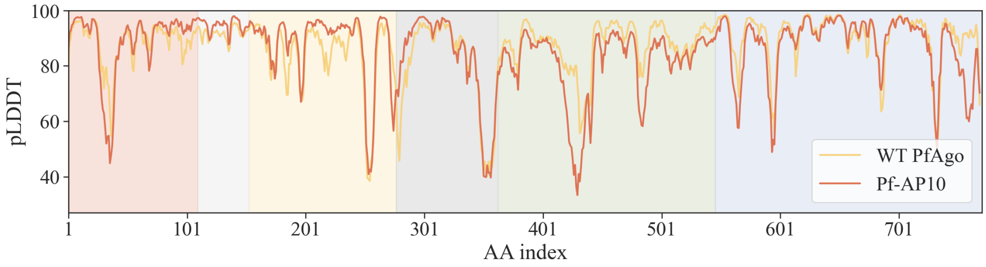

Fig. S77: Comparison of pLDDT of WT PfAgo and Pf-AP10.

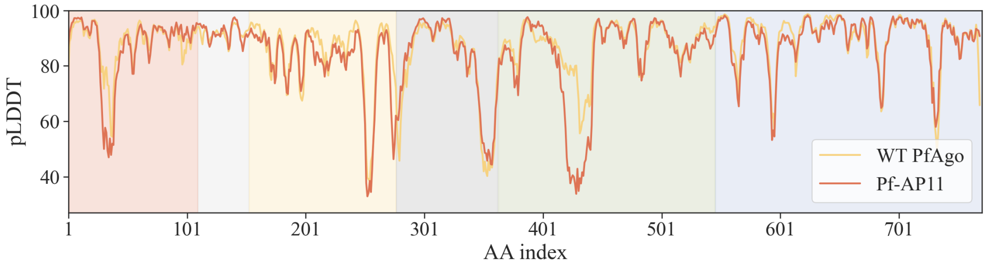

Fig. S78: Comparison of pLDDT of WT PfAgo and Pf-AP11.

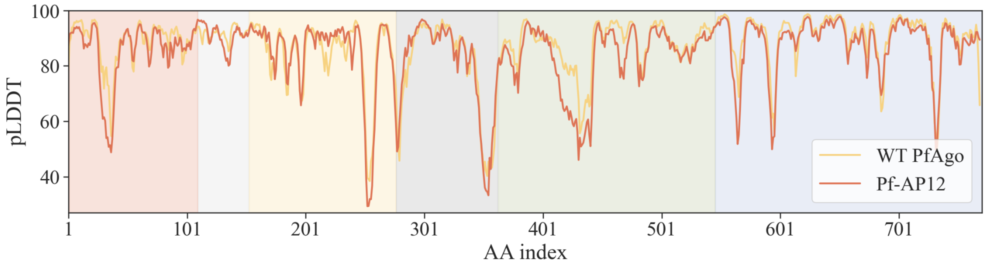

Fig. S79: Comparison of pLDDT of WT PfAgo and Pf-AP12.

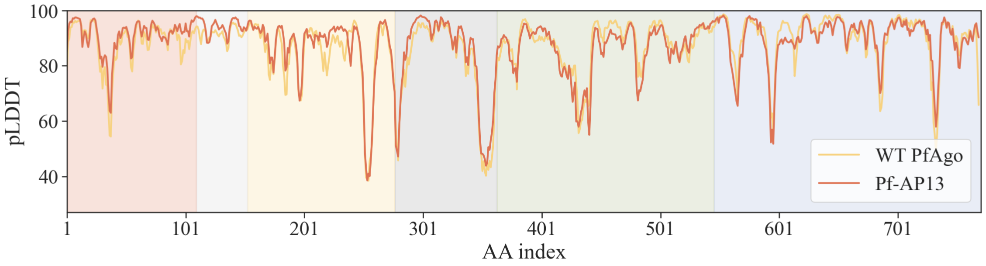

Fig. S80: Comparison of pLDDT of WT PfAgo and Pf-AP13.

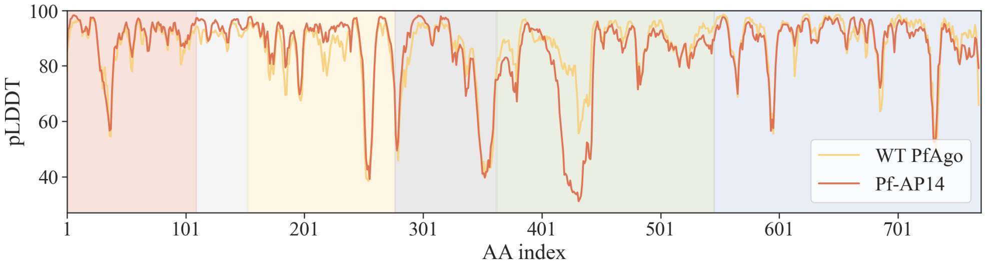

Fig. S81: Comparison of pLDDT of WT PfAgo and Pf-AP14.

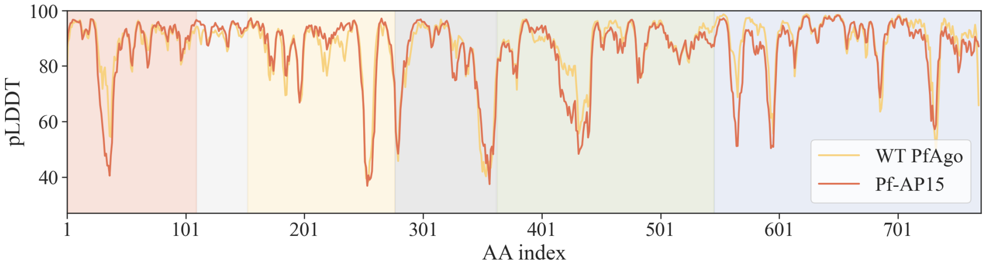

Fig. S82: Comparison of pLDDT of WT PfAgo and Pf-AP15.

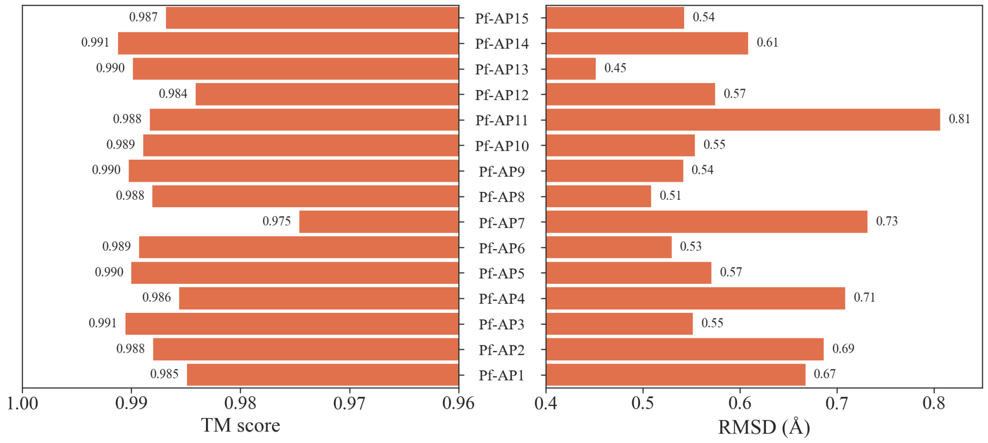

Fig. S83: RMSD (right) and TM score (left) of 15 Pf-APs.

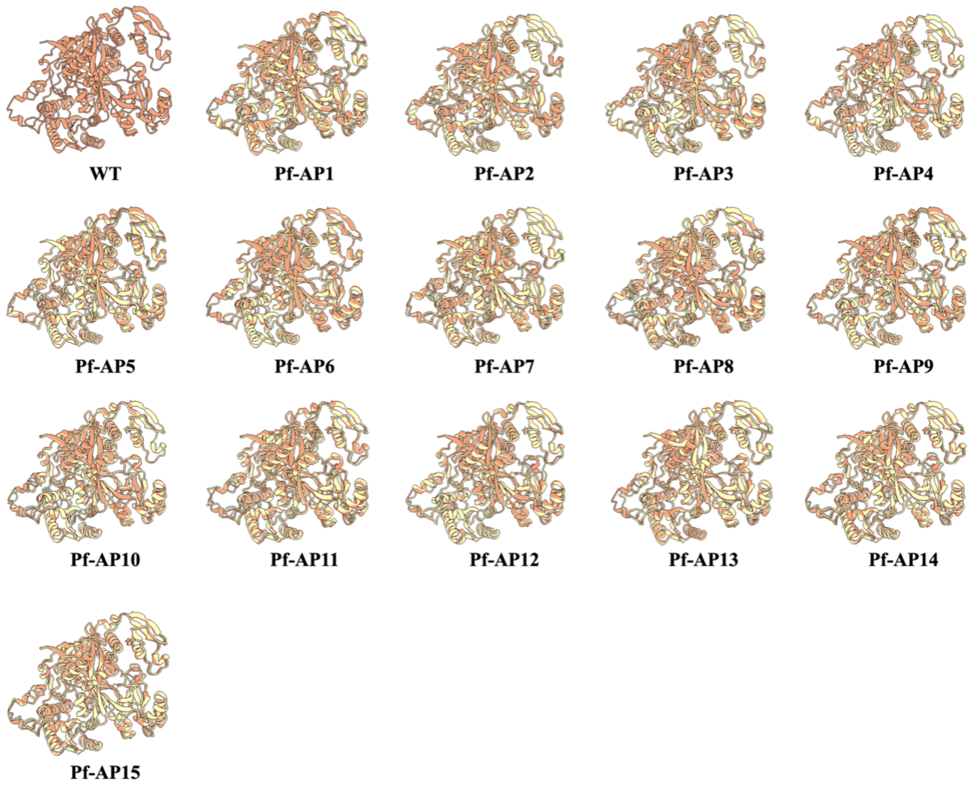

Fig. S84: Structural comparison of WT PfAgo and Pf-APs folded by AlphaFold2.

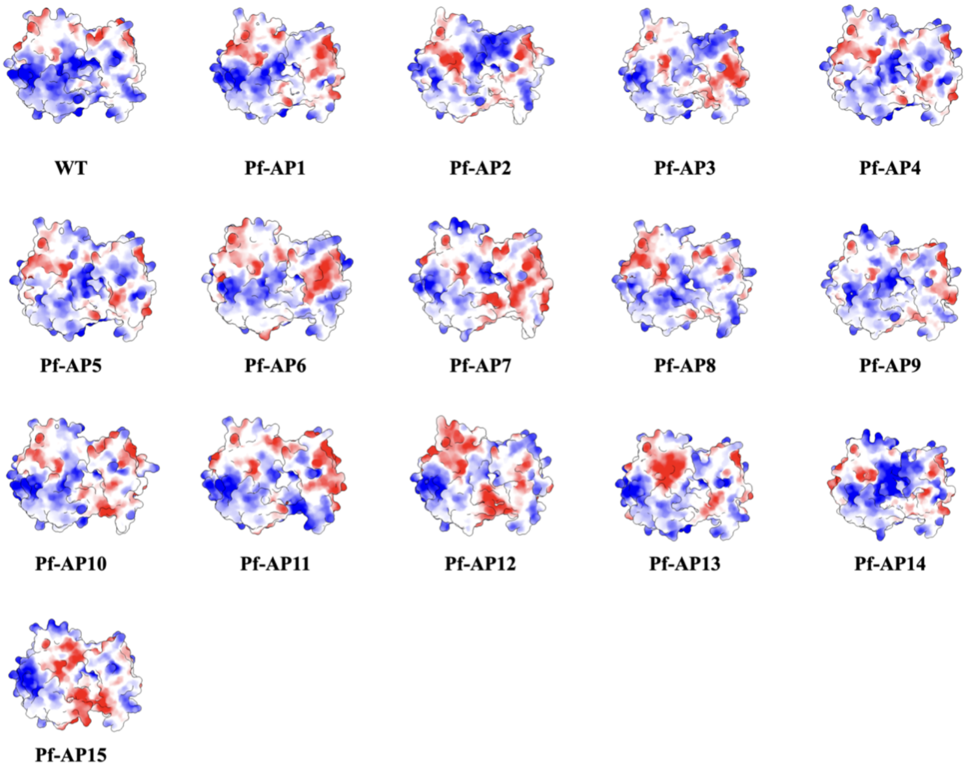

Fig. S85: The electrostatic surface of MID domain and PIWI domain in PfAgo and Pf-APs.

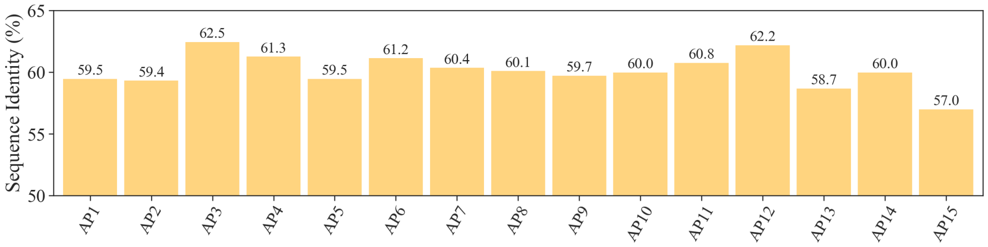

Fig. S86: Sequence identities of Pf-APs with the WT PfAgo.

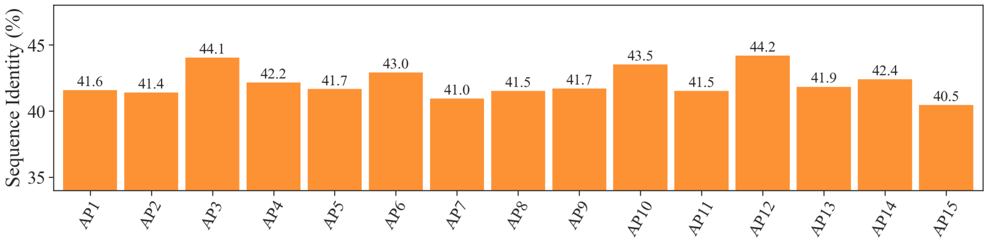

Fig. S87: Sequence identities of Pf-APs with the most similar pAgo proteins (excluding WT PfAgo) in the training dataset.

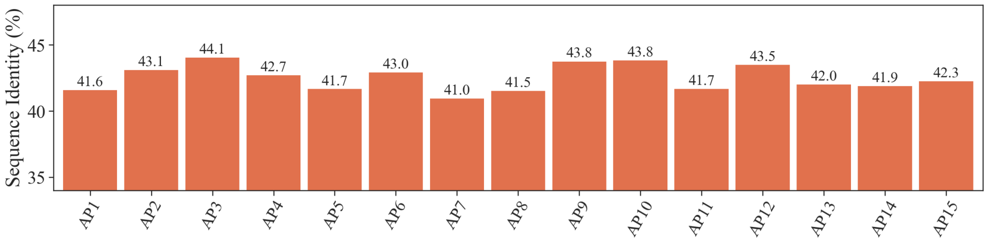

Fig. S88: Sequence identities of Pf-APs with the most similar protein sequence in NCBI NR (excluding WT PfAgo).

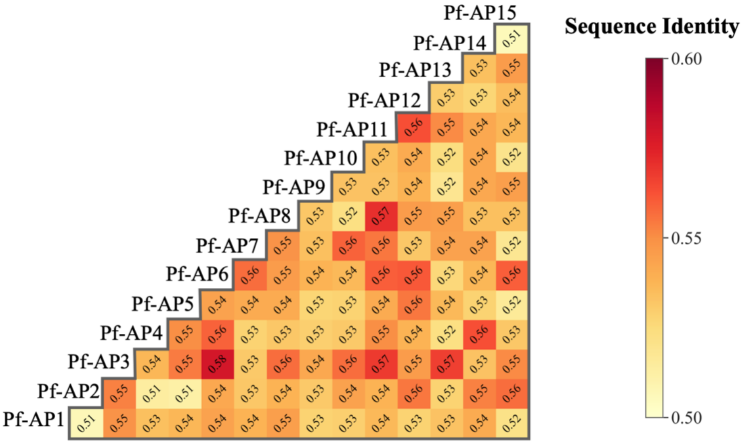

Fig. S89: Pairwise sequence identities of 15 Pf-APs.

Table S19: Sequence specifications of Pf-APs (1-3).

**Protein**	**Sequence**
Pf-AP1	M H A R V V I N L I K I D K T L I P E K I Y L Y K L Y Q D P E L E L K R D<br>G F T L F R L A Y E N N G I V L D P E N N V I A S T R I I Q Y E G E Y D P<br>E G E V S F S Q L R D G D Q S E F I L S L F N E H G L G E F S L S K I L Q K<br>H Y K P K T F G N Y E V V P F V Q L N V I K H E D E Y Y L V I N I V H E V<br>Q S M K T F A Q L V D R D P Q Q L I E Y A R K H K N N L M L Q D I S T P<br>E K T T Y R P L F K E Y Q E K P K L D Y N E L I K H Y F Y N H H I E R Y<br>W D V P I G K E E F F R K F G Q V D T H Q P A I L A K F A N K V R T T E<br>N F R V Y A L P Q F V V P K F N A S Q M E N D V S K E V T E Y T K L K P<br>E E R R K L L E N V L S K I N S D I I D K T L N Q V E C D K I S F E S E N R<br>I R I R D D K G N T I P V S H I S V E A A E L L N K S L Q S H K F P V I L P<br>I E V P E V V K K I K E I P L F I I L D S G M K S E I K N F A V N Q A R N F<br>I E S V E S S L T N K M N S E A K K T H A N D K E G L G I L N F R S R I V<br>V I T E D L N S K R G I I Q V I E E A S A M M K G K E L G Y A Y I A T R N<br>K L S S E K N Q D V K K K L F H S R V I S Q V V N E D A L R N K R D K W<br>N R N K L D L K V V Y R V L F Q I L S K L G I Q Y T V L N Y Q L N Y D F<br>V I G I D V T P M K T S E G F I G G I M V M F D S E G Y L R Y I I P I K I G<br>E Q R S E T V N M H Q F Y K E C V D V M R H F K I Y L I N K K I L I L R D<br>G R I T E D E L E G L K Y I S R K Y N I D I V S F D C I M N H P L R I F A N<br>E R M Y F N F G G A I Y Y I P H E L R D S R G T P I P I K L A E R K E I K<br>Q G K V K R R S I T K E D I L D I L I Q T Q L N L G R I S S D Q Q L P A P V<br>H F A H K F A D A V R N D W H I D I Y F L S N G F L Y F V
Pf-AP2	M K A K V V I N F I K I N Q K I I P E R I Y A Y K L F Q D P E E E L K K V<br>G F T L H K L A D Q N C G I V V D P E N L I I A T T K E L E F E G E F I P<br>L G E I S F E E L K D S D Q A E L I L T L L Q E R G I G Q K N L S K L L Q K<br>Y R K P E V V G N F Q V V P L T N C D V I K H E E D F Y L V I H L K H I I<br>Q S L K T L W E T C N K N P E H L I E F L Q Q H K E N L Q L Q N I I S P Q<br>K T V Y K P A Y L K F N E K P E L N H N Q Q V V Q F F F Q Y H I E R F Y<br>N V P I G I L N F F R K F G Q V D T K L P A V V A K L A S E V K L T H D<br>K E A Q C L P Q L V V P Q F N A Q K L D S D L N E Q I E I F T K L E P D E<br>R R E L L Q N I L A Q I D S D V I D R T L D K I E C M Q I S Q E L E H I I K<br>V K D T K G N S V P I S Y I T V E F S K K I D Y D E Q E Y R F P I V L P Y<br>K V P Q L L K K V R E I P L F L I L D S G L E A T I S D F A T N E F K E L I<br>K A L E H T M A K E Y S S L A K M A K S N D H E G I G W L C I R A R I I T<br>I T E D L A S Q R G K D H I V N S V S S I M Q G Q E L G L C L I Y I K D K L<br>S A E K I E D I K R K M F Y L N I I S Q I I N E D T L K D Q R D R Y N K N R<br>V D L Y F V H N I L F Q V L S K L G V G Y Y V L D Y Q F N Y D Y I I G Y D<br>L A P M K K S K G L Y G G S A V M F D S Q G H L K R I V P I R I G E Q R S<br>E S V D M N E F Y K E L V E L L K K C K F K F T D K K V L L L K D G R I<br>T Q D E R E G L K Y I S K K F N I Q V D T L N C I S D H P I R A Y A N M R<br>F Y F N L G G A L Y L I P T K L K E A N G T P L P L K I A K R M V I K D G<br>E V E W E T I T R K D I T N I F V L S K L N N G T I S T D M R L P A P V H<br>F A H R F A E A I R N D W K L K R Y F L S W G F L Y F V
Pf-AP3	M K A Y V T V N L I K I N T K I I P N K I Y V F K L F Q D P E Q E L K Y V<br>G F T I Y K L A Y E N V G I V I D P K N L I I A S T K Q L E Y I G E Y I P E<br>G E I S F S L L K N S N Q S K F V L Q L F Q E H G I G Q F T L S E L L R K Y<br>K K P K T F G D Y E V I P S V L L N V I K E N I D Y Y L V C D L K H K I Q<br>A L K T L A E F V D R D P E L L E E Y I K V H K E T L D L K D I L S P Q R<br>T V Y K P C F Q E F Q I K P E L D H N H N V I Q Y F F K Y H I E R Y W N<br>V P Q A E K N F F Y K F G K L D L N Y P A I L A K F A T Y D R E A L D R<br>R V F L L P Q L V V P N F N A T Q L N S D I T K E I Q I Y T K L M P L Q R<br>R K L L E D I L A K I N S T I V D K T L S E I E V E K I S F E L E N L I K I K<br>D N K G N S V P I T H I T D L S S E E L D E S D K S N K Y P V I L P Q K I P<br>E G F K K I R E V P M F V L L D S G L K S D I Q D F A V N E F K Q L I K A<br>L E H T L T E K Y L A I A K Y A H S N N K E A I G F L D F R T K E E V I T<br>E D L T S Q R G I V E V V N S V S A I M K G R E L G L A C I K V Q D F L S<br>S Y R F N D I K R R L L H L N I [illegible] S Q V M N E T T I K N H Q D K F N K D R<br>L D N K V R H N I L D Q I L S K M G I A Y F V I D Y E L N Y D Y I I G I D V<br>A P L R D S K G Y I G G S A I M Y D S D G Y M K R I I P I K V G E Q R S E<br>T I D M E E F F N E L I D K L K E L F I Y L N N K K I L V L K D G R I T E D<br>E R Q G L K Y V S K L Y N I E I L T M D V I R I H P T R A F A N M K L Y Y<br>N F G G S I Y L I P H K L Q Q Q E G T P I P L K I A K K Q I I K N G K V F R<br>K S I T R Q D V E D I F I L T R L N H G L V S S D M R L P A P V H Y A H K<br>F A N A I K N D W Q I I I E E L D Q G F L W F V

Table S20: Sequence specifications of Pf-APs (4-6).

Protein	Sequence
Pf-AP4	M K A K V K I N L V K I D D E I I P D R I F V Y R L Y Q D P E N E N K K T<br>G F T I E K L A Y E N S G I V V D P E N L V I A T T K I L I F D G E F I P E<br>G E V S Y S Q L R N S Y Q A K L I L R L L K Q H G I G E Y A L S Q I L Q Q<br>H I K P K T F G T Y E V V P S V K L S V I K N N D S L Y L I V H L I H L L E<br>S L K T L W Q L V N Q D P L I L Q E F L E T H R E T L L L K D I A S P L K<br>T V Y Q P A F E E F T N K P L L D Y N Q D I V Q Y Y Y N H H I E R Y W N<br>T P L A I N E Y F R Q F G Q I D T E Y P A I L A R Y A S M E E H T T D R R<br>M Y L L P Q L T V P K Y N A T Q A S S T L C K K V D Q I T K L E P L E R<br>Q R L L E D I L A Q I N S D I I E K N L N Q I E V E L I S K E S D D I V K V K<br>N D K G N S V P V S E L S V E S S Q K L N H A D Q S D E F P V I S P D R V<br>P E K I E Q I R D I P M F I V L D S G L E S D I Q D L A V N E F R E L I K S<br>L Q S S M A M L Y N A E A Q K S R S N T K Q G L P Y L T F K S K Q K V I<br>N Q N L C S N K G I I E I V T V V S A I M K G K E L G L A F I S I E N K L S<br>A N K Y E N I R K R L F N C N I V T Q I I N E D F V K N E R D K Y N R N R<br>F S L D T V Y N I L L E V L S K L G I K F T T I D Y H L N Y D F I V G I D T<br>V P M K D S Q G Y I G G S A V M F D S E G H A Y K I V P I Q V G E K R S<br>E T V N M E Q Y F N D M V D D W K Q L H I K M D N K K V L I M R D G R<br>I T E A E Q E G L R F I S R E W N I Q I V T L D V I K D H P F R I F A N Q K<br>L Y F N L G G A I Y L V P T K L K N S K G T P I P L K L S K Y T T I K N G<br>K I K R M S I T R Q D V L D I L I L T R L N Y G Q I S G D L M L P A P V H<br>Y A H R F A N A I R N L W K I K E F L L E L G C L Y W V
Pf-AP5	M K A Y V V V N L V K I N Q K I I P E K I F C Y R L F Q D P E E E L K K D<br>G F S I E K L A D D N N G I V V N P E N L I I A T T K D L K Y D G Q Y I P<br>E G K I S F S E L R N G Y Q S E Q V N Q L F N L H G I G E Y Q L S K I L Q<br>K F R K P K T Y G I F L V I P S V E M N V I K E N N D Y Y L V V H I I H Q I<br>Q S L K T A S E L V N R N P K K L V L F V Q Q Y R E N L N L K D V A S P<br>E K T V Y K P C Y E E Y N D K P E L D Y N K N V I E Y Y F N H H L E R Y<br>Y N T P Q A K D E L F R R Y G F V D T H E P A I V A K F A N E I H T S H<br>D Q R V H Q L P Q F V I P T Y N A E L L D S E V S F E I L E S I E L K P E E<br>R Q K L C E D I L S E V D S T I V D K D L N Q I E C D K V D E E L D N I I K<br>I Q D D K G N A I P I S L I S V Q A S K F N N E D D W A Y K F P V I L P Q<br>K I P E A F K K I R E I P I Y I L I D S G L E S D I Q D F A I N C F M N L I I<br>A L R F S L C R Q Y N S E A K K A F S D V K I G L P L L N F R S H Q E V I<br>S Q N L N T D R G I V E I V D T V A A I M Q G K E L G L A F I S I E N K L S<br>D Y K L E D I R K K L F N M N I I S Q V I N E N V L R N H R S K R D R D R<br>L D I H T V Y H E L E Q I C S K L G V K Y Y H L D Y E F N Y D Y I I G I D<br>A A P M K N S K G H I G G S A I I F D A E G Y L K K I M P V K I G E L W S<br>E T V N M N Q F W R D L V D D L R K L H I R L D N K K I L F I R D G R I T<br>K D E E Q G F M Y I S R K Y N M E L I T M D V I I D H P L R V F A N M S I<br>Y F N F G G S L Y L L P T K L R D A K G T P I P I K L S K Y Q I I K D G K I<br>Q K K S I T R R D V C N I L I Q T K I N Y G E I S A D L R L P A P V H Y A<br>H R F A E A V R D E W T I N I K L L D Q G F L W F V
Pf-AP6	M K A Y V V I N F V K I D T I I I P E K I F V Y Q L F Q D P E E E L K K T<br>G Y S I W K L A K L N V G I V L D P K D L V I A T T K Q L E Y E G Q F I D<br>Q G E I S F S E L K N S N Q S Q F I H R I L N E H G I G E K N L S Q L L R E<br>Y R K P K T F G N F K V V P D V E M T V I K C N N D Y Y L V T H L I H R<br>I E G L K T L A E L V D K N P K L L E E F L Q I H L E T L M L K D I A T P<br>E R T V Y K P C F E K F T N K P L L N Y N E Q I V E Y W F N Y H I E R Y<br>W N L P I A I L E F F K K Y G E V D T K Q P A L I A K D A T E V K E F L N<br>K E V Y A L P Q L V V P E F D A E D M T D D I E K E I K D Y S K L E P E<br>E R K K I L L T I L S K V N S D V I D K N L E E I E M E K I S K E M D H I I<br>K V E D D K G N A I P I S Q L S V R D A E E N N F N D Y S Y K G P V I T P<br>I Q V P E A L K K I R E V P F F I V L D S G L K S E V Q N L A V D E F K S<br>L I E S L E F S I T K E E L S E A E K S R A N N K I G L P F F T F R A K I H<br>V I T E N L T S D K G I Q E I V T T V S A I M K G K L L G L G L V A I R D<br>K L S T E R K S D I K R R L F H [illegible] N L V S Q I T D E D V L K N Q R D K Y N<br>W D R L D L K T R Y N I L F Q V L S K L G V K Y F I L D Y H L D Y D F I I<br>G I D V T P M R K S Q G Y I G G A A V M Y N A E G Y L Q K I I P I K I G E<br>Q R S E S V N M I N F F F E M V D H F K Q L K I E L E N K K V L I L R D G<br>R I T D D E E Q G L K Y I S K K Y N I E V L N L D V V K D H P L R A F A N<br>M K R Y F N F G G A I Y L V P H K N R L S E G T P V P L K L A E R S I I K<br>N G K V R R E T I T K N H I E D I Y I A C R L N Y G R I S S D Q R L P Y P<br>V H F A H R Y A N A I K D N W K I N R Y D L A E G F L W F V

Table S21: Sequence specifications of Pf-APs (7-9).

Protein	Sequence
Pf-AP7	M K A R V V V N L V K I D N K I I P E K I F L Y K W K K H P E K E L K K<br>V G Y S L W R L A F Q N V G I V V D P Q N L I V A T T K Q L Q F E G L F I<br>P E G E I S M S Q L M N D Y Q S E L L H Q L L N K H G I G Q K S L S N I L<br>Q K Y E K P R T V G D Y E V V P F V N C N V I K Y N E D Y Y L V I H I K<br>H R I R S L K T L D E I V D K D P K K L V E F F E K H R E N L E L K N I S<br>S P E K I V Y K P C F E E H N N K P K L D Y D Q D I R Q Y F Y H Y Q V K<br>R Y W H T P I A L Q D F L R K F G K T D M H Q P S L V A K Y A S K I K T<br>D M D Q M A Y L L P Q Y V T P T F D A Q E S T S V I D K E V L I F T K L<br>K P E E R K K L L Q N I L A E V D S T I I D K N L S Q I E C E K I S K E L E<br>S I I R V K N D E G N S I P I T D L D V K S A K Q L N Q D Q H S Q K A P V<br>V L P H H V P K S F K E I R Q I P M Y I V L D S G L K S W I Q N L A T D E<br>F K S L I E S L E T T F S E E S N A I A K Y A R A T N K Q G L P S L N F K<br>D K I K V I T E D L N S N K G K V L V V T T I S A F M Q G K E L G F A F V<br>A A T D E L S T E K I E D I K R K L F H M N I I S Q V T N E D T L R N R R<br>D K F N W S R L S L Y L V Y H L L F Q V L S K L G V N H F V L D Y Q L N<br>Y D Y I I G I D L S P L K K A E G H Q G G S A V M F D S H G Y L K K I I P I<br>K I G E L R S E T V D M H E Y W D E L V T E F R E L N I Y L D N K K V L<br>L L R D G R I T E E E K K G L K Y I S E K F N I E I I T M D I I K N N P V R<br>V F A N E K M Y F N L G G A M Y L I P T E L R L S Q G T P V P L R L S K<br>E E V I K D G K V E W R S V T K N D I L D I L F A T K I D Y G S I S A T M<br>R L P A P I Y W A H K F A N A V R D Q W K I K H E A L E E G W L W F V
Pf-AP8	M K A R V V V N L I K I D Q T I I P D K I F A Y R L H K D P E E D L M K D<br>G F S S E K M A D E N N G I V V N P E N L I I A T T K Q I E Y E G E F E P<br>Q G E V S F S N L K D S F Q S E F I L S R M Q K H G I G E K E L S E I F R K<br>F R K P K T V G N Y Q V T P S I E L D V I Q L N E D Y Y L V I H I I Y Q I I<br>S E K T L F Q L V N K D P M L M E D F L E I H K E T L E L K D I A T P R Q<br>T V Y K P L F E K Y N D K P L L D Y D E N I I E Y Y Y N H Y I L R Y W N<br>T P I G K E N F K R K F G K V D F K Q P A I L A R F A N H I Q I A M D K<br>K T H L L P Q L T V P V F N A T K L D D D V N K K V L Q F S K L K P N E<br>R K L L L T N I L A E V D S D I I D K T L E E V E A E K I S Q Q L D N I I K I<br>K D D K G N S I P I S Q F T I D E S E E E I K D D K S Y K Y P V I E P I R V<br>P E K L K Y I R E I P I F Q I L D S G L Q S E T S D F A C N Q A M S L I E S I<br>E F S L S N E Y N S I A K Q A H S N D Q E G L G T L N F K S K D M I I T E<br>D M N S N R G I E L I V K E V S T L M E G K E L G F A F I F V H D K L S A<br>E K F S E I K R R C L D T N I I S Q I L N E T F L K E K R D Y Y D R D R L<br>T E F F R H H V L H Q V L S K F G I K Y F V L D Y K F N Y D Y I I G I D I S<br>P L K K S K G F I G G S A V M F D S Q G Y L K K I V P I E V G K Q R S E T<br>V N M H E F I M E L V D E W K R L K I E L E N K K V L L L R D G R I T E<br>N E K E G L Q Y V S K K Y N I E I L T L D V V S N H P L R A F A N R M L Y<br>F N L G G A D Y L I P H E L K E S Q G T P I P V K L S K Y Q I I K D G K V<br>Q R Q S T T K Q D V E D I F V L T K I V F G Y V D A D M K L P A P V Y F<br>A H K F A N A I R N E W K I N K E K L S H G A L W Y V
Pf-AP9	M K A Y V V V N L V K V N K K I I P E K V Y Y V Y K F F Q D P Q L E L Q R<br>V G Y S I Y R L A Y N N N G I V V D P E N L V I A T I R K L E Y E G E F I P<br>L G Q I S F S Q L Y N A D Q A K L I K N L L K E H G I G E K T L S N I L K E<br>F K Q P K T F G D Y K V V P F V I M N V V K H K E A F Y M V L H L I H N<br>I K S T K T L A E L V N R N P K E L I T Y V M Q H K E T L L L K D I S S P<br>E R T V Y K P N F L K H N D K P M L N Y N E D I I H F Y F N L Q V Q R Y<br>W N T P I A K E N F F R K F G K L D M E Q P S I V A R L A Q L V E T S K<br>N R E V Y L L P Q F V I P Q Y N A E Q L N T D V D K K V Q L F S R L M P<br>Y E R K Q L L E D M L S Q I D S D I I E K K L N K V E C N K I A K E M D N<br>I L R V K D D Q G N E I P I T E L E D N S A E L L D W T D E A E K A P D I<br>T P Q K V P E S F K K I R K I P L F I V L D S G L K S D I S D F A T N E F K<br>N L I Q S I E Q T M A Q K Y N A V G Q K A Y S N D Q E G L P F L S F R A<br>K I E V V T E D L S S D R G L V E V I T V C S A R M K G K E L G M A L I C<br>I K N R L S A K K F K D L K R R C L E M N V I T Q I I N E D F I K N Q Q D<br>R Y N K D R L A L F F R Y N E L M Q V L S K L G V K F Y N L D Y E F D Y<br>D Y V I G L D V S P M K R S K G F I G G T A V M Y N S D G Y L K R I V P I<br>K I G K L R S E S I D M I Q F Y K S F V D V L R E L H I K L D N K K V L L<br>L Q D G R I T Q N E E E G M E Y V S K K F N I E V V T L N V I K N H P L R<br>V F A N K E M Y F N L G G S I Y L I P T Q L R E S Q G T P L P L K L S R Q<br>Q E I K N G E V F Y Q S W T Y Q D V T D I H I L T R L N Y G K I V A D M<br>K L P A P V H Y A H K F A N A V R N D W K I N R Y L L D E G F L F F V

Table S22: Sequence specifications of Pf-APs (10-12).

Protein	Sequence
Pf-AP10	M K A Y V S V N L V K I N K K I I P D K I Y L Y M L F Q N P E K E L K K V<br>G Y T V Y R L A Y D N N G I V V D P K N L I V A T S K N L Q F E G E Y D<br>P E G E I S F S E L K D A N K S K L V L N I L E E K G I G E Y E L S L I L K<br>K Y W K P Q T F G D Y Q V I P T V Q C N V I K H D D D Y Y L V I D I K W<br>R I E S L K T L A E L C N R D P F L L E K F F I Q H R E L L E L K D I V S P<br>E K V V Y R P L F Q E N N N K P L L D Y D Q N I I Q Y Y Y Q F H V E W W<br>W H D P E A L E N F F K K F G E L D T K Q P A I V A R F A N K E K I A L<br>T Q Q V Q M L P Q F V V P T Y K A D E L V S T Q D Q Q I Q E Y T K L L P<br>E E R Q E L L Q S I L S Q I D S D V I D R T L D E I E C Q K I S K Q T E N L<br>L R V R N S Q G N S V P I S E F S D K A S E E D L F S D Y G H K Y P V I L<br>P V K V P D T F K K I R E I P L F L L L D S G L L S D T Q D F A T Q Q F R<br>D L I K A L D N S L S N K I N S D A E K A L S N N M E G M P F L T F K G K<br>N Q T I T Q D L N S E K G I V Q I I T C V S A I M K G K E L G L A F V A V<br>K N K L S N E K E E D V R R R L F D Q N I V T Q I V N E T T L K N Q R D<br>K Y N K N R L D L N T V Y N I L L Q V L A K L G V W Y Y V L N F H F N Y<br>D F I I G Y D V S P M K K R E G Y V G G L A V M Y D S E G Y L T K I I P I<br>R I G E S R S E S I D M N E F L K E L V T T F K K M K V H L D D K K V L I<br>M R D G R I T E E E E E G L K Y V S K K Y N I V V I T M D C I S E H P L R<br>V F A N M N M Y F N F G G S M Y L I P T K L E F S E G T P I P I K L S R R<br>E V I K N G K I E R K S T T K K D V L D I F F L S K I T H G Q I N G R L R L<br>P A P I H Y A H K F A N A I R Q N W K V I E E L L S E G F L Y F V
Pf-AP11	M K A K V T V N C V K I D T T L I P E K I Y V Y R F F N D P E E E L L K V<br>G Y S I E R L A Y Q N C G I V I D P K D L I I G T T K E L H Y I G K Y V P L<br>G E I S F S Q L K D S I Q S D A I N Q I F N K R G I G E R E L S K I L Q K Y<br>F K P E Q V G N W E I V P S V Q L S V I K H D Q S Y Y L V I H L I F D I Q<br>A L K T L W Q L V N R D P I L L V E F L E E H K E D L E L K D I A S P E R<br>T T Y E P S F Q K H N D K P Q L N Y D I N I V Q Y W Y D H F I E R Y Y N<br>T P I A I N E F Y R K F G K I D T Q Q P A I L A R F A T Q I E I S L D Y L Y<br>K L L P Q L V I P L F Q A E E L N S D Y T K E V L I F T K Q E P T E R R K<br>L L E D I L A E I D S D I I N R T L D E V K T Q K I S K E T D N I I K I K D N<br>K G N S V P I S K L D V E S S Q E T I K D Q W S D Y F P V I T P I K V P Q<br>V F E K I R E I P M F I I L D S G L E S D H S N L A T N C F K Q L I E S L V<br>Q S L T F K Y N A L A H Q A R S N D R E G L P F L T F R S K E L V I T E N<br>L N S T K G I D E V V D T C A A I M Q G K E L G L A F I L I N N K L S A E<br>K Y N D I K R K L F N L N V I S Q V I N E T V L R N M R D K Y D R N R L<br>D Q L I I H Y L L F Q V L S K L G V K Y Y V L D F Q F N Y D Y V I G I D L<br>S P M R K T K G D I G G A A V I Y N S Q G W M K K I I P I R I G K L R S E<br>T V N M D D F F Q E L I T K F R K L N I R L T N K K V L F L R D G R I T E<br>N E K Q G L K Y V S R L Y N I D I M T F D C V K N H P I R A F A N M K M<br>Y F N L G G A M Y L I P H E L K E S K G T P I P L K L A E K S V I K N G K<br>I E R E S I T K L D V E D I W I D T R L N Y G T I N A T M N L P A P T F Y<br>A H K L A N A I K N D W V I N L Y E L D E G F L Y W V
Pf-AP12	M K A Y V T I N L I K V N V K L I P N K I Y V Y R L F Q N P E E E L K R V<br>G Y S L F K L A Y Q N N G I V V D P L N L I I A T T K N L E Y E G K F V P<br>L G E V S F S E L K P G M Q C K L I L N I L K K H G I G E Y T L S K L L Q<br>E Y R K P K T F G D Y L V I P D A N C D V I K H N N D Y Y L V I H L I Y E<br>I E S L K T L W E L V N R N P K D L I L F L K T H K D S L L L K D I D S P<br>E K T V Y K P E Y E R H T D K P R L D H N K N I V Q Y H Y N H H I Q R Y<br>Y N V P I A I L E L F K K F G Q I D L K L P A I E A K H A N K Q E L T H N<br>E K F Y L L P Q F V V P L Y V A E D L D S D V N K Q T L I Y T K L Q P N<br>E R L H L L Q D M L A E I N S N I I D Q T L D E I Q C E K I S K Q M E H I I<br>R I K D D K G N T V P I S Q I T V H S S Q I Q Q E D D F S E K F P V I T P H<br>E I P E L F K K I R Q I P L Y I I L D S G L L S S T C D L A V D E A K N L I E<br>S L E Y S L A K R S N S L A R K A R T N D K E G L P Y L N F R A K E N V I<br>T E D L N S D K G I E V V V D E V S A I L K G K Q L G L A F I S A K D K L<br>S S E K L E D I K R R L F D L N [illegible] I S Q V I N E T T L K N E Q N F Y N K N R<br>L D I D L R F N L L Q Q I L S K L G V N F H H L D Y Q F D Y D Y I I G I D L<br>F P M K K S K G Y I G G A A V M F N A E G Y L R Y L V P V Q I G E L R S<br>E T V N M I L F W Q N L V T K Y R K L E I Y L D D K I I Y F L R D G R I T<br>E E E Q E G I R Y V S K Q F N I E V L T Y D C V K E H P T R V F A N M Q<br>H Y F N L G G A L Y L I P H R L R L S K G T P I P V K L S K Y R I I K D G<br>K V K K K T I T R E D V V D I L I L S R L N Y G D F I G D Q K L P A P V Y<br>F A H K F A N A I R N N W K I N K Q Y L A E G F L Y F V

Table S23: Sequence specifications of Pf-APs (13-15).

**Protein**	**Sequence**
Pf-AP13	M K A Y V T I N L V K V D T D I I P K K I Y H F K L F Q D P E E E L Q R D<br>G F T I W R L A F Q N C G I V I D P E D L I I A C T K E L E I I G H W E P E<br>G E I S F T Q L K D A M Q S E L L L N K F N H H G I G E F N L S I L L K K<br>Y R K P K T V G D Y E V I P S V E C N V V K H D E D Y Y M V V N V I H L<br>I K S I K T L A E L V D K D P Q K L E D F L E V H K E H L L L K N I V S P<br>E H T T Y R P A F L K H T N K P L L D H D Q N V V Q Y W Y H H H I E R Y<br>Y N Q P F G Q L E F F K R Y G K V D T E Q S S I K A K L A T K T K R S D<br>D K R V F N L P Q C V V P A F N A D K L T S T V A K E I N E F S K L W P<br>E E R K Q L L E D I L A Q I N S T I V D K N L V K I E V E K I S R E M D D<br>V I R I K D S K G N S I P V S Q M S V R G A E Q L T W S N E S D K F P V I<br>D P E Q V P E A L K K V R E I P L F I L L D S G L E S D T Q E W A T N E F<br>K E L I E A L Q Y S L S K E Y N A L A K R A Y S D D K D A L P F L N F K A<br>E I E T I V E D L G S L R G C E D V V D T V S A F M K G K E L G F A F I A<br>L K D K L S A E K I E D I K R K L F D C N V I S Q V I N E T T L R N K R S<br>K F D K D R L Q T F V R H N A L E Q I L A K L G I H Y F V L D Y Q F N Y<br>N Y I V G I D I T P N R R T E G H I G G S S N M F D A S G F C K K L I P I Q<br>V G E Q R S E T V N M H Q F F N Q A I D T F K R F R I Y L D N K K V L L I<br>K D G R L T E N E Q E G L E Y I S K K F N I D I V T F N V I I N H P I R V F<br>A N E R K Y F N L A G S L Y L I P F E L K K S E G T P I P I R L S K R Q V I<br>K N G K I Y R R S I T K K L V E D I F I L S K L N Y G S L S A E V R L P A P<br>V F Y A H K F A N A I R N D W K I N Y K L L S Q G W L F F V
Pf-AP14	M K V K V V I N L V K I N T K I V P E K V Y V Y R L I Q D P E E E L K K V<br>G Y T I E K L A D Q N V G I V I D P E E L V I A T T R E L Q Y E G E F I P L<br>G E I S Y S Q L K D S F K C Q F L E T L L N K K G I G E F V L S K I L K K F<br>R K P R T Y G D Y K V I P A T K V D V V K H N E E H Y L V I H I I H E I Q<br>S E K T L W E M V N R D P K Q L Q E Y L E Q H R R T L M L K D I S S P Q<br>K T V F Q P Q F E Q Y N N K P K L N H N Q D I I E Y H F N Y H I E R Y W<br>N T P I A I L D F F F K F G F V D T E L P S V V A K L A S Q E K F S L N K<br>R I P S L P Q F V V P L F N A E Q L D S V V A K E V L Q F V K L E P W E<br>R D E L L Q N V L A Q V N S T I I D K N L S Q V E C S K V S Y E L D N L I<br>K V R N D Q G N E V P I S L I D I E S S E E N N E D Q I S T K F P V I L P V<br>E V P E S F R K I R E I P L F I V L D S G L K T S T S E F A T N E F M E L I<br>E S L E H T L C F K L N A E A E M A W S N D K E A L P M F T F K S K I N<br>V I T K D L N S N K G V E L I V N T I S A M M K G K E L G L A F V V L K<br>D K L S S E K Y Q L I R K K L F H N N I I T Q I L N D T T L Q N Q R D R Y<br>N R N R L A I D T R H N I L L Q I L S K L G I S Y Y T L L Y K F N Y D Y V I<br>G I D I S P E K S S Q G D I G G A M V M Y D S D G Y I R K I I P I W V G E<br>Q W S E S V D M E E F V N N M V D T F K Q M E I K F E D K K V L I I K D<br>G K I T N S E R E G M E Y I S R K Y N I D I I T F D I I K D H P S R I Y A N<br>Q R M Y F N L G G A V Y L I P H E L K E D E G T P I P I K L A R R M E I K<br>N G K I K R K S W T R Q D V L N I W F L T R L N Y G E I T A E E R L P A<br>P V H Y A H K F A N A V R N T W K L W E E Y L A W G C L Y F V
Pf-AP15	M K A R V V V N L V K V D K T I I P D K I Y V F K L F Q D E E E E L K K<br>D G F T I E K L A Y E N V G I V V D P K N L V I A T T K E I E Y Q G E K I<br>P L G E I A F S E L K D S N Q A K L V L N I L N E H G I G Q K A L S E L L<br>Q K Y R K P K T V G N Y K V V P N C Q M S V I K H N E D L Y L I I N L I H<br>Q I E S R K S L A Q L V N R N P E K L I E W A I D H K K T L Q L K D V A S<br>P Q K A V Y Q P L F L K Y Q D K P L L N F D E D V V Q Y Y F N Y H L Q R<br>Y W N V P I G Q Q E F R Y R Y G Q L D S H Q P S I L A R L A N I I E D S E<br>C K Q V H A L P Q Y V V P Q F N A E E L D T D T D K E V L K Y T K K K<br>P Q Q R E E L L E D A L A Q I D S D V I N R D L D E I E T E K I S Y E M E<br>N I I K V Q N D K G N S I P V S Y L S V E S S N I E L W V N H D N R F P V<br>V L P F E T P E L F M R V R Q I P V F I L L D S G L L S D V Q E L A T N K<br>S K H L V E S L E F S M T L E Y S A Y S R Q A Q A N N K E G L G F L N F<br>R S K I L V I V E N L N S D R G I D E V I N E V S A L M K G K Q L G L A M<br>I A I K D K L S A K R I E D I K R R L F H I N V I S Q I I N E D T L K N Q R<br>D R Y N K D R L S L H V R Y N L L N Q I L S K L G V W Y T V L D Y E L D<br>F D Y V I G I D D T P M K Q S K G H I G G I A N M H N A Q G F C K R I I P<br>V E I G E L R S E S V N M N L F F N E L V S N F R E L Y I F L D N K K V L<br>L L K D G R I T D N M R E G L K F I S K K F N I E V I T M D V I I D H P A<br>R A F A N Q R R Y Y N F G G A L Y L V P H K L K E A Q G T P Q P L R L S<br>K E M V I K N G K S K R K E I T K Q D I S D I F F L S R I D F G Q V N V L<br>L R L P A P V H F A H K F A D A I E N N W K I K K L L L S Q G Y L F F V

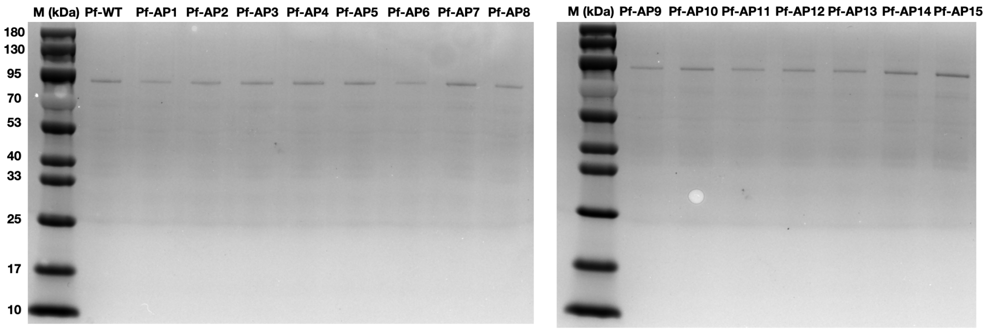

Fig. S90: The SDS-PAGE of Pf-WT and Pf-APs.

Table S24: SAXS data analysis of Pf-WT and Pf-APs.

**Protein**	**Rg (nm) (from Guinier)**	**Rg (nm) (from P(r))**	**Oligomeric state**
Pf-WT	2.90	2.88	monomer
Pf-AP1	2.86	2.89	monomer
Pf-AP3	2.87	2.89	monomer
Pf-AP5	2.93	2.90	monomer
Pf-AP9	2.95	2.93	monomer
Pf-AP13	2.92	2.93	monomer
Pf-AP15	2.93	2.82	monomer

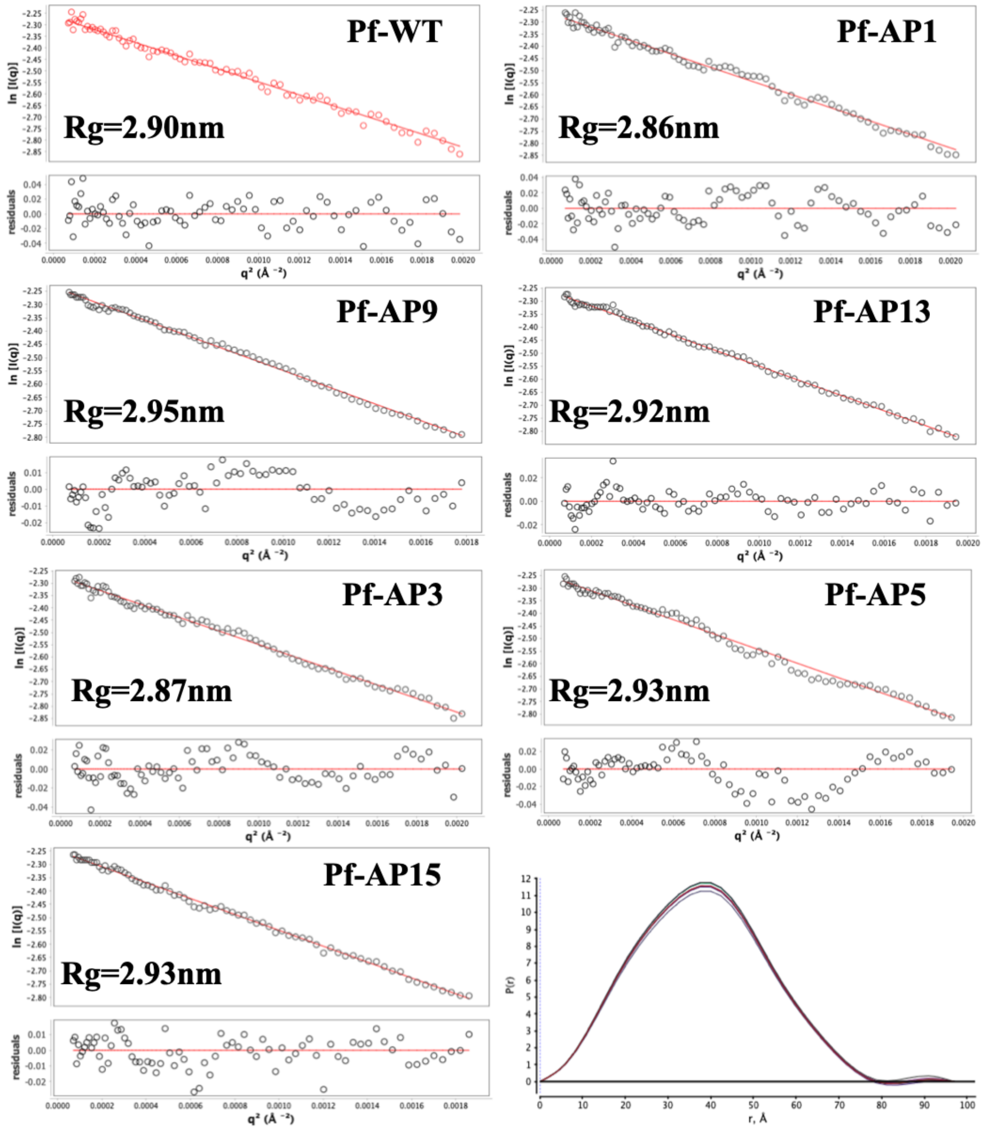

Fig. S91: Guinier plots of of Pf-WT and Pf-APs. The lower insets show the error weighted residual difference plots for the Guinier fitting. Last panel represents the pair distribution of Pf-WT and Pf-APs obtained from SAXS.

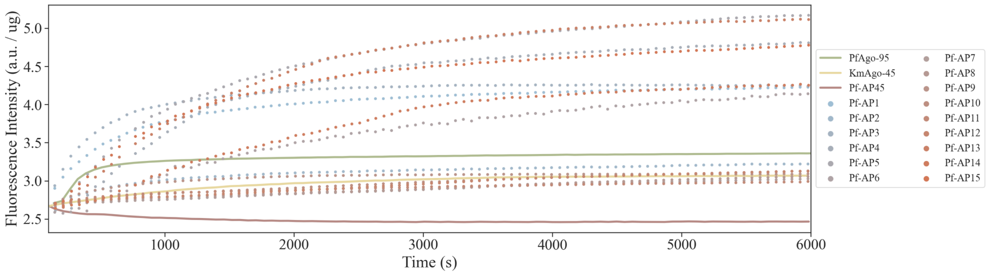

Fig. S92: The cleavage activity of KmAgo, PfAgo, and Pf-APs. The cleavage experiment of KmAgo, PfAgo, and Pf-APs is conducted at 45 °C, 45 °C and 95 °C, and 45 °C, respectively.

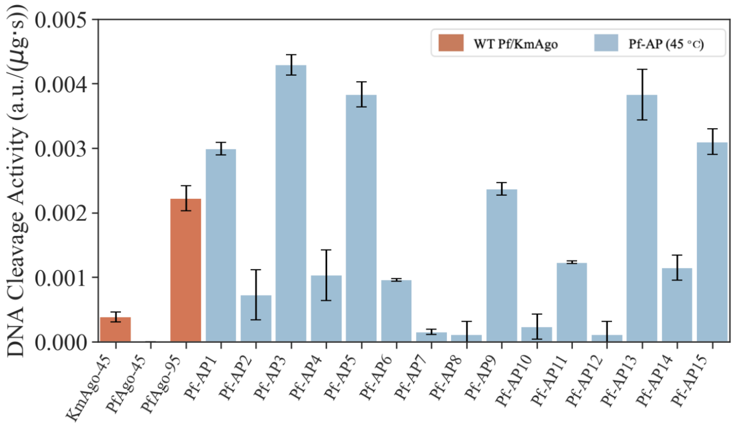

Fig. S93: The protein quantification of activity experiments of Pf-APs.

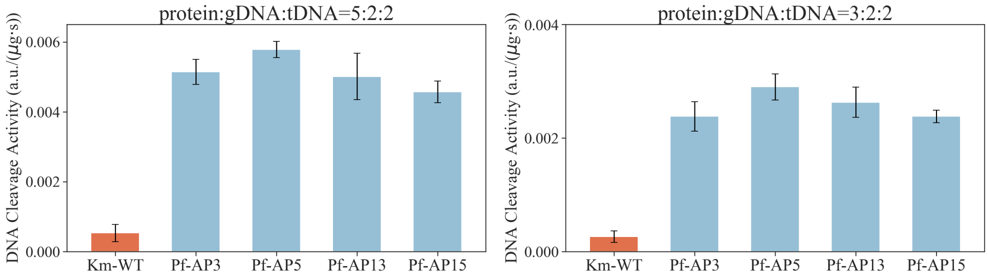

Fig. S94: The cleavage activity experiments of Pf-APs under different ratios of protein:gDNA:tDNA.

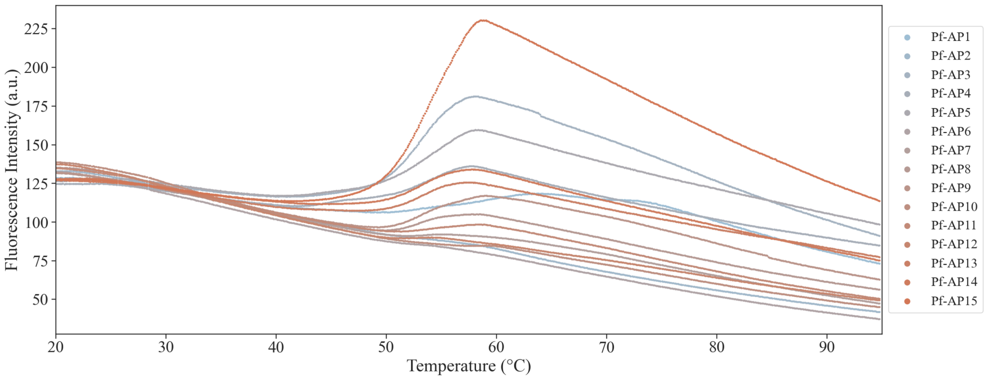

Fig. S95: Thermal unfolding curves of Pf-APs measured by DSF spectroscopy.

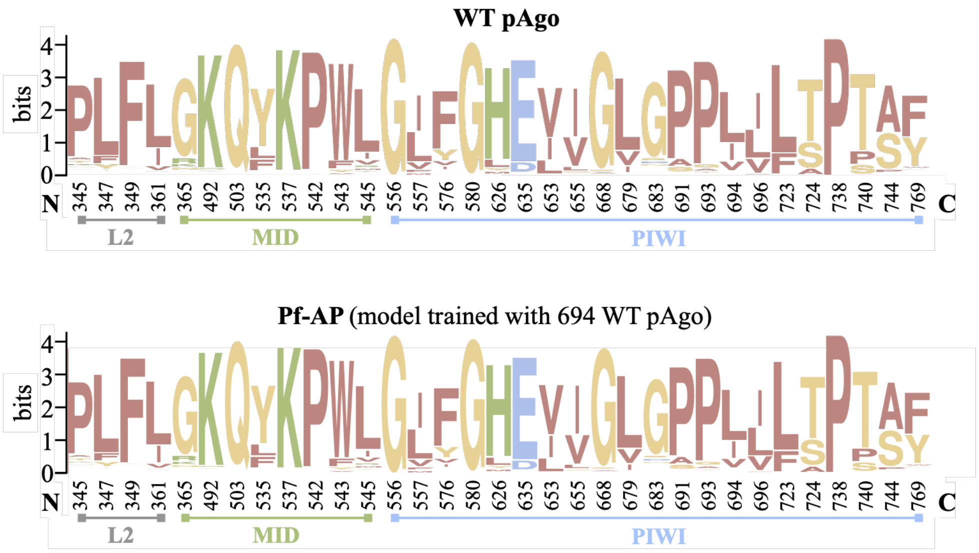

Fig. S96: Conservative patterns of Pf-APs on the 33 conserved sites aligned from the pAgo dataset.

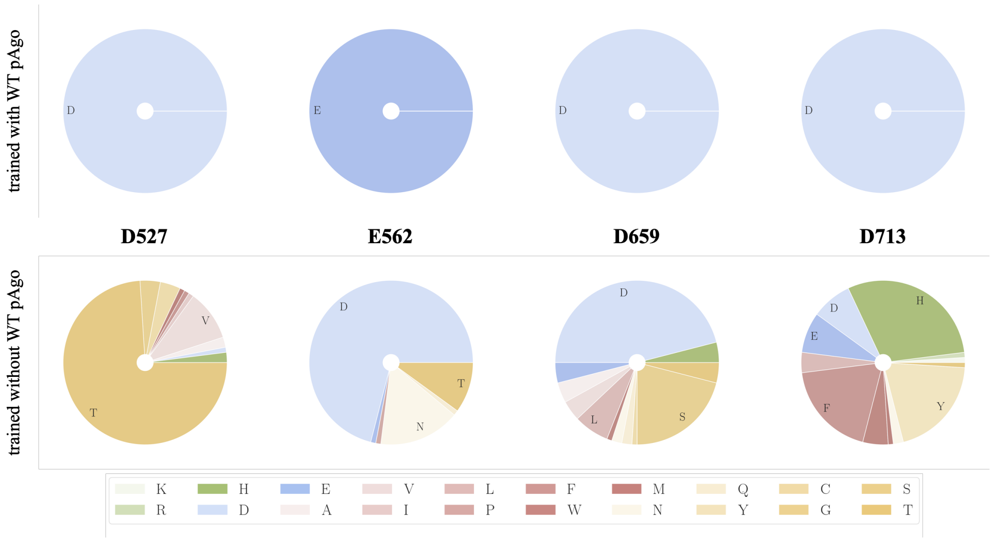

Fig. S97: AA composition of generated samples on the 4 sites of PIWI catalytic motif (DEDD) for KmAgo. Results are summarized for sequences generated by the CPDiffusion trained with (upper panel) and without (lower panel) the pAgo datasets. AAs with > 5% composition are labelled for clearer presentation.

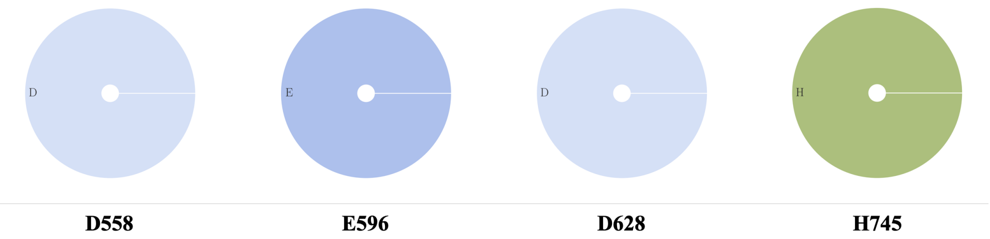

Fig. S98: AA composition of generated samples on the 4 sites of PIWI catalytic motif (DEDH) for PfAgo.

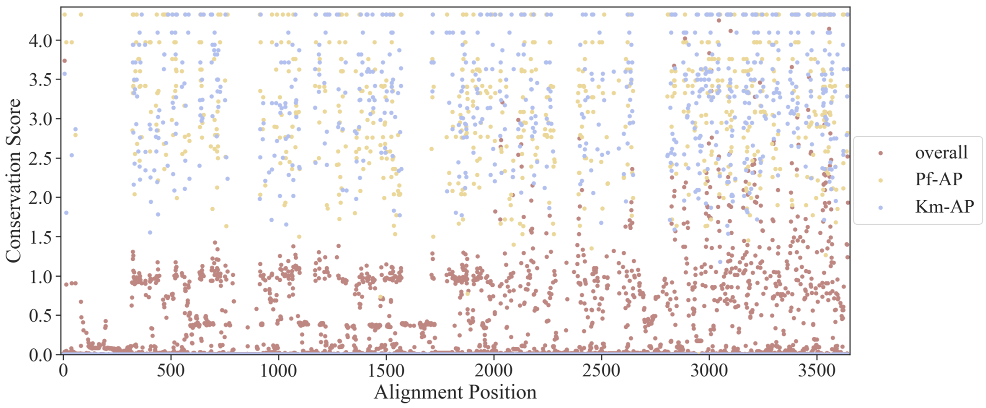

Fig. S99: Residue conservation scores for the aligned 694 WT protein, Km-APs, and Pf-APs.

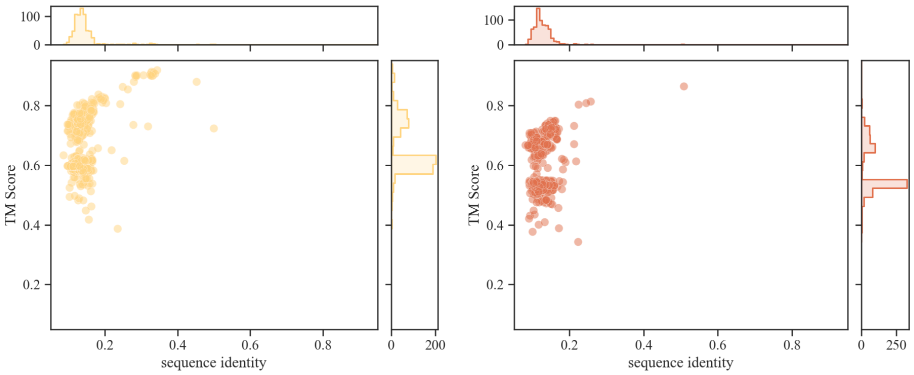

Fig. S100: Sequence and structure similarity of WT KmAgo (left, yellow) and WT PfAgo (right, red) with other WT pAgo proteins.

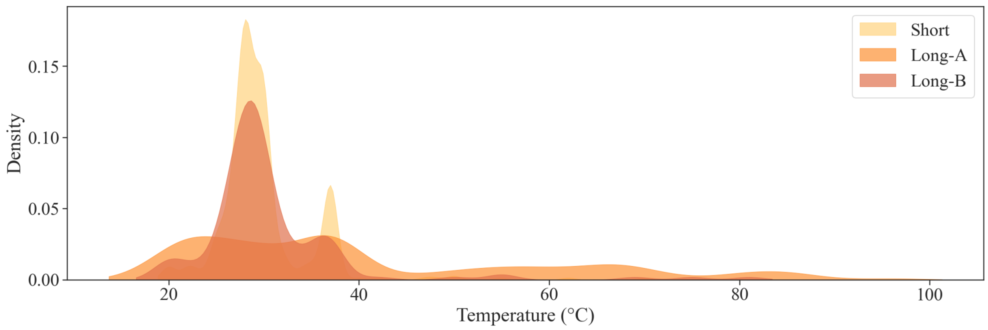

Fig. S101: The growth temperature of associated bacterial strains of the WT pAgo proteins, grouped into short, long-A, and long-B pAgo proteins. This metric is investigated to provide an indirect evidence of the optimal temperature for enzyme activity.

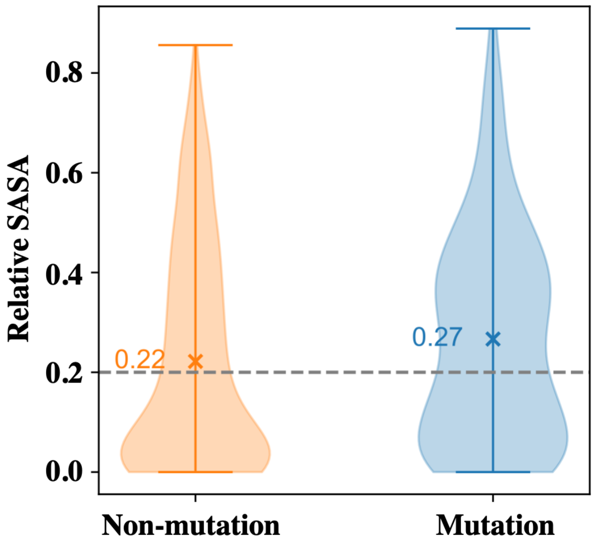

Fig. S102: The distribution of non-mutation sites and mutation sites of Km-AP23 compared with Km-WT.
